# Supplementary material for: TGFβ-induced long non-coding RNA LINC00313 activates Wnt signaling and promotes cholangiocarcinoma
Source: EMBO Rep. 2024 Feb 8;25(3):11. doi: 10.1038/s44319-024-00075-z (PMC10933437; doi:10.1038/s44319-024-00075-z)
Supplement: Supplementary file 1 — Appendix [file 44319_2024_75_MOESM1_ESM.pdf]

TGFβ-induced long non-coding RNA *LINC00313* activates Wnt signalling and promotes cholangiocarcinoma

Panagiotis Papoutsoglou[1,2], Raphaël Pineau[1],, Raffaele Leroux[1], Corentin Louis[1], , Anaïs L'Haridon[1], Dominika Foretek[2], Antonin Morillon[2], Jesus M. Banales[3,4], David Gilot[1], Marc Aubry[1] and Cédric Coulouarn[1*]

Table of content

Appendix Figure S1. *LINC00313* is induced by TGFβ signalling in TGFβ-responsive CCA cell lines. 3
Appendix Figure S2. Efficiency of SMAD silencing at mRNA and protein levels. 5
Appendix Figure S3. Gene expression profiling after *LINC00313* loss of function. 7
Appendix Figure S4. Specificity of *LINC00313* silencing in CCA cell lines. 9
Appendix Figure S5. *LINC00313* does not cause global alterations on chromatin accessibility. 11
.... 12
Appendix Figure S6. *LINC00313* affects HuCCT1 cell proliferation, migration and invasion. 13
Appendix Figure S7. Impact of LINC00313 on tumor growth and metastasis in a xenograft CCA mouse model. 15
Appendix Figure S8. The SWI/SNF complex catalytic subunit BRG1 regulates specific LINC00313-target genes. 17
Appendix Figure S9. Prediction of miRNAs that commonly target LINC00313 and TCF7. 18
Appendix Table S1. List of siRNAs 19
Appendix Table S2. List of DNA oligonucleotides (F: forward; R: reverse) 20
Appendix Table S3. List of primary antibodies 23

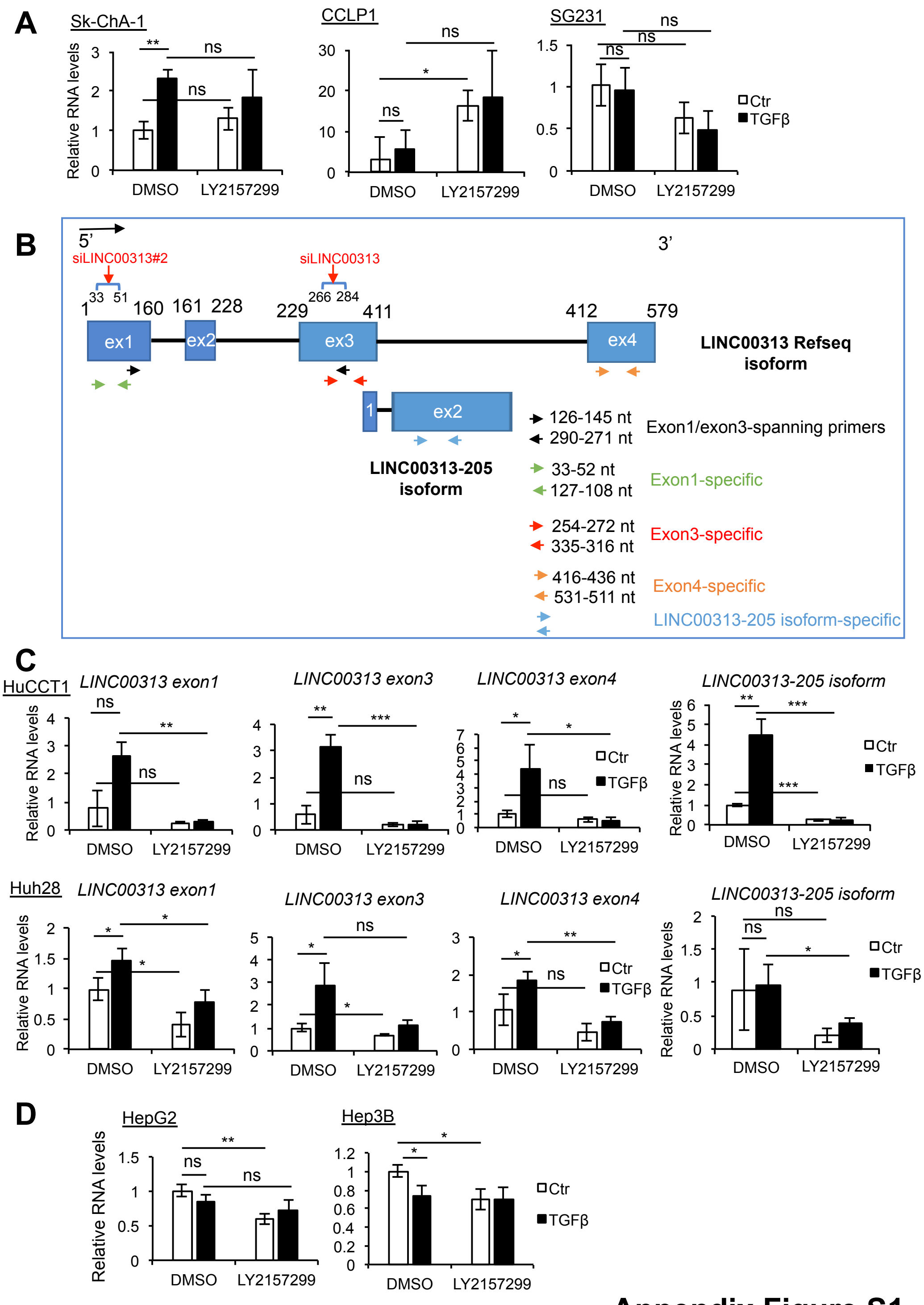

Appendix Figure S1

Appendix Figure S1. *LINC00313* is induced by TGFβ signalling in TGFβ-responsive CCA cell lines.

A) Real-time qPCR for detection of LINC00313 in eCCA cell line Sk-ChA-1 and in iCCA cell lines CCLP1, SG231 treated with the TβRI inhibitor LY2157299 or DMSO, and stimulated with TGFβ1 or BSA/HCl for 16h. B) Scheme showing the exonic regions of the RefSeq LINC00313 transcript, amplified by qPCR, using specific primer pairs. The regions targeted by the two individual siRNAs in exons 1 and 3 are also indicated. C) Real-time qPCR for detection of LINC00313 specific exonic regions in HuCCT1 or Huh28 cells treated with the TβRI inhibitor LY2157299 or DMSO, and stimulated with TGFβ1 or BSA/HCl for 16h. D) Real-time qPCR to detect LINC00313 expression in HCC cell lines HepG2 and Hep3B treated as in panel A.

Data information: In panels A, C and D data are presented as mean ± SD (n=3 biological experiments). *P≤0.05, **P≤0.01, ***P≤0.001, n.s.: not significant (Student's t-test).

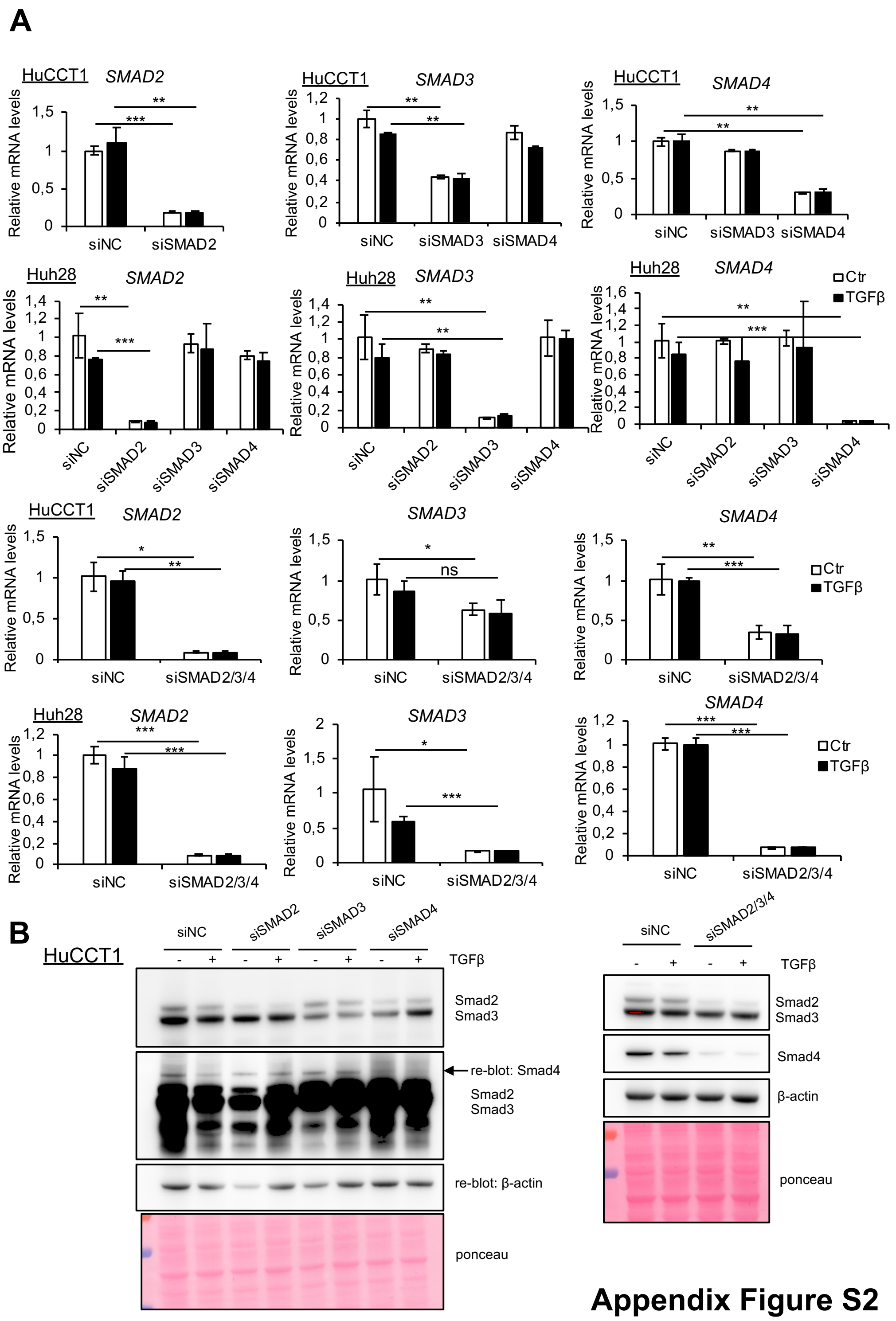

Appendix Figure S2

Appendix Figure S2. Efficiency of SMAD silencing at mRNA and protein levels.

A) Real-time qPCR to measure SMAD2, SMAD3 and SMAD4 mRNA levels in HuCCT1 and Huh28 cell lines transiently transfected with siRNAs targeting individual SMAD2 or SMAD3 or SMAD4 or simultaneously all three SMADs or a non-targeting siRNA (siNC) and stimulated with TGFβ1 or BSA/HCl for 16h. B) Immunoblotting measuring SMAD2, SMAD3, SMAD4 and β-actin protein levels in HuCCT1 cells transiently transfected with siRNAs targeting SMAD2 or SMAD3 or SMAD4 or simultaneously SMAD2/3/4 and in the presence or not of TGFβ or BSA/HCl for 16h. Ponceau staining of the same blots is also shown.

Data information: In panels A data are presented as mean ± SD (n=3 biological experiments). *P≤0.05, **P≤0.01, ***P≤0.001, n.s.: not significant (Student's t-test). In panel B the experiment was performed twice (n=2 biological experiments and the data of one replicate are shown.

A

FC≥ 2, p-value<0.001

Ctr group	Exp. group	Probes Up	Probes Down
siNC	siNC+TGFβ	575	699
siNC	siLINC00313	44	116
siNC+TGFβ	siLINC00313+ TGFβ	24	78

B

GO Biological Process (siNC vs siNC+TGFβ, 446 Up-regulated genes)

GO term	p-value	Adjusted p-value
regulation of cell migration (GO:0030334)	1.508e-16	7.696e-13
extracellular matrix organization (GO:0030198)	8.060e-16	2.056e-12
positive regulation of cell motility (GO:2000147)	9.283e-11	1.579e-7

GO Biological Process (siNC vs siNC+TGFβ, 591 Down-regulated genes)

GO term	p-value	Adjusted p-value
DNA replication (GO:0006260)	6.804e-34	3.472e-30
DNA strand elongation involved in DNA replication (GO: 0006271)	3.213e-12	1.491e-9
DNA biosynthetic process (GO: 0071897)	2.220e-16	1.416e-13

C

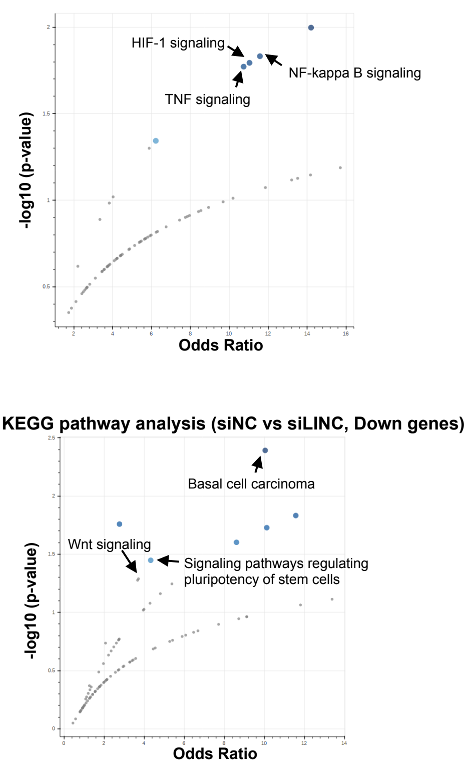

D

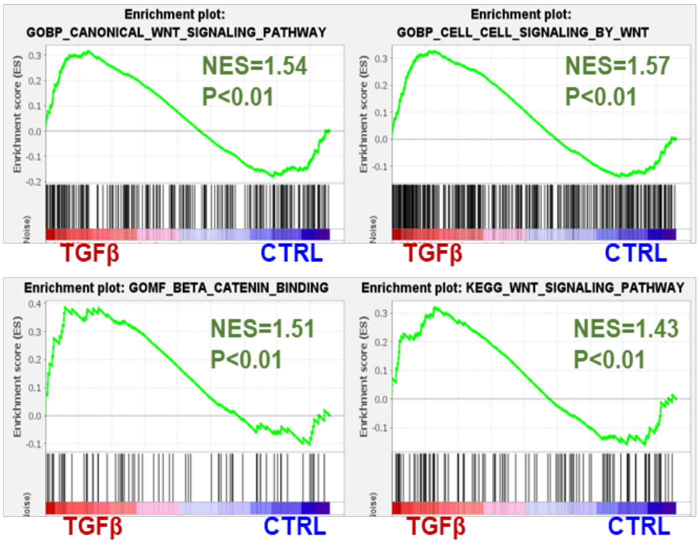

E

GOBP_CANONICAL_WNT_SIGNALING_PATHWAY

SYMBOL	ES	CORE ENRICHMENT
NOG	0.0120	Yes
TCF7	0.0242	Yes
COL1A1	0.0354	Yes
FERMT1	0.0467	Yes
JUP	0.0575	Yes
RBPJ	0.0664	Yes
SDC1	0.0785	Yes
WNT11	0.0833	Yes
WNT5B	0.0956	Yes
TMEM88	0.1033	Yes
GSK3B	0.1103	Yes
WNT7A	0.1198	Yes

Appendix Figure S3

Appendix Figure S3. Gene expression profiling after *LINC00313* loss of function.

A) Number of differentially expressed genes in HuCCT1 cells transiently transfected with an individual siRNA targeting LINC00313 or a non-targeting siRNA (siNC) and stimulated with TGFβ1 or not for 16h. B) Gene ontology analysis of the 446 upregulated and 591 down-regulated genes, in response to TGFβ1 stimulation. The top four statistically significant GO terms, related to biological process, are shown in the table, together with p-value and adjusted p-value for each GO term. C) KEGG pathway analysis of differentially up- or down-regulated genes in response to LINC00313 silencing in HuCCT1 cells. D) GSEA of curated Wnt signaling pathway signatures (from MSigDB) in the gene expression profiles of HuCCT1 cells treated with TGFβ versus control (NES, normalized enrichment score; $P<0.01$). E) Core enrichment genes for the GOBP_CANONICAL_WNT_SIGNALING_PATHWAY signature.

Data information: In panels B and C, GO and KEGG analyses were performed using EnrichR. The p-value is computed using a standard statistical method used by most enrichment analysis tools: Fisher's exact test or the hypergeometric test. The adjusted p-value is calculated using the Benjamini-Hochberg method for correction for multiple hypotheses testing. The odds Ratio is computed using this formula: oddsRatio = (1.0 * a * d) / Math.max(1.0 * b * c, 1) where: a are the overlapping genes, b are the genes in the annotated set - overlapping genes, c- are the genes in the input set - overlapping genes, and d- are the 20,000 genes (or total genes in the background) - genes in the annotated set - genes in the input set + overlapping genes. In panels D and E

GSEA analysis results are displayed as a normalized enrichment score (NES) and presented as an enrichment plot. The Kolmogorov-Smirnov test was used for statistical analysis.

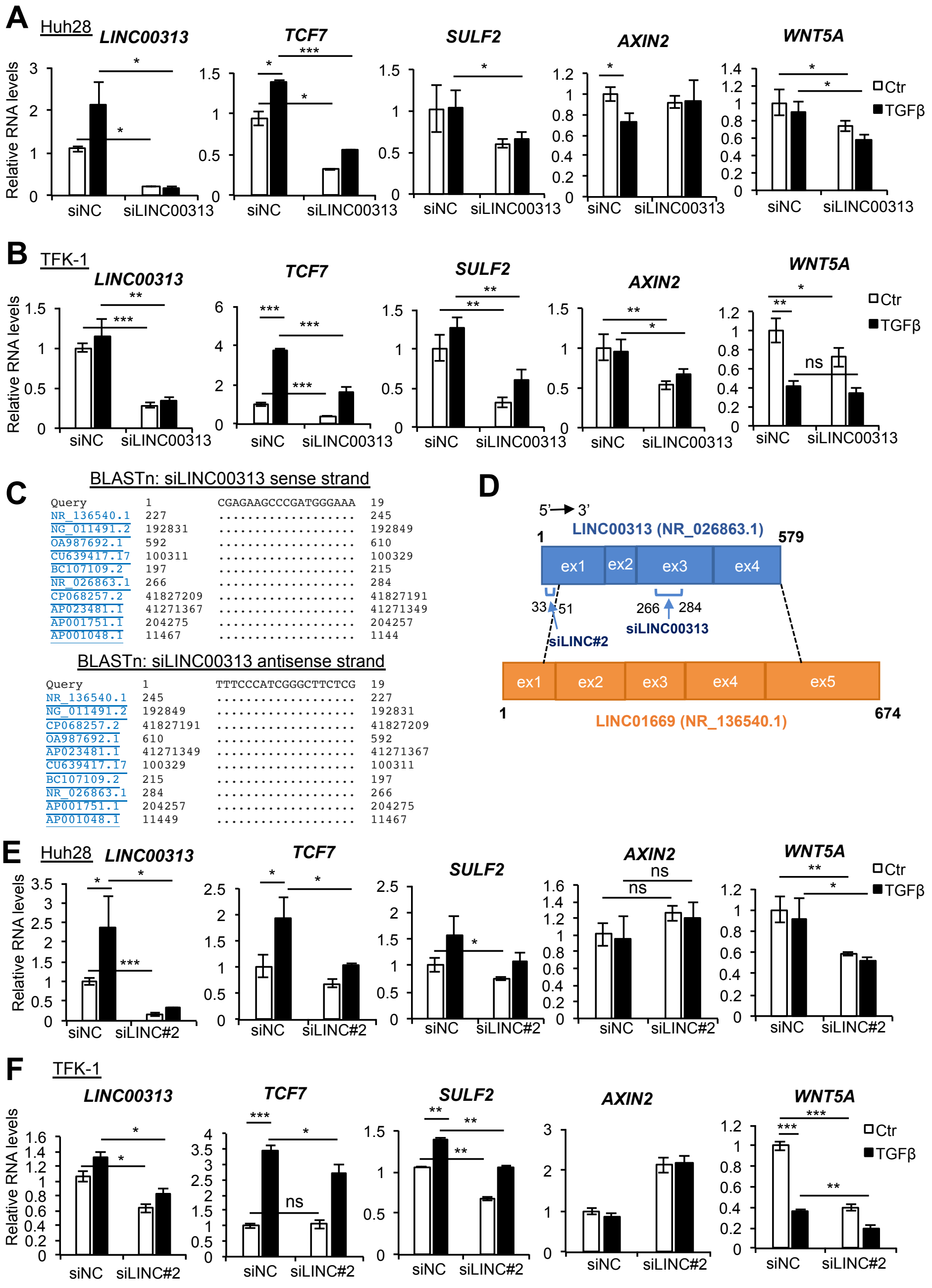

Appendix Figure S4

Appendix Figure S4. Specificity of *LINC00313* silencing in CCA cell lines.

A-B) Real-time qPCR analysis of LINC00313, TCF7, SULF2, AXIN2 and WNT5A expression in A) Huh28 and B) TFK-1 cells, transiently transfected with an individual siRNA targeting LINC00313 (siLINC00313) or a non-targeting siRNA (siNC) and stimulated or not with TGF β 1 for 16h. C) Nucleotide BLAST results showing top targets of the sense and antisense strands of the siRNA against LINC00313. D) Scheme representing the region shared between LINC00313 and LINC01669, as well as the regions targeted by the two individual siRNAs against LINC00313. E-F) Real-time qPCR analysis of LINC00313, TCF7, SULF2, AXIN2 and WNT5A expression in D) Huh28 and E) TFK-1cells, transiently transfected with a second individual siRNA targeting LINC00313 (siLINC#2) or a non-targeting siRNA (siNC) and stimulated or not with TGF β 1 for 16h.

Data information: In panels A, B, E and F data are presented as mean ± SD (n=3 biological experiments). *P≤0.05, **P≤0.01, ***P≤0.001, n.s.: not significant (Student's t-test).

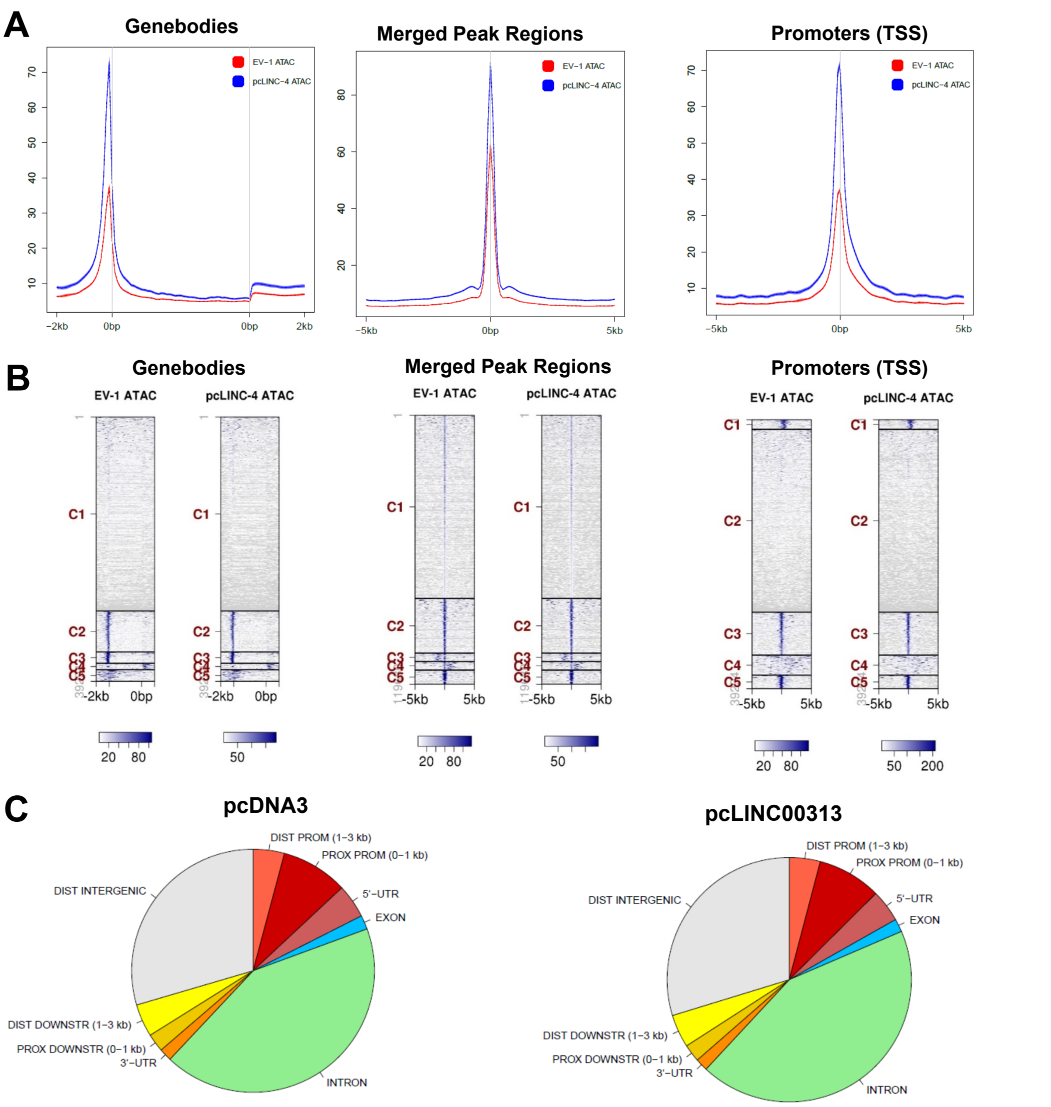

D

HOMER de novo motif analysis
Regions with increased peak signal in pcLINC00313

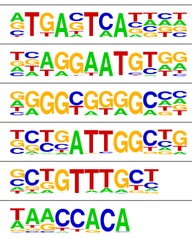

	Motif	p-value	% of targets	TF
1.		1e-186	25.24%	Fra1
2.		1e-45	11.35%	TEAD1
3.		1e-38	20.73%	KLF5
4.		1e-37	9.38%	NFY (CCAAT)
5.		1e-20	10.33%	Foxo1
6.		1e-16	39.71%	RUNX2

HOMER de novo motif analysis
Regions with decreased peak signal in pcLINC00313

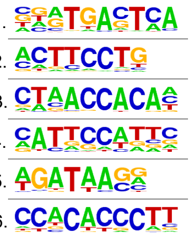

	Motif	p-value	% of targets	TF
1.		1e-230	32.37%	Fra1
2.		1e-38	30.85%	ERG (ETS)
3.		1e-37	11.18%	RUNX
4.		1e-35	15.04%	TEAD
5.		1e-32	13.87%	Gata4
6.		1e-24	18.36%	KLF4

Appendix Figure S5

Appendix Figure S5. *LINC00313* does not cause global alterations on chromatin accessibility.

A-B) Tag distributions across target regions such as Merged Regions (= all peak regions; +/- 5 kb), transcription start sites (TSS; +/- 5 kb) or gene bodies (with 2 kb flanking regions) are presented as average plots (A) and as heatmaps (B). C) Piecharts displaying the location of ATAC-seq peaks relative to genomic annotations. Several peaks are assigned to more than one feature. D) HOMER-based analysis of transcription factor binding motifs in chromatin regions with increased or decreased peak signal after LINC00313 over-expression. The top 6 most significant consensus motifs, together with the percentage of target regions that are assigned to, are depicted.

Data information: In panel B for the heatmaps the data is clustered using k-means algorithm (default=5 clusters indicated by C1-C5) and sorted by decreasing average values inside each cluster. The graphics of panels A and B were generated with the Seqplots Bioconductor R package using the commands plotAverage() and plotHeatmap(). In panel D, HOMER analysis was performed on the 200 bp sequence centered around the midpoint of the differential región (+100 bp, -100 bp).

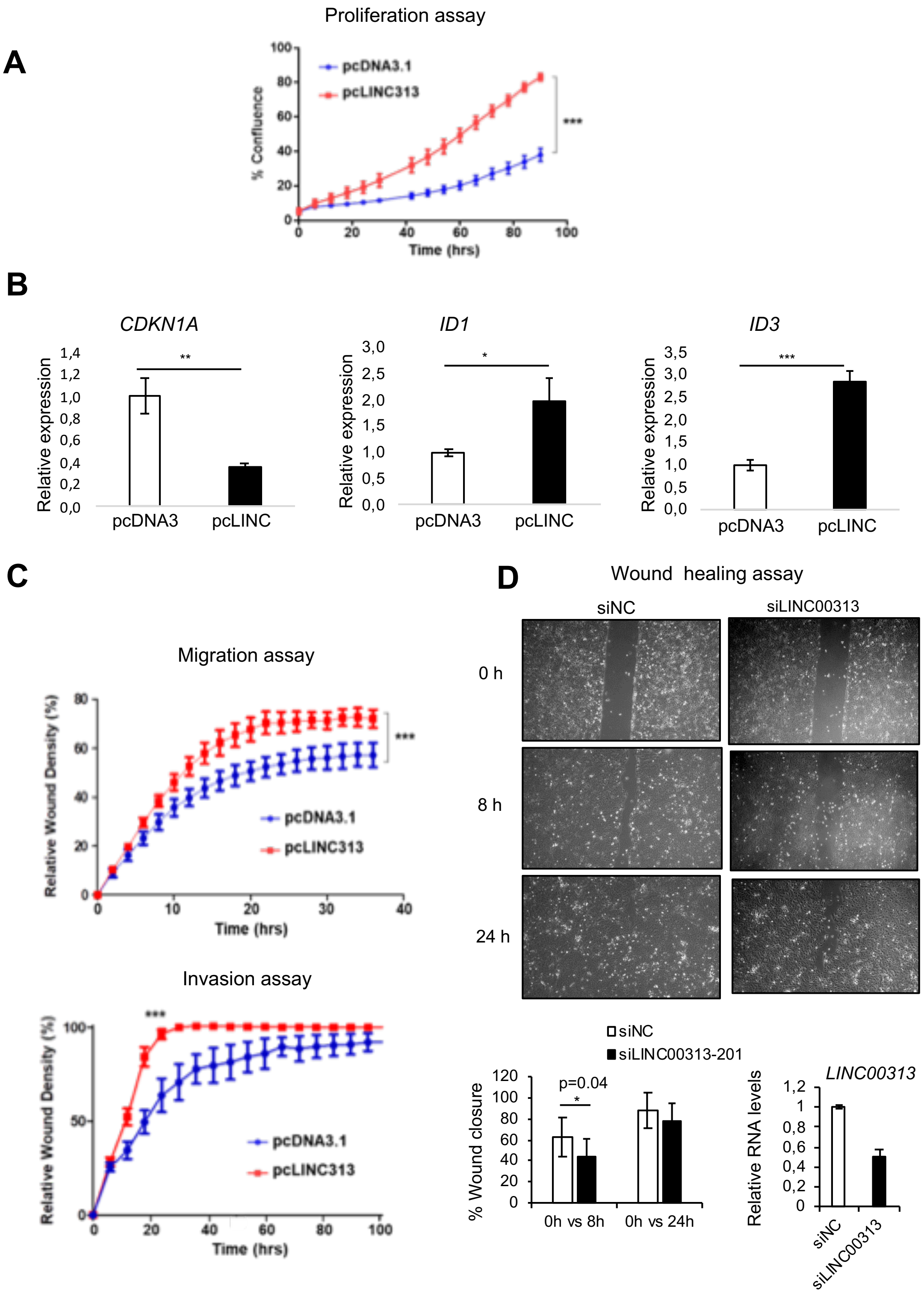

Appendix Figure S6

Appendix Figure S6. *LINC00313* affects HuCCT1 cell proliferation, migration and invasion.

A) Cell proliferation assay in control or LINC00313 stably over-expressing HuCCT1 cells, using Incucyte. B) Real-time qPCR analysis of *CDKN1A*, *ID1* and *ID3* mRNA in control or LINC00313 stably over-expressing HuCCT1 cells. C) Cell migration and invasion assays in control or LINC00313 stably over-expressing HuCCT1 cells, using Incucyte. D) Wound healing assay in HuCCT1 cells, transiently transfected with an individual siRNA targeting LINC00313 or a non-targeting siRNA (siNC) and in pcDNA3 and pcLINC00313 over-expressing cells. Quantification of migration is shown as percentage of wound closure after 8h and 24h since the scratch was performed. Real-time qPCR analysis of LINC00313 to quantify the efficiency of LINC00313 silencing is also shown.

Data information: In panel A, B, C and D data are presented as mean ± SD (n=3 biological experiments). *P≤0.05, **P≤0.01, ***P≤0.001 (Student's t-test).

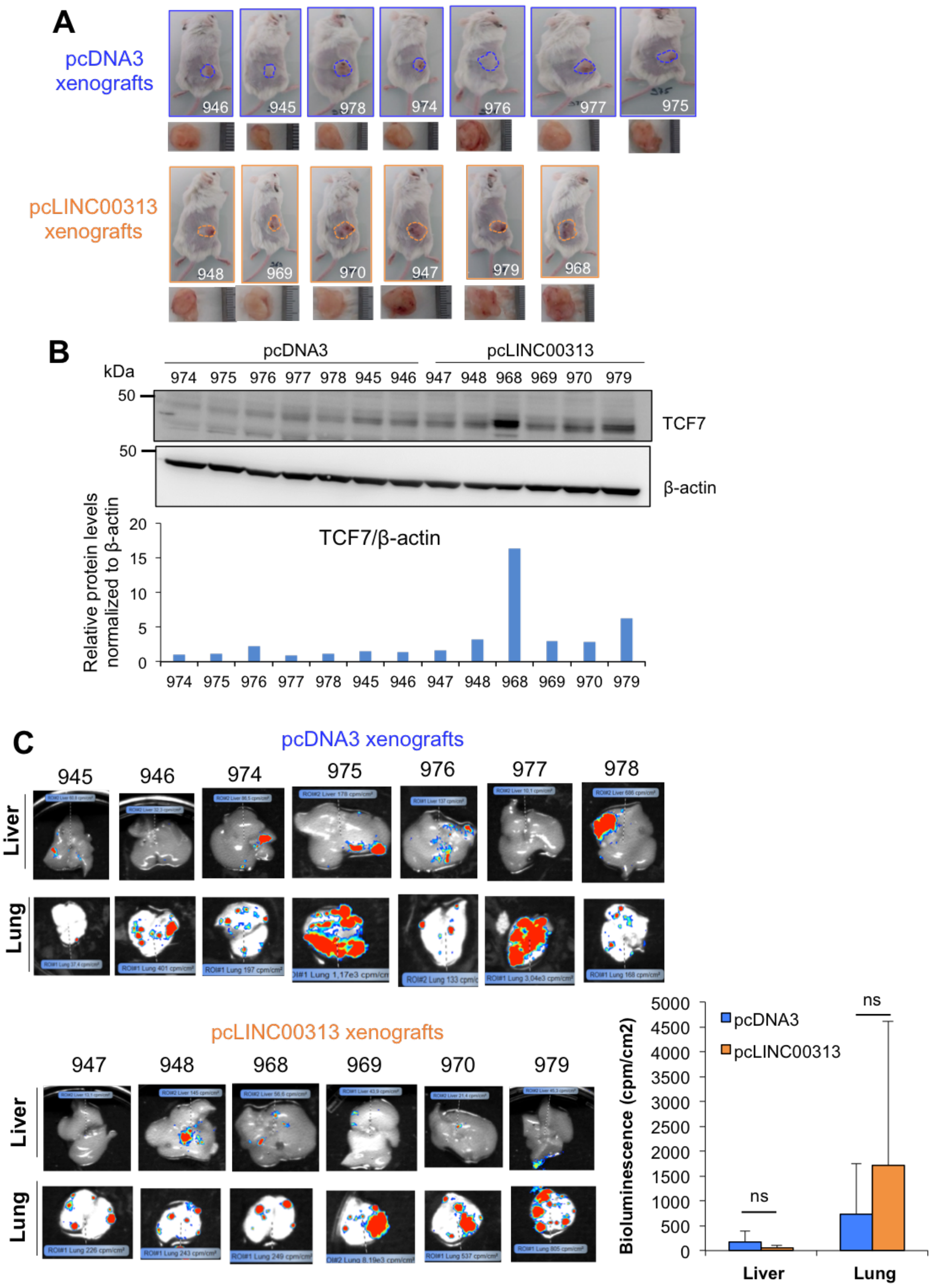

Appendix Figure S7

Appendix Figure S7. Impact of LINC00313 on tumor growth and metastasis in a xenograft CCA mouse model.

A) Picture of the mice and the corresponding resected tumours, used for analyzing tumor growth and metastasis and gene expression profiling (pcDNA3, n=7 and pcLINC00313, n=6). B) Immunoblotting for detection of TCF7 and β-actin protein levels using total protein extracts from the resected tumours as described in panel A. Quantification of the intensity of the TCF7 protein levels, normalized to β-actin levels is also shown. C) Pictures showing the presence of metastases in resected livers and lungs of the same mice. Bioluminescent signal expressed as cpm/cm^2, derived from HuCCT1 cells expressing luciferase, indicates the sites of metastases. Quantification of bioluminescence signal is also shown in the graph.

Data information: In panel B immunoblotting was performed once and quantified using ImageJ. In panel C data are presented as mean ± SD (pcDNA3, n=7 and pcLINC00313, n=6). n.s.: not significant (Student's t-test).

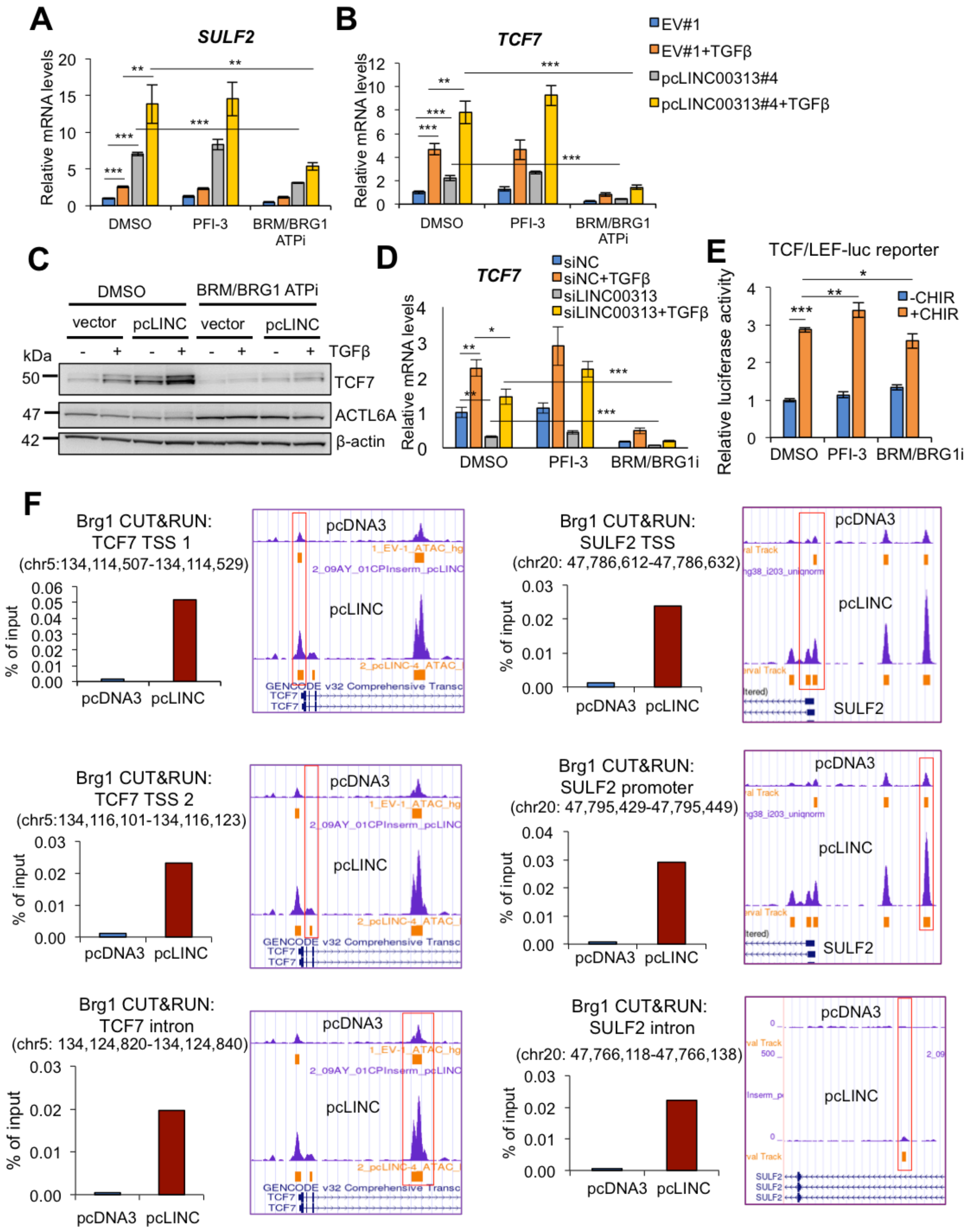

Appendix Figure S8

Appendix Figure S8. The SWI/SNF complex catalytic subunit BRG1 regulates specific LINC00313-target genes.

A) qPCR analysis of *SULF2* mRNA in pcDNA3 or *LINC00313* over-expressing HuCCT1 treated with PFI-3 or BRM/BRG ATPi or DMSO and with or without TGFβ1. B) qPCR analysis of *TCF7* mRNA in the same conditions as in panel A. Data are presented as mean ± SD (n=3 biological replicates). C) Immunoblotting for TCF7, ACTL6A and β-actin protein levels in the same conditions as in panel A.

D) qPCR analysis of *TCF7* mRNA levels in HuCCT1 cells transiently transfected with siRNAs targeting LINC00313 or a non-targeting siRNA (siNC) and treated with PFI-3 or BRM/BRG ATPi or DMSO and in the presence or not of TGFβ1. Data are presented as mean ± SD (n=3 biological replicates).

E) TCF/LEF luciferase reporter assay in HuCCT1 cells treated with DMSO, PFI-3 or BRM/BRG ATPi and in the presence or not of CHIR99021. Data are presented as mean ± SD (n=4 biological replicates).

F) CUT&RUN assays to evaluate Brg1 binding to the specified genomic regions in pcDNA3 or *LINC00313* over-expressing HuCCT1 cells. Experiment was performed in simplicate.

Data information: In panels A, B, D, E data are presented as mean ± SD (n=3 biological experiments). *P≤0.05, **P≤0.01, ***P≤0.001 (Student's t-test). In panel F (n=1).

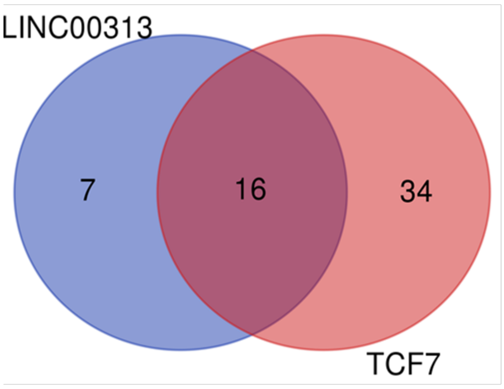

List of 16 common miRNAs
miR-150/5127
miR-24/24ab/24-3p
miR-205/205ab
miR-218/218a
miR-503
miR-7/7ab
miR-122/122a/1352
miR-15abc/16/16abc/195/322/424/497/1907
miR-204/204b/211
miR-214/761/3619-5p
miR-199ab-5p
miR-103a/107/107ab
miR-143/1721/4770
miR-31
miR-138/138ab
miR-93/93a/105/106a/291a-3p/294/295/302abcde/372/373/428/519a/520be/520acd-3p/1378/1420ac

Appendix Figure S9. Prediction of miRNAs that commonly target LINC00313 and TCF7.

Appendix Table S1. List of siRNAs

RNAi reagent	Sequence	Product ID
siNC	UGGUUUACAUGUCGACUAA<br>UGGUUUACAUGUUGUGUGA<br>UGGUUUACAUGUUUUCUGA<br>UGGUUUACAUGUUUUCCUA	ON-TARGETplus Non-targeting Pool, D-001810-10-05, Dharmacon
siLINC00313	Sense:<br>CGAGAAGCCCGAUGGGAAAUU<br>Antisense:<br>UUUCCCAUCGGGCUUCUCGUU	Custom-made individual siRNA, CTM-484556, Dharmacon
siLINC00313#2	Sense:<br>CGGGCUUCCUGGAUUGCAUUU<br>Antisense:<br>AUGCAAUCCAGGAAGCCCGUU	Custom-made individual siRNA, CTM-632606, Dharmacon
siSMAD2	GAAUUGAGCCACAGAGUAA<br>GGUUUACUCUCCAAUGUUA<br>UCAUAAAGCUUCACCAAUC<br>ACUAGAAUGUGCACCAUAA	ON-TARGETplus Human SMAD2 siRNA SMARTpool, L-003561-00-0005, Dharmacon
siSMAD3	CAACAGGAAUGCAGCAGUG<br>GAGUUCGCCUUCAAUAUGA<br>GGACGCAGGUUCUCCAAAC<br>UUAGAGACAUCAAGUAUGG	ON-TARGETplus Human SMAD3 siRNA SMARTpool, L-0020067-00-005, Dharmacon
siSMAD4	GCAAUUGAAAGUUUGGUAA<br>CCCACAACCUUUAGACUGA<br>GAAUCCAUAUCACUACGAA<br>GUACAGAGUUACUACUUAG	ON-TARGETplus Human SMAD4 siRNA SMARTpool, L-003902-00-0005, Dharmacon
siACTL6A	GAACGGAGGUUUAGCUCAU<br>CCUACUACAUAGAUACUAA<br>GUAAAGGGGUUAUCAGGAA<br>UGGGAUAGUUUCCAAGCUA	ON-TARGETplus Human ACTL6A siRNA SMARTpool, L-008243-00-0005, Dharmacon
siMAPK11	GCCCUGAGGUUCUGGCAAA<br>CGACGAGCACGUUCAAUUC<br>CCAUAGACCUCCUUGGAAG<br>GCGCCGACCUGAACAACAU	ON-TARGETplus Human MAPK11 siRNA SMARTpool, L-003972-00-0005, Dharmacon
siMAPK12	GAAGCGUGUUACUUACAAA<br>GCGCUAAGGUGGCCAUCAA<br>GCAAGACGCUGUUCAAGGG<br>GGAGACGCCUCUGUGAAGA	ON-TARGETplus Human MAPK12 siRNA SMARTpool, L-003590-00-0005, Dharmacon
siMAPK13	GCUCAAAGGCCUUAAGUAC<br>GGAGUGGCAUGAAGCUGUA<br>GGAUUUCACUCAGCUGUUC<br>GCCGUUUGAUGAUUCCUUA	ON-TARGETplus Human MAPK13 siRNA SMARTpool, L-003591-00-0005, Dharmacon
siMAPK14	GGAAUUCAAUGAUGUGUAU	ON-TARGETplus Human MAPK14

	UCUCCGAGGUCUAAAGUAU<br>GUAAUCUAGCUGUGAAUGA<br>GUCCAUCAUUCAUGCGAAA	siRNA SMARTpool, L-003512-00-0005, Dharmacon

Appendix Table S2. List of DNA oligonucleotides (F: forward; R: reverse)

RT-qPCR primers	Sequence
TBP	F, GAGCTGTGATGTGAAGTTTCC<br>R, TCTGGGTTTGATCATTCTGTAG
GAPDH	F, GACCACAGTCCATGCCATCA<br>R, TCCACCACCCTGTTGCTGTA
LINC00313	F, GCGGGAAACCTCGATGAACA<br>R, ACATTCTTTCCCATCGGGCT
LINC00313 exon 1	F, CGGGCTTCCTGGATTGCATA<br>R, GCTGCTGACGGTTGTTTTGA
LINC00313 exon 3	F, AACACCCCGGATCGAGAAG<br>R, GAGTGGAAACTGTCACGCAA
LINC00313 exon 4	F, GGAAATGACCGCAGAAGGGAG<br>R, TAGCAGCGCCAAGTTCTTTCT
LINC00313-205	F, CTTGGAAGCCGAGCATCAGT<br>R, CCTCCTGCCGAATAACGACT
SMAD2	F, AGACACCAGTTTTGCCTCCA<br>R, GAGGTGGCGTTTCTGGAATA

SMAD3	F, CTAGGGCTGCTCTCCAATGT<br>R, AAGACCTCCCCTCCGATGTA
SMAD4	F, GCTGCTGGAATTGGTGTTGATG<br>R, AGGTGTTTCTTTGATGCTCTGTCT
TCF7	F, GTACAAAGAGACCGTCTACTCC<br>R, GGCTGTTGAAATGTTCGTAGAG
WNT5A	F, CGCCCAGGTTGTAATTGAAG<br>R, GCATGTGGTCCTGATACAAGT
AXIN2	F, ATTCGGCCACTGTTCAGACG<br>R, GACAACCAACTCACTGGCCTG
SULF2	F, CTGTGGGAAGGCTGGGAAGG<br>R, TGAGAGTGCGTGCTTGCTTTC
ACTL6A	F, ATGTGTGATATTGACATCAGACCAG<br>R, CGCCAATCCATGAGCTAAACC
MALAT1	F, TGTGTGCCAATGTTTCGTTT<br>R, AGGAGAAAGTGCCATGGTTG
RNU48	F, AGTGATGATGACCCCAGGTAACTCTG<br>R, TCAGAGCGCTGCGGTGATG
MAPK11	F, AAGCACGAGAACGTCATCGG<br>R, TCACCAAGTACACTTCGCTGA
MAPK12	F, GGGCTGCTGGACGTATTCAC<br>R, TGCCCATGAACGGCATCAC
MAPK13	F, TACATCCACTCTGCTGGGGT<br>R, GTCACCACGTAGCCAGTCAT
MAPK14	F, TCAGTCCATCATTCATGCGAAA<br>R, AACGTCCAACAGACCAATCAC
**CUT&RUN primers**	**Sequence**
TCF7 TSS 1	F, TGATCATTCCCAGGCTTGTCC

	R, CATAGCCTTAAAGGGCTCGCT
TCF7 TSS 2	F, GCAAGAGACTTCTGCCTGGAA<br><br>R, CCGGGTGTGTATGGGAGAAAA
TCF7 intron	F, ACTTTGGGGGTTTACACGGT<br><br>R, ACCCAAACCAGCTCAGACAT
SULF2 TSS	F, GCCCGGGAGTAGCATAGAAA<br><br>R, TAGACAGCACCCCCTTGAGT
SULF2 promoter	F, GAGCAGTGTCTTCTGTCCGT<br><br>R, GGGCAGCGATTCCATCAAGT
SULF2 intron	F, TGGTCCCAAACCTCACAAGC<br><br>R, ACAGATGTGCGTGTGTCATC
**smiFISH probes**	**Sequence**
LINC00313 probe1	AAG AGA TGC AGG CCC ATC GTG TGC ACT TAC ACT CGG ACC TCG TCG ACA TGC ATT
LINC00313 probe2	ACC TTT GCC CTG GCT ATG TAA GGA GAA GTT ACA CTC GGA CCT CGT CGA CAT GCA TT
LINC00313 probe3	CCT TTA TGC AAT CCA GGA AGC CCG CGT TAC ACT CGG ACC TCG TCG ACA TGC ATT
LINC00313 probe4	CGC CAT TGC CTT AGC AGC GCC AAG TTT TAC ACT CGG ACC TCG TCG ACA TGC ATT
LINC00313 probe5	TTC TGG GGC AAT CGC CGC TGT TGG TTT TAC ACT CGG ACC TCG TCG ACA TGC ATT
LINC00313 probe6	CGG GCA TGA CGT CCT TCC CAG ACA TTT TAC ACT CGG ACC TCG TCG ACA TGC ATT
LINC00313 probe7	CAT CGG GCT TCT CGA TCC GGG GTG TTT TAC ACT CGG ACC TCG TCG ACA TGC ATT
LINC00313 probe9	TTC CAG CGC TTC CAC ACT TCA CTC GAT TAC ACT CGG ACC TCG TCG ACA TGC ATT
LINC00313 probe10	CGG TGT TTG TTC ATC GAG GTT TCC CGC TGT TAC ACT CGG ACC TCG TCG ACA TGC ATT
LINC00313 probe11	GAC GGT TGT TTT GAC TTG CTC AGA GTT GTC ATT TAC ACT CGG ACC TCG TCG ACA TGC ATT
LINC00313 probe12	CGG TGC ACT CCC TTC TGC GGT CAT TTT TAC ACT CGG ACC TCG TCG ACA TGC ATT
LINC00313 probe13	AGG TTC AGA GTG AGC GCA CTG TGA GTG GAA ATT ACA CTC GGA CCT CGT CGA CAT GCA TT
LINC00313 probe14	TCA CAA AGC AGG ATC TGC AGA AAC ACT CTT TAC ACT CGG ACC TCG TCG ACA TGC ATT
LINC00313 probe15	TTC CCC TCG TGT TTC CTA GCA GAT AGT GTT ACA CTC GGA CCT CGT CGA CAT GCA TT

Appendix Table S3. List of primary antibodies

Antibody	Dilution	Application	Source	Catalogue number
phospho-AKT (Ser473) (D9E)	1:1000	WB	Cell Signaling Technology	#4060
AKT (pan) (C67E7)	1:1000	WB	Cell Signaling Technology	#4691
phospho-SAPK/JNK (Thr183/Tyr185) (81E11)	1:1000	WB	Cell Signaling Technology	#4668
SAPK/JNK	1:1000	WB	Cell Signaling Technology	#9252
phospho-p44/42 MAPK (Erk1/2) (Thr202/Tyr204) (197G2)	1:1000	WB	Cell Signaling Technology	#4377
p44/42 MAPK (Erk1/2) (137F5)	1:1000	WB	Cell Signaling Technology	#4695
p38α MAPK	1:1000	WB	Cell Signaling Technology	#9218
p38β MAPK (C28C2)	1:1000	WB	Cell Signaling Technology	#2339
p38γ MAPK	1:1000	WB	Cell Signaling Technology	#2307
p38δ MAPK (10A8)	1:1000	WB	Cell Signaling Technology	#2308
phospho-p38 MAPK (Thr180/Tyr182) (D3F9)	1:1000	WB	Cell Signaling Technology	#4511
phospho-MAPKAPK-2 (Thr334) (27B7)	1:1000	WB	Cell Signaling Technology	#3007
SMAD2/3 (D7G7)	1:1000	WB	Cell Signaling Technology	#8685
SMAD4 (D3M6U)	1:1000	WB	Cell Signaling Technology	#38454
β-catenin (D10A8) XP	1:200 for IF, 1:1000 for WB	IF, WB	Cell Signaling Technology	#8480
TCF1/TCF7 (C63D9)	1:1000	WB	Cell Signaling Technology	#2203
HuR	1:1000	WB	ThermoFisher Scientific	1862775
HA tag	1:1000	WB	Abcam	ab9110
Lamin B1 (D9V6H)	1:1000	WB	Cell Signaling Technology	#13435
14-3-3	1:1000	WB	Abcam	ab125032

Brg1 (D1Q7F)	1:100 for RIP, 1:50 for CUT&RUN	RIP, CUT&RUN	Cell Signaling Technology	#49360
ACTL6A/BAF53A	1: 1000 for WB, 1:50 for RIP and IF	WB, RIP, IF	Cell Signaling Technology	#76682
β-tubulin	1:1000	WB	Abcam	ab15568
beta-actin	1:1000	WB	Abcam	ab8224
β-actin (D6A8)	1:1000	WB	Cell Signaling Technology	#8457
