# Supplementary figures and images for: TGFβ-induced long non-coding RNA LINC00313 activates Wnt signaling and promotes cholangiocarcinoma
Source: EMBO Rep. 2024 Feb 8;25(3):11. doi: 10.1038/s44319-024-00075-z (PMC10933437; doi:10.1038/s44319-024-00075-z)

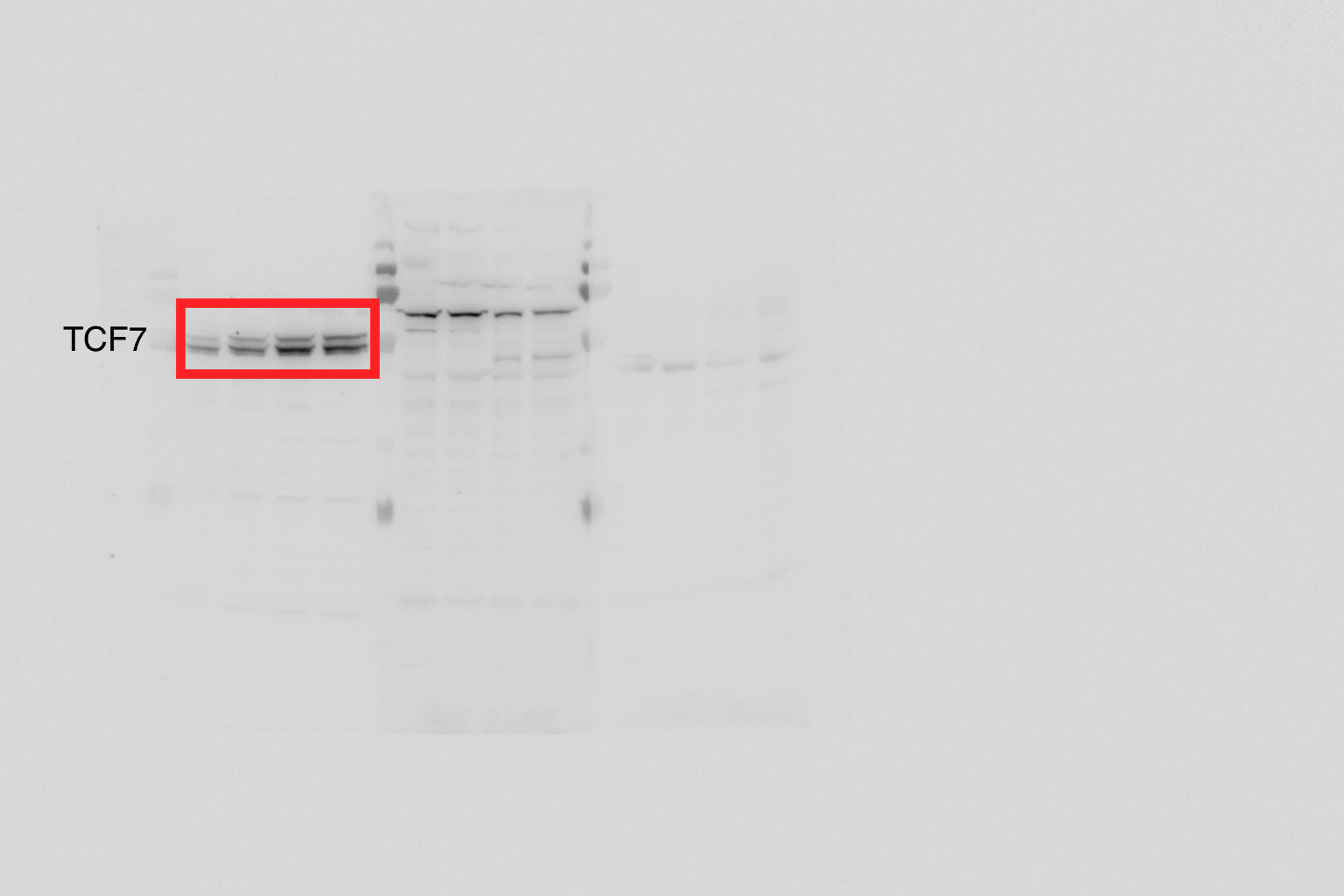

Supplement: Supplementary file 10 — Source Data Fig. 4 [file 44319_2024_75_MOESM10_ESM.zip › Figure 4/4F /WB/TCF7 5 sec pcLINC.tif]

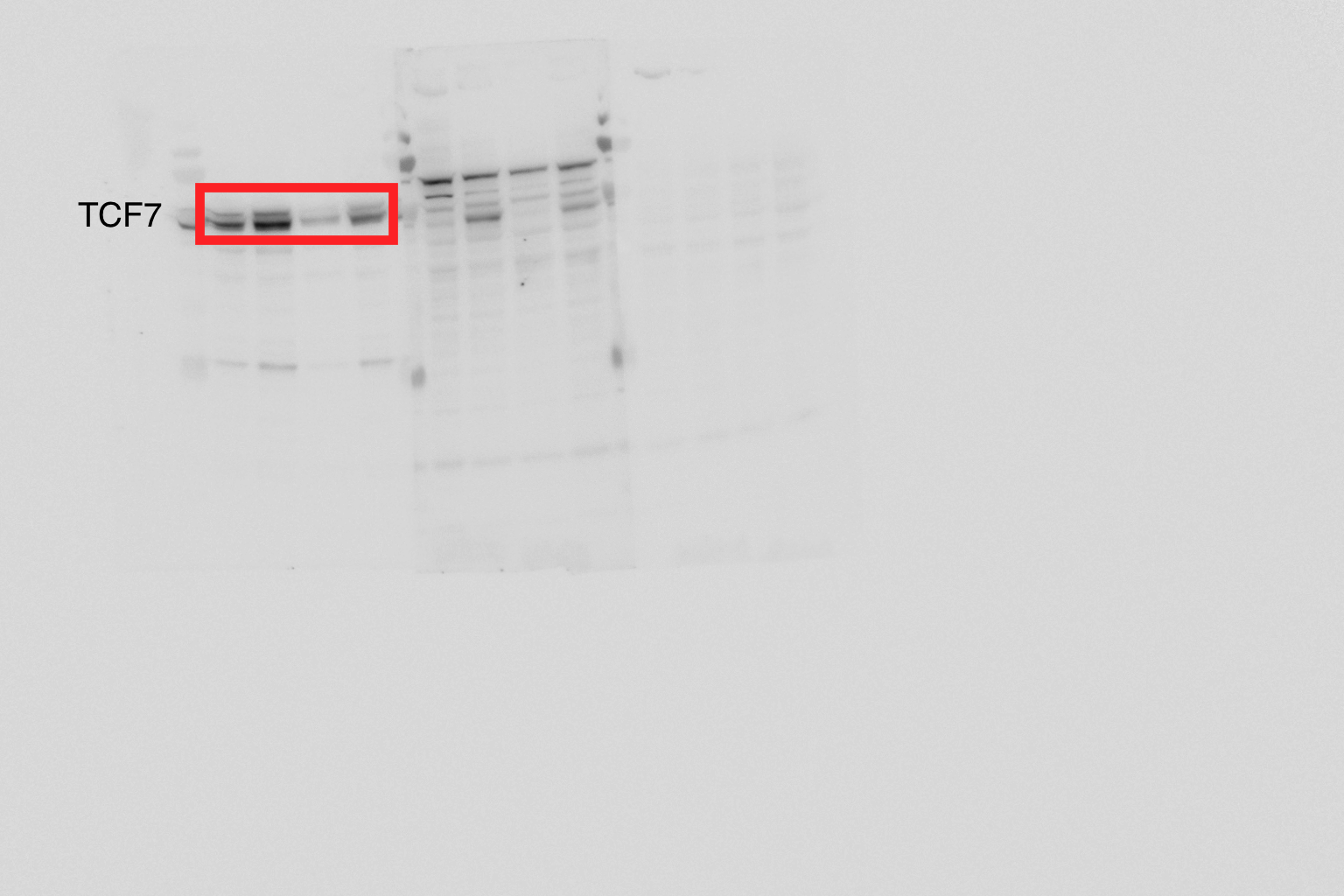

Supplement: Supplementary file 10 — Source Data Fig. 4 [file 44319_2024_75_MOESM10_ESM.zip › Figure 4/4G/WB/TCF7 5 sec siLINC.tif]

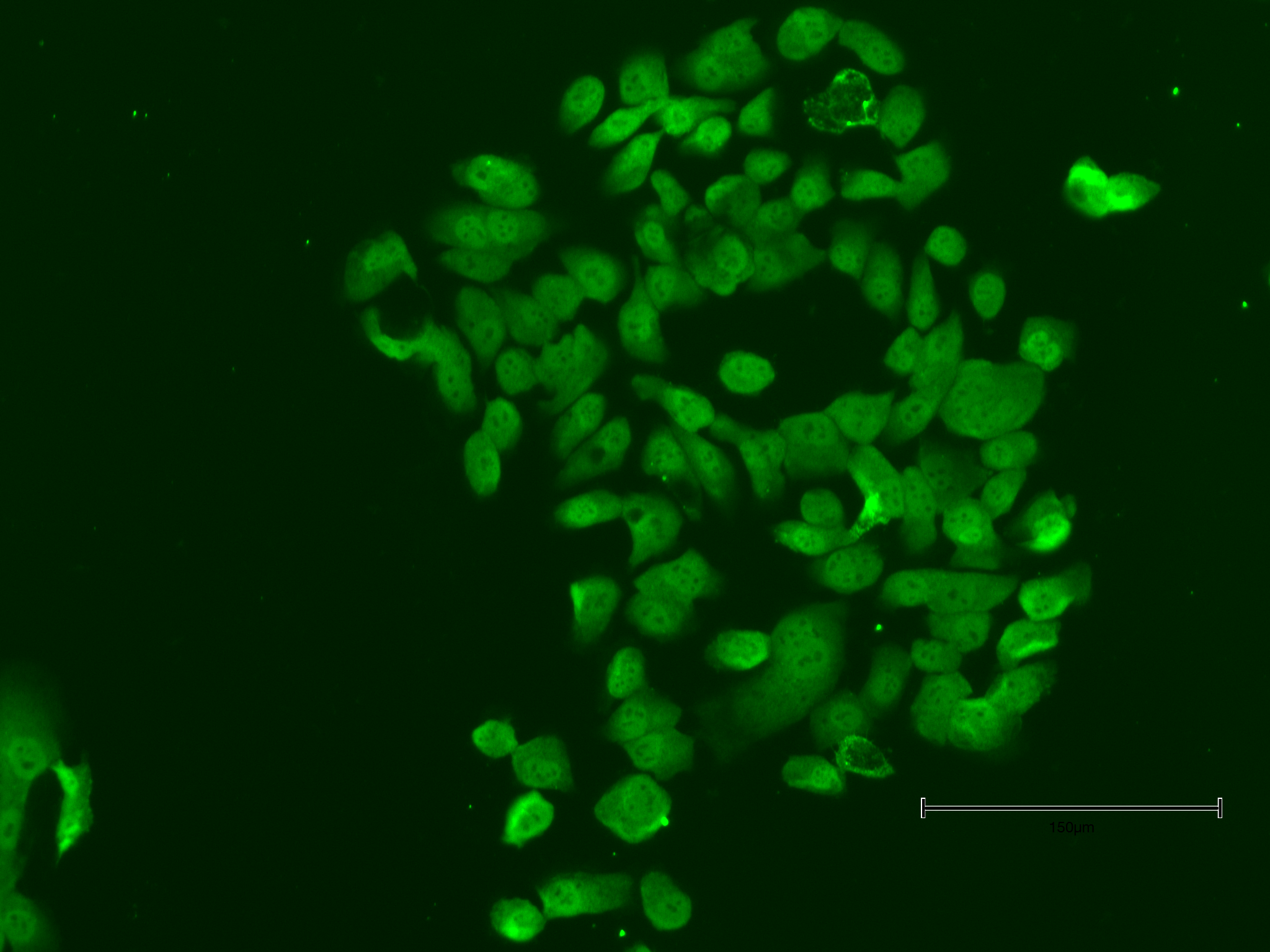

Supplement: Supplementary file 12 — Source Data Fig. 6 [file 44319_2024_75_MOESM12_ESM.zip › Figure 6/6G/pcDNA3.1_ctr.tif]

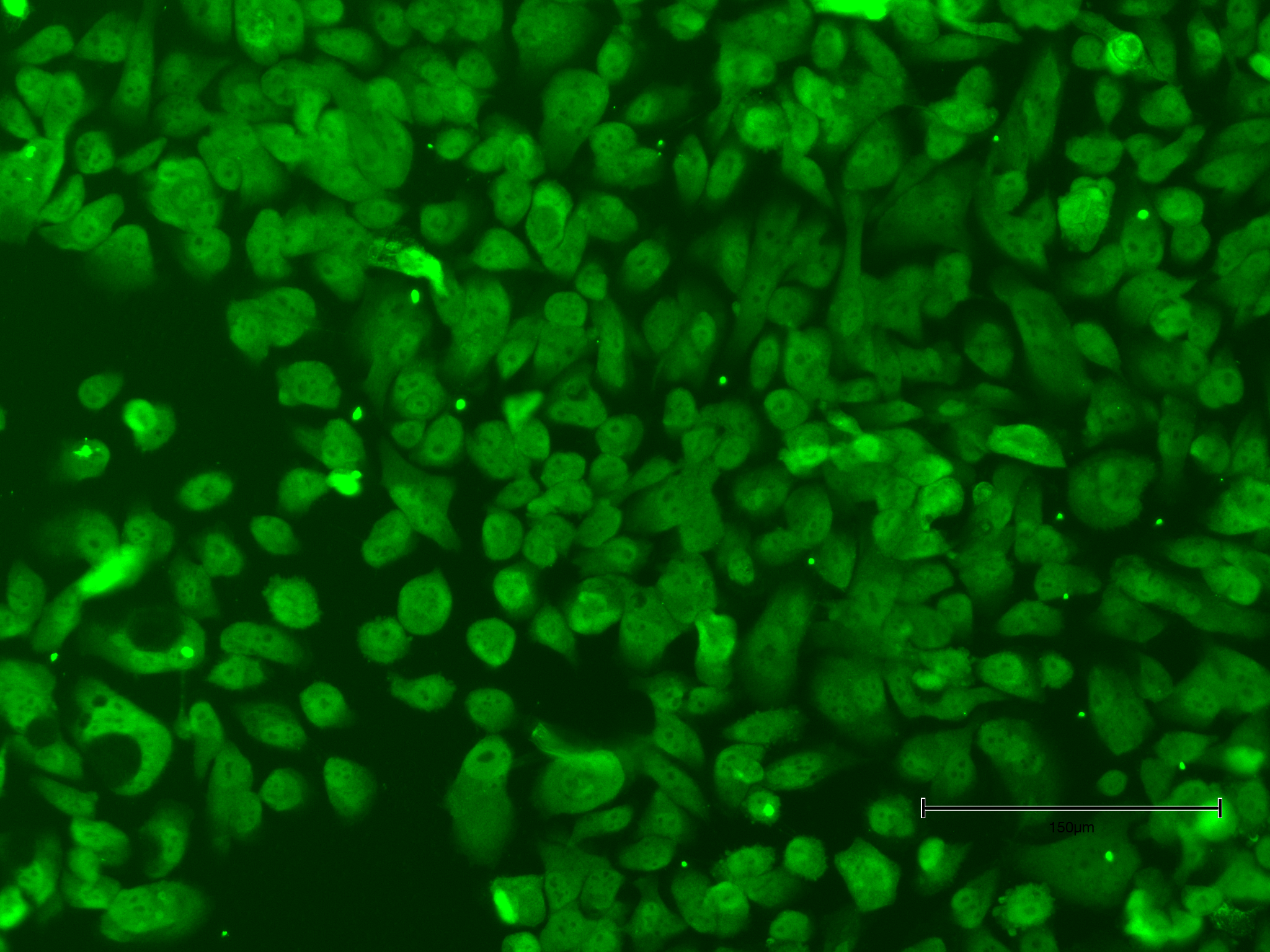

Supplement: Supplementary file 12 — Source Data Fig. 6 [file 44319_2024_75_MOESM12_ESM.zip › Figure 6/6G/pcDNA3.1_TGFb.tif]

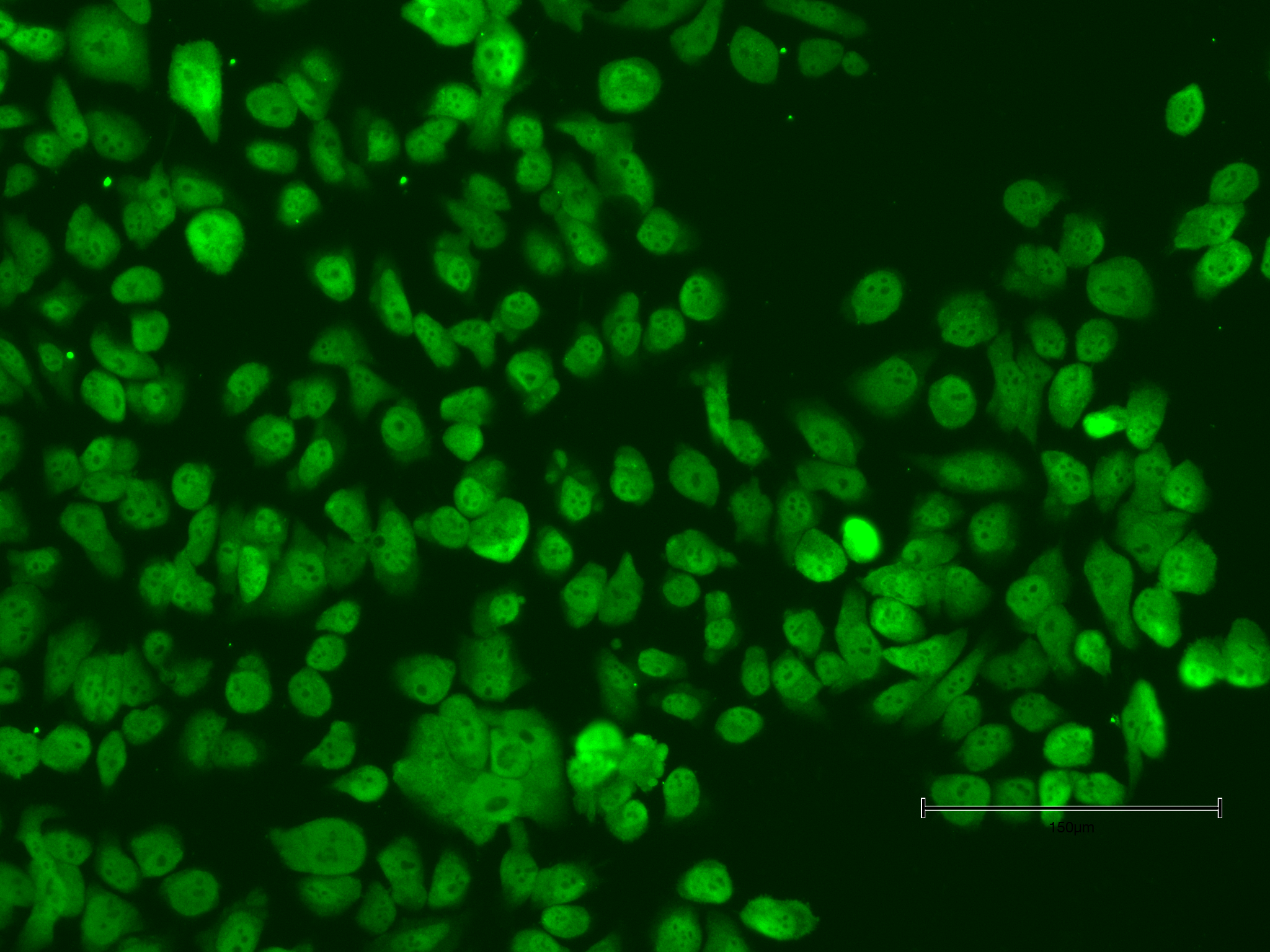

Supplement: Supplementary file 12 — Source Data Fig. 6 [file 44319_2024_75_MOESM12_ESM.zip › Figure 6/6G/pcLINC_ctr.tif]

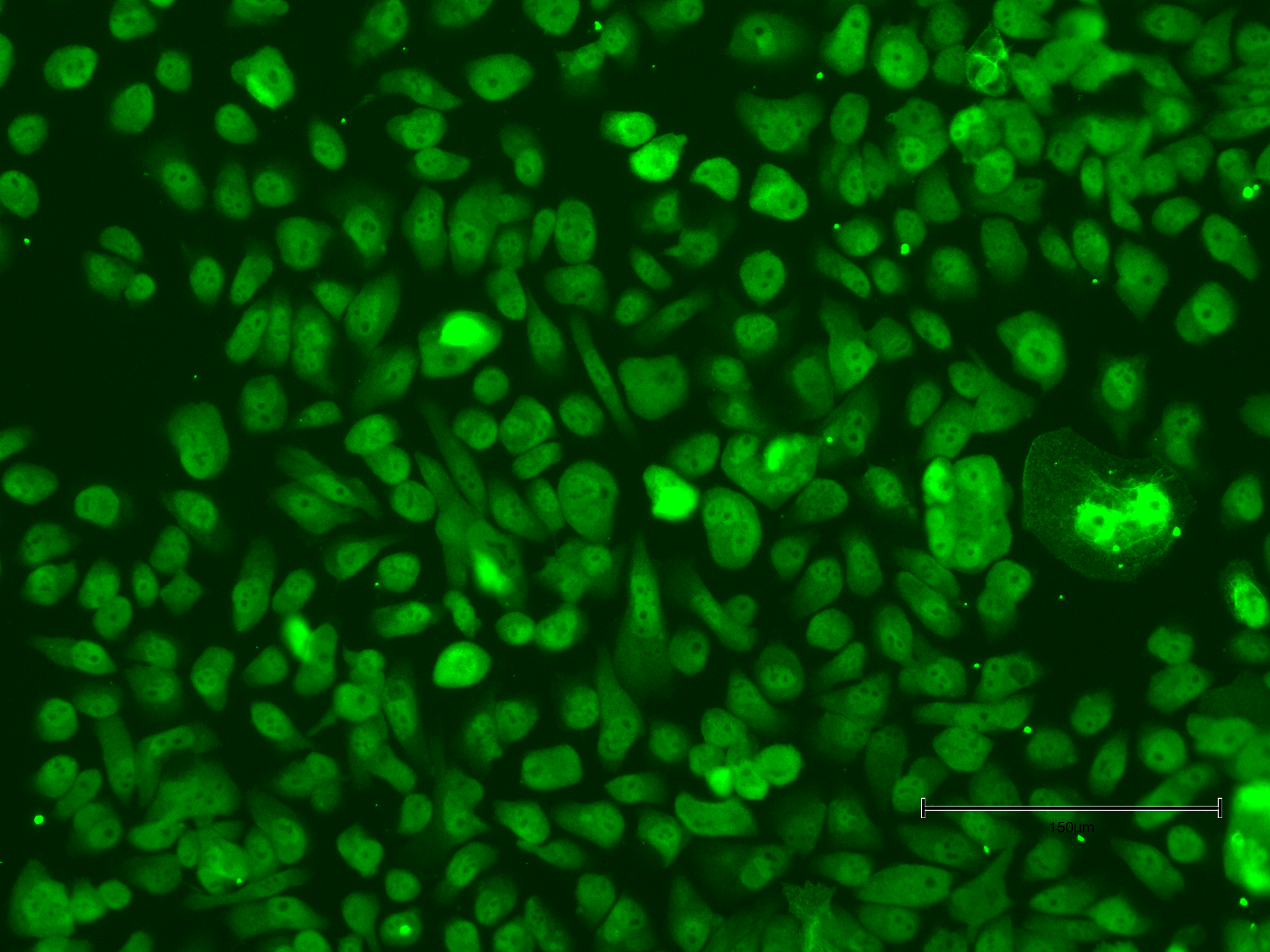

Supplement: Supplementary file 12 — Source Data Fig. 6 [file 44319_2024_75_MOESM12_ESM.zip › Figure 6/6G/pcLINC_TGFb.tif]

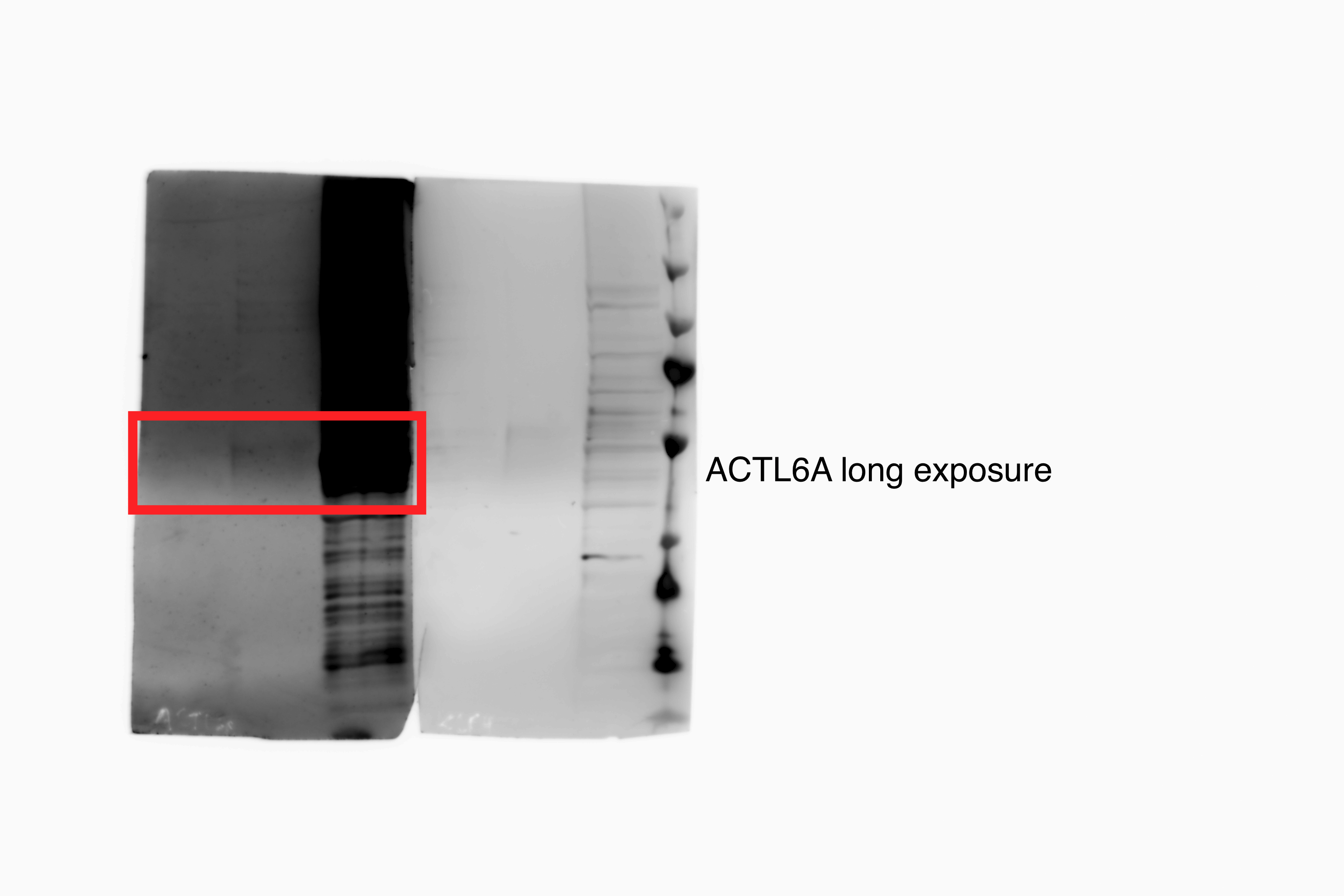

Supplement: Supplementary file 12 — Source Data Fig. 6 [file 44319_2024_75_MOESM12_ESM.zip › Figure 6/6H/RNA pull-down LIN C00313 ACTL6A HuCCT1 with TGFb/ACTL6A 30 sec.tif]

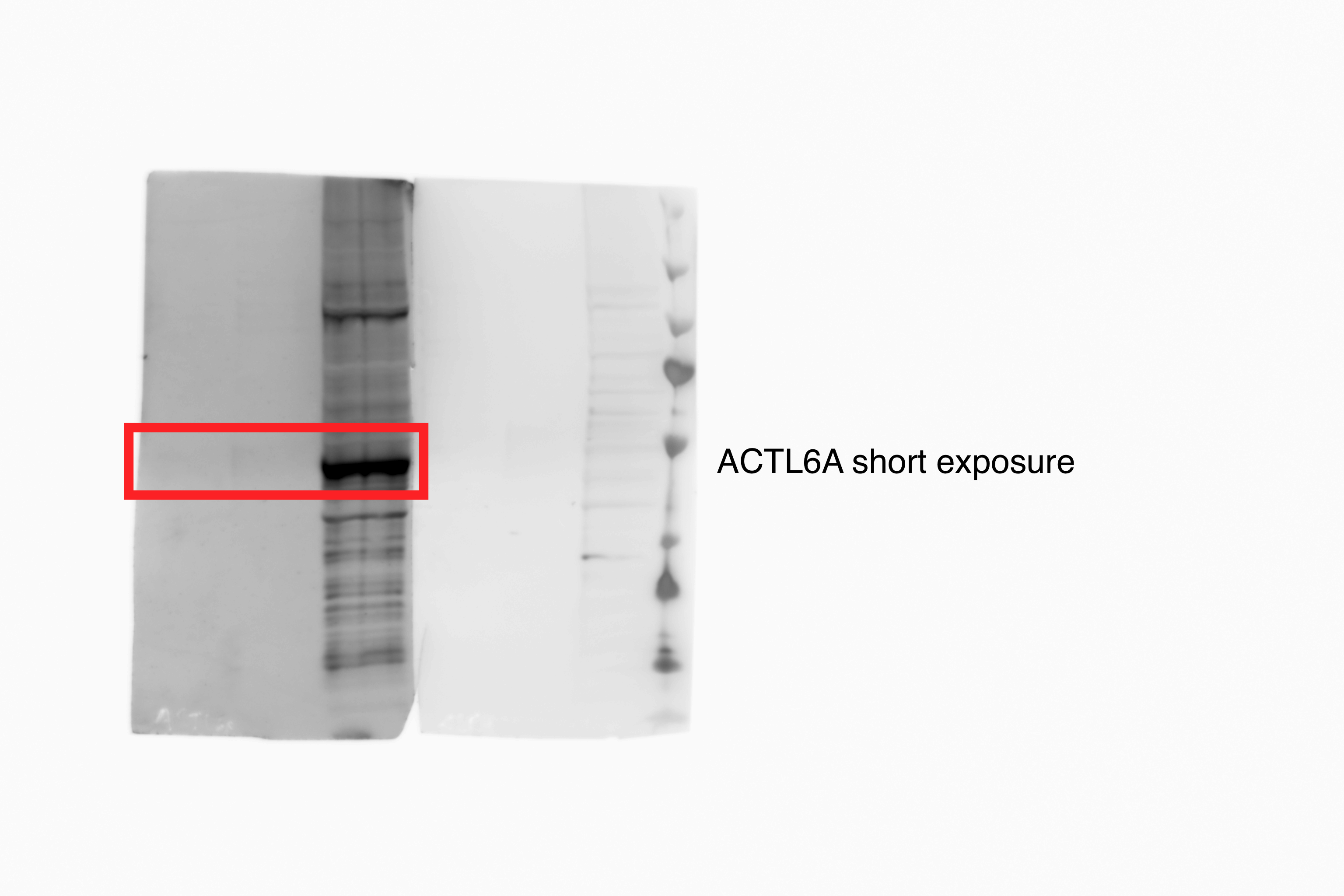

Supplement: Supplementary file 12 — Source Data Fig. 6 [file 44319_2024_75_MOESM12_ESM.zip › Figure 6/6H/RNA pull-down LIN C00313 ACTL6A HuCCT1 with TGFb/ACTL6A 5 sec.tif]

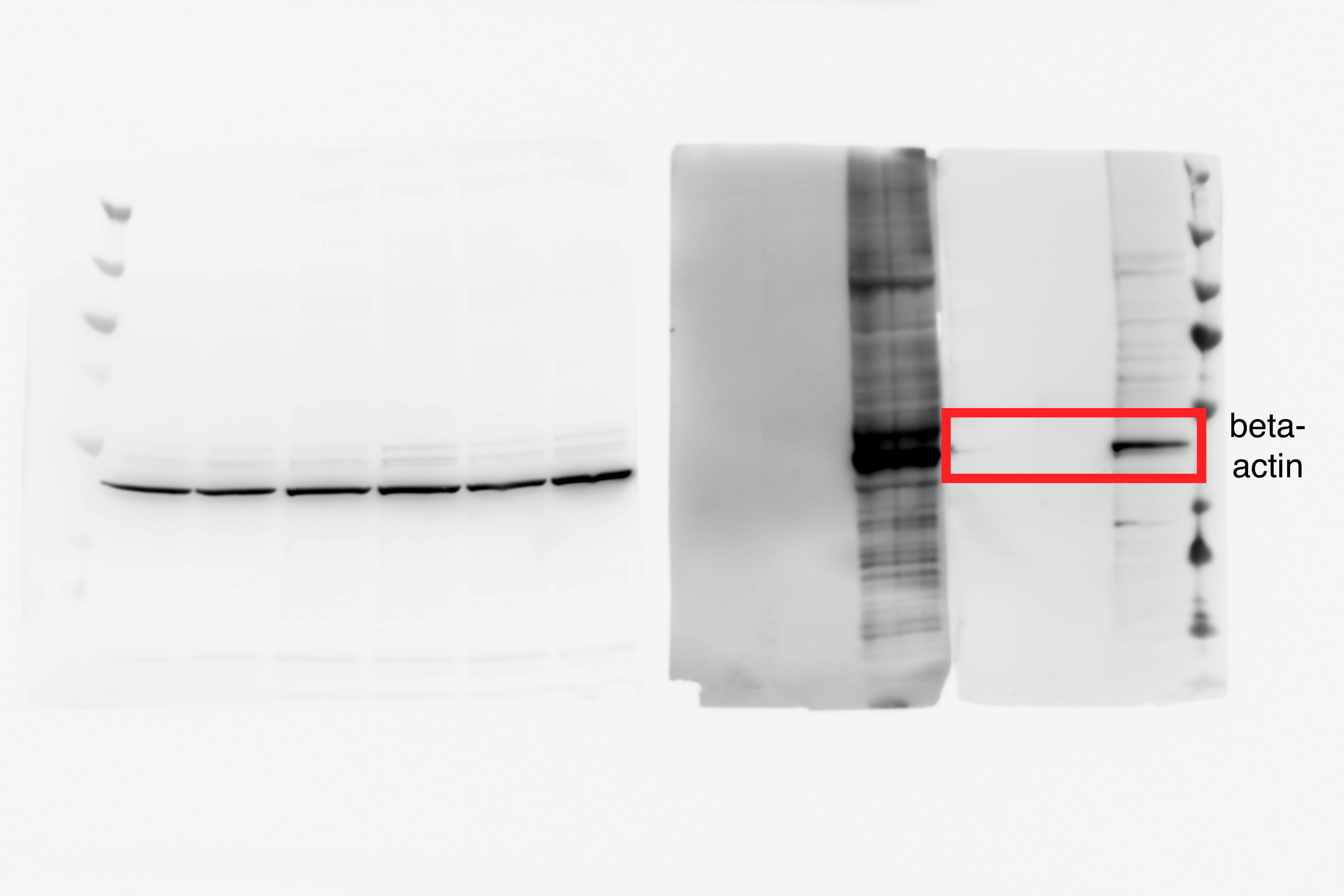

Supplement: Supplementary file 12 — Source Data Fig. 6 [file 44319_2024_75_MOESM12_ESM.zip › Figure 6/6H/RNA pull-down LIN C00313 ACTL6A HuCCT1 with TGFb/reblot b-actin 10 sec.tif]

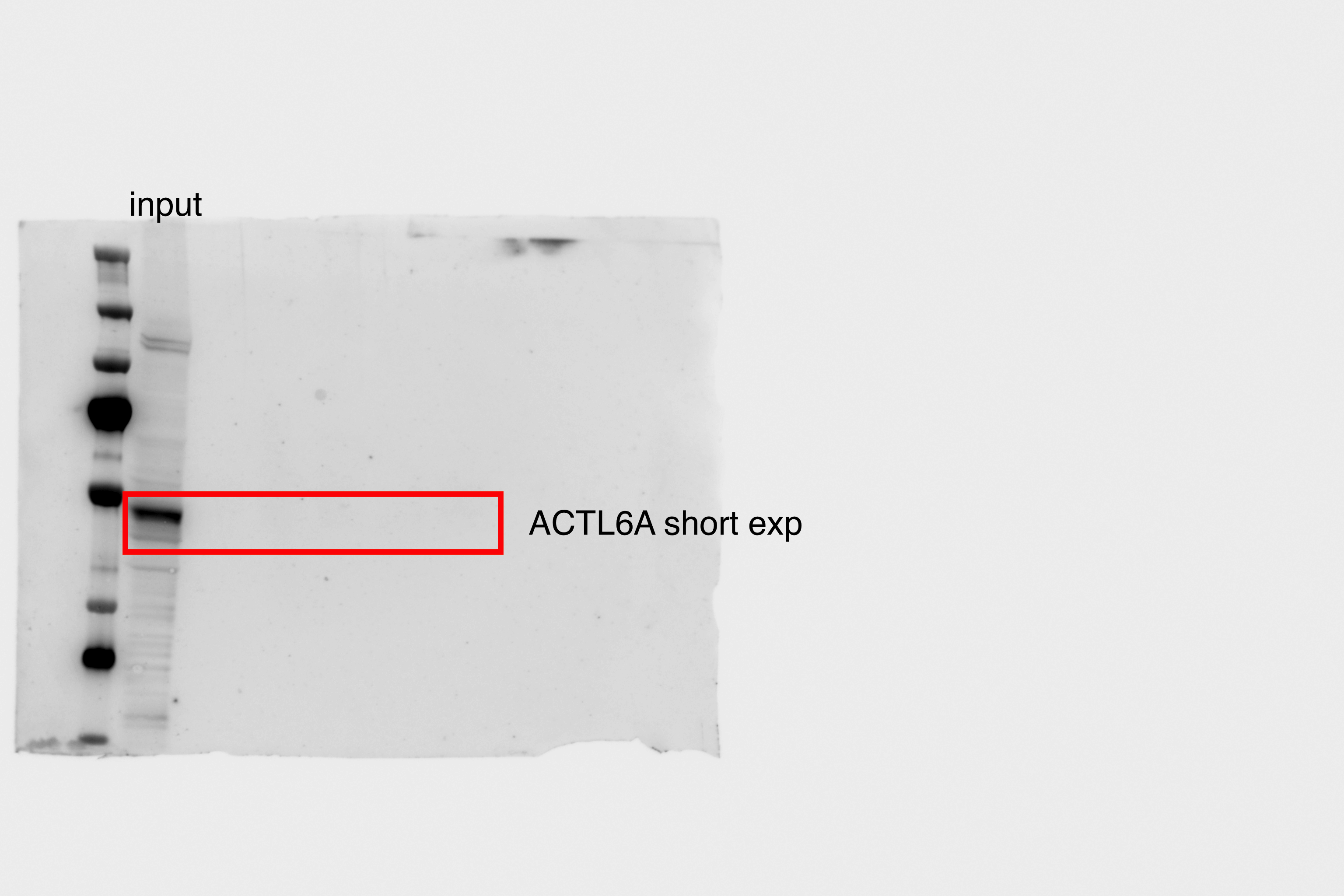

Supplement: Supplementary file 12 — Source Data Fig. 6 [file 44319_2024_75_MOESM12_ESM.zip › Figure 6/6H/RNA pull-down LINC00313 ACTL6A HuCCT1 no TGFb/ACTL6A 2 sec.tif]

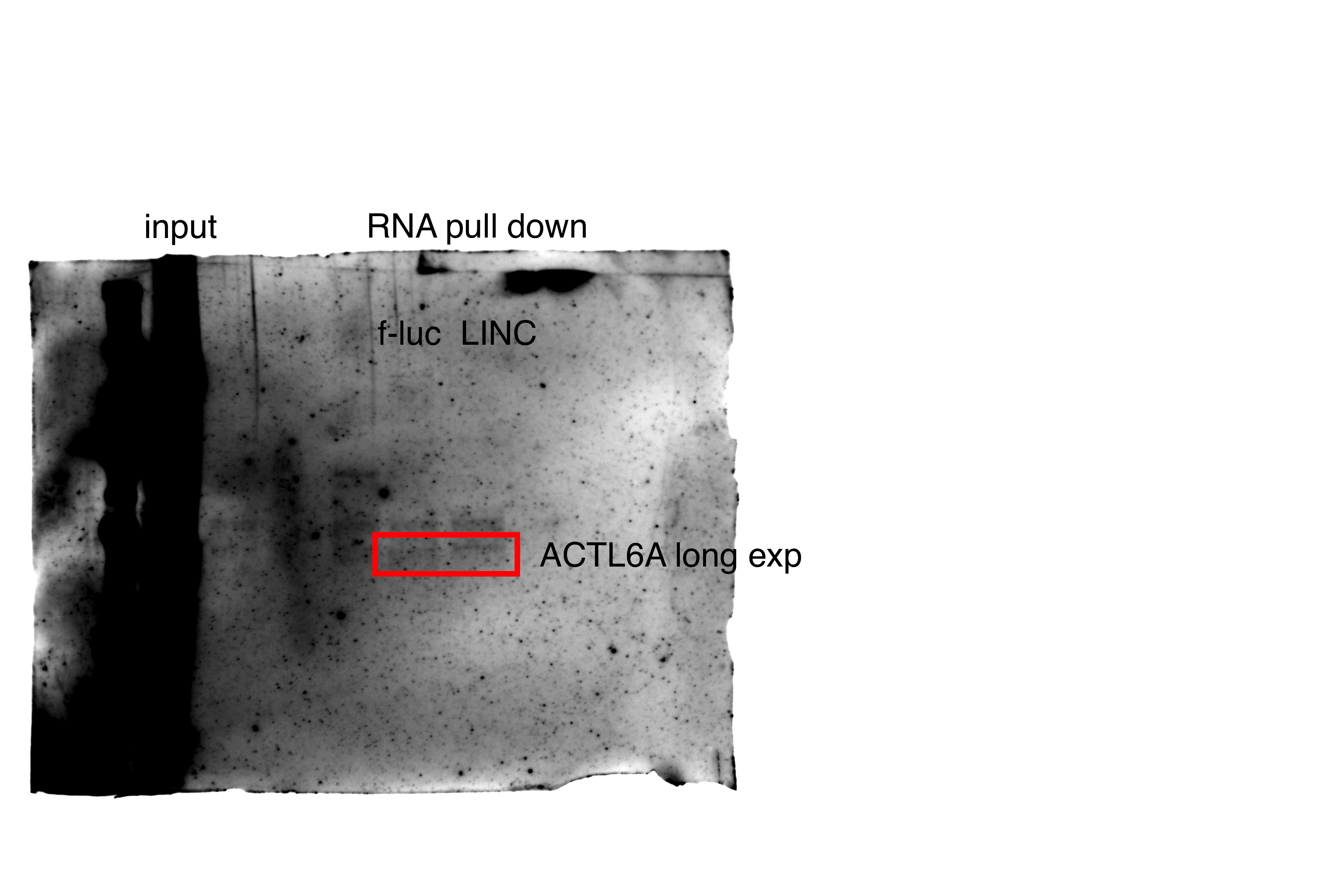

Supplement: Supplementary file 12 — Source Data Fig. 6 [file 44319_2024_75_MOESM12_ESM.zip › Figure 6/6H/RNA pull-down LINC00313 ACTL6A HuCCT1 no TGFb/ACTL6A 5 min.tif]

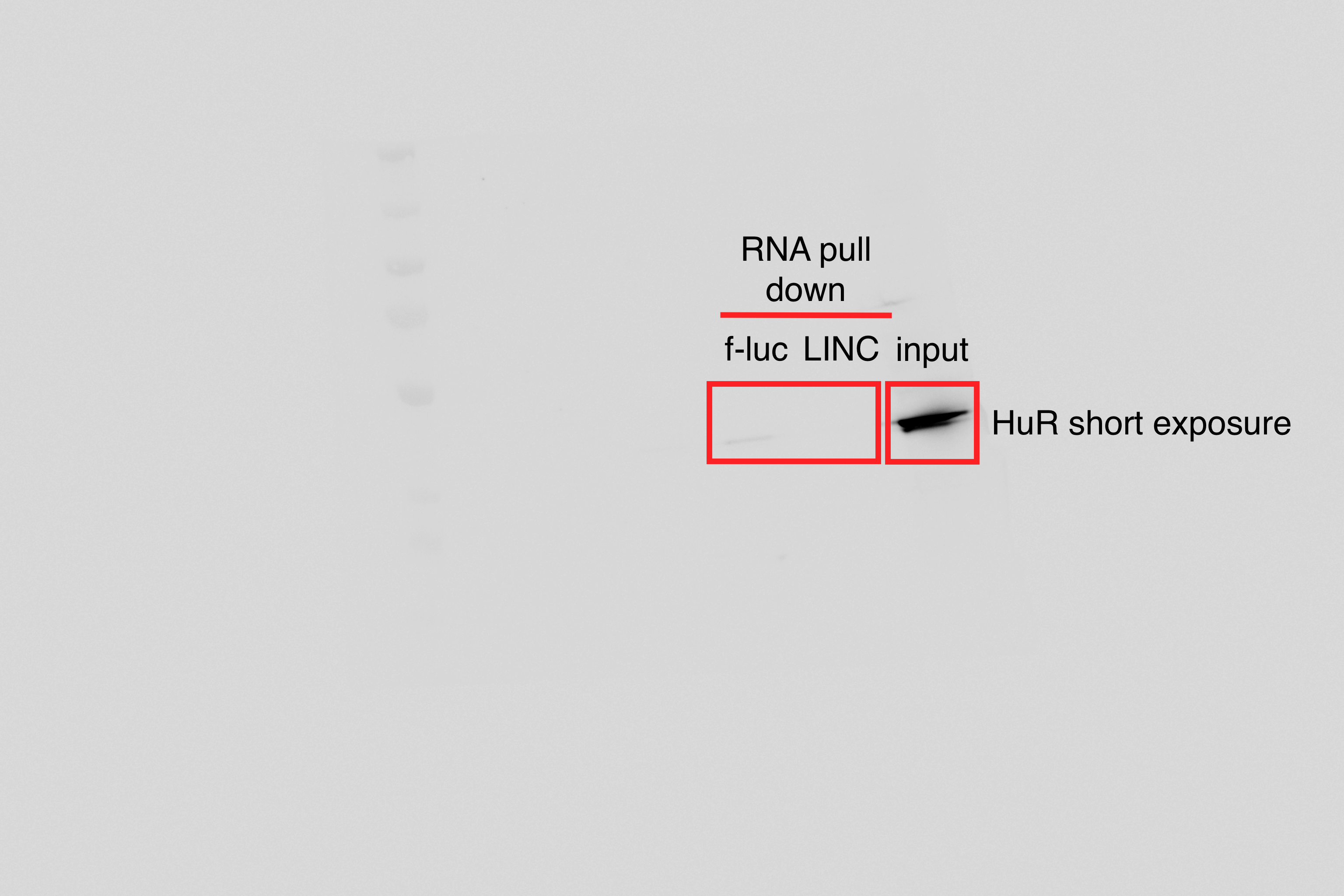

Supplement: Supplementary file 12 — Source Data Fig. 6 [file 44319_2024_75_MOESM12_ESM.zip › Figure 6/6H/RNA pull-down LINC00313 ACTL6A HuCCT1 no TGFb/HuR 1 min.tif]

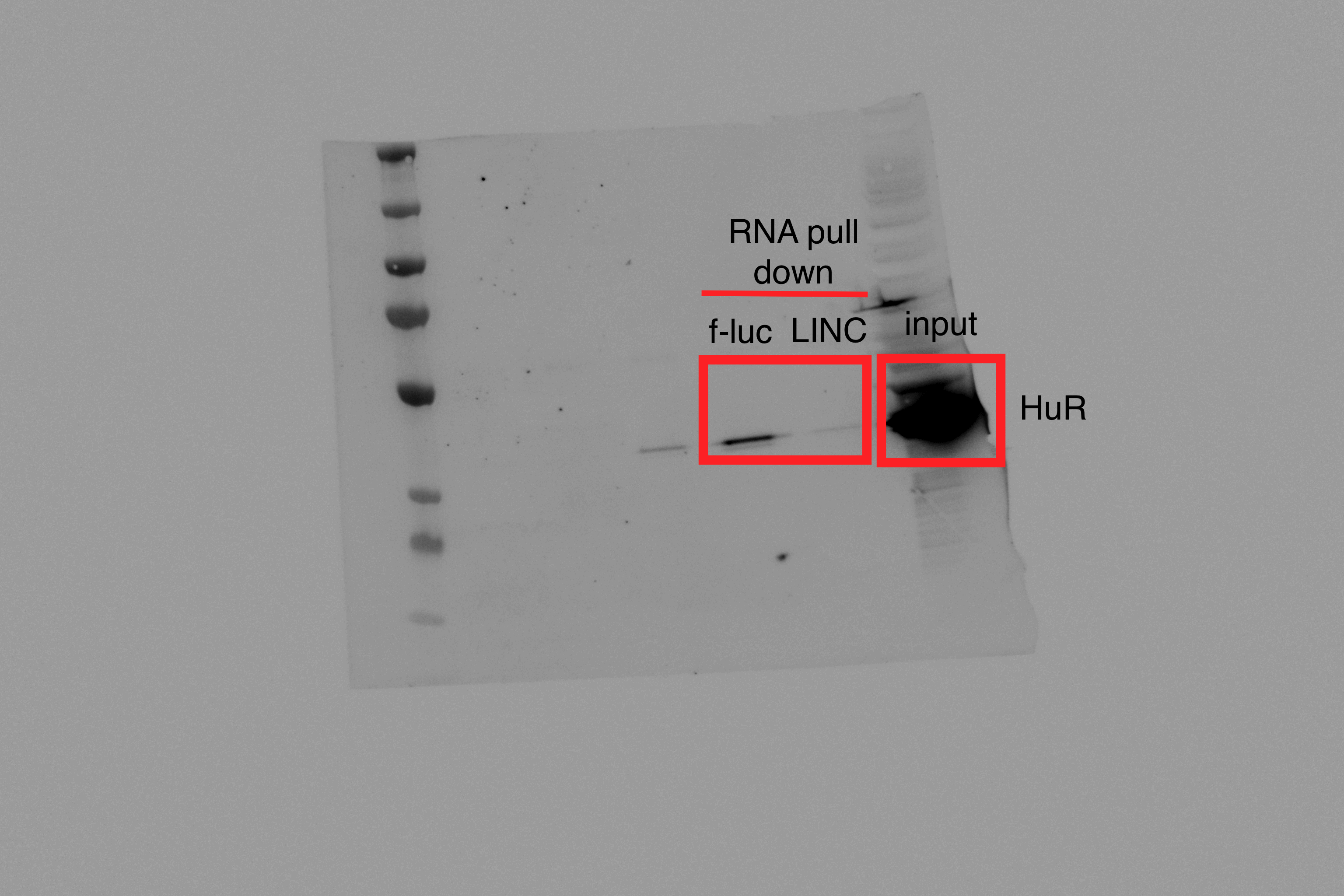

Supplement: Supplementary file 12 — Source Data Fig. 6 [file 44319_2024_75_MOESM12_ESM.zip › Figure 6/6H/RNA pull-down LINC00313 ACTL6A HuCCT1 no TGFb/HuR 6 min.tif]

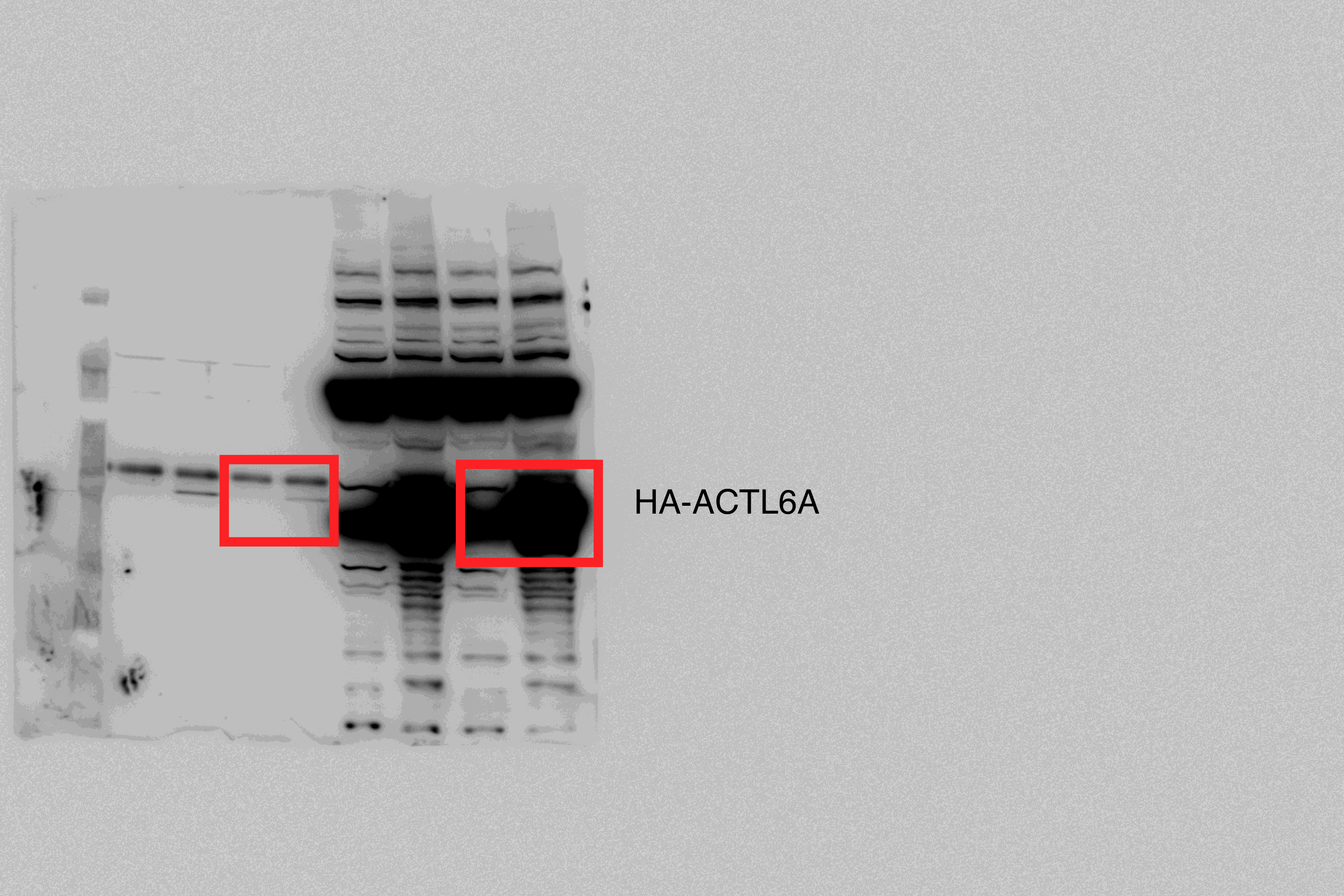

Supplement: Supplementary file 12 — Source Data Fig. 6 [file 44319_2024_75_MOESM12_ESM.zip › Figure 6/6I/HA 30 sec.tif]

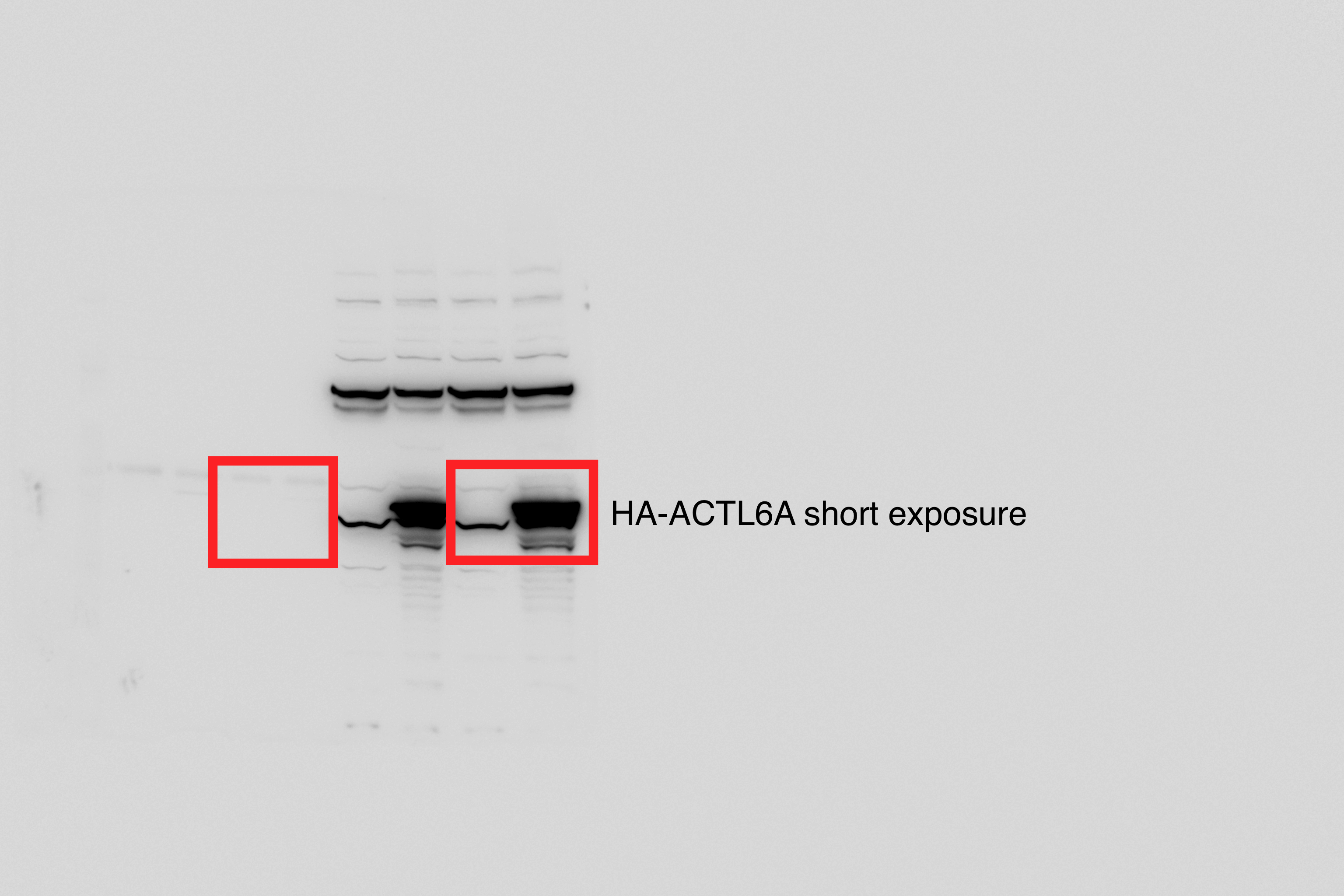

Supplement: Supplementary file 12 — Source Data Fig. 6 [file 44319_2024_75_MOESM12_ESM.zip › Figure 6/6I/HA 5 sec.tif]

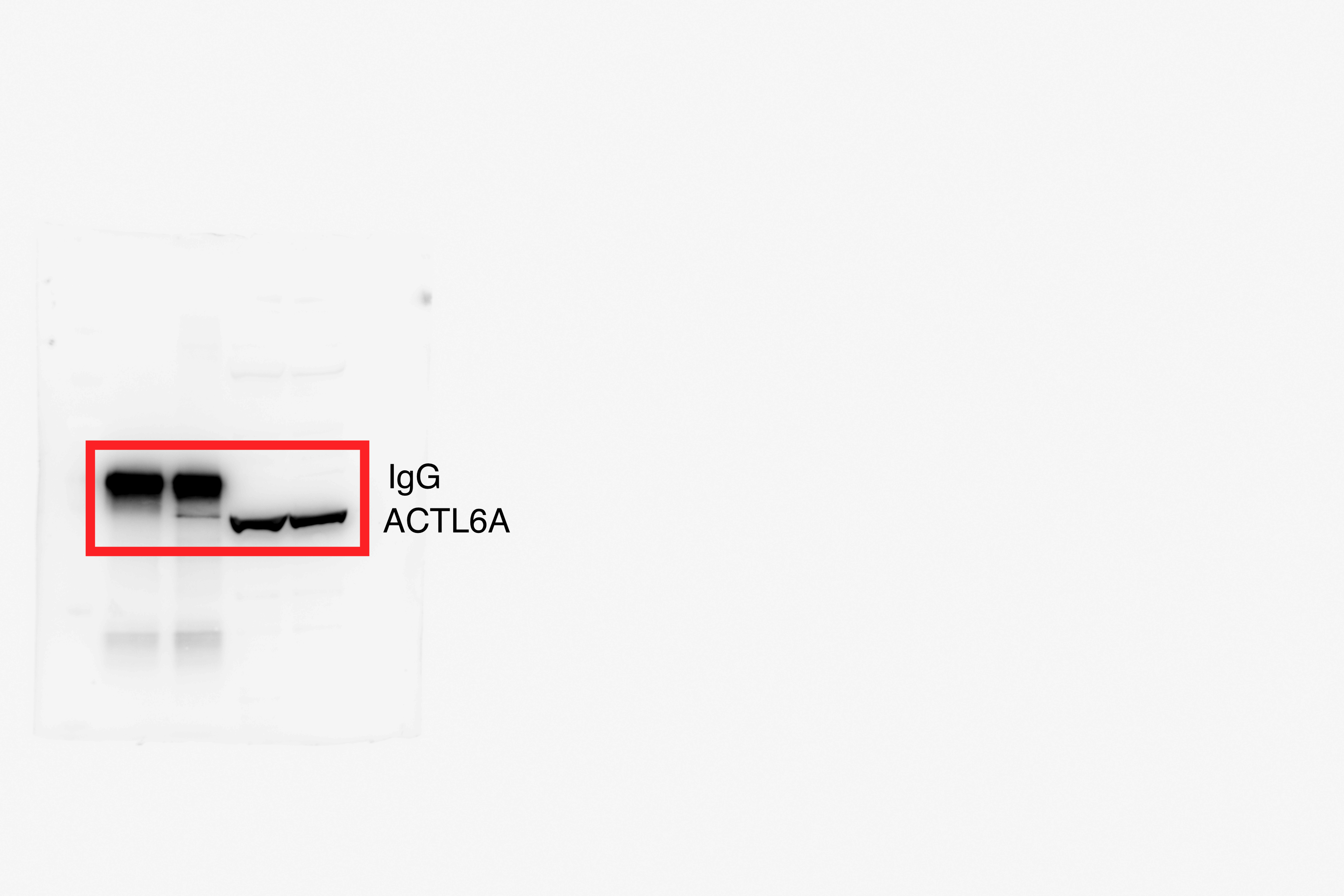

Supplement: Supplementary file 12 — Source Data Fig. 6 [file 44319_2024_75_MOESM12_ESM.zip › Figure 6/6J /ACTL6A 10 sec.tif]

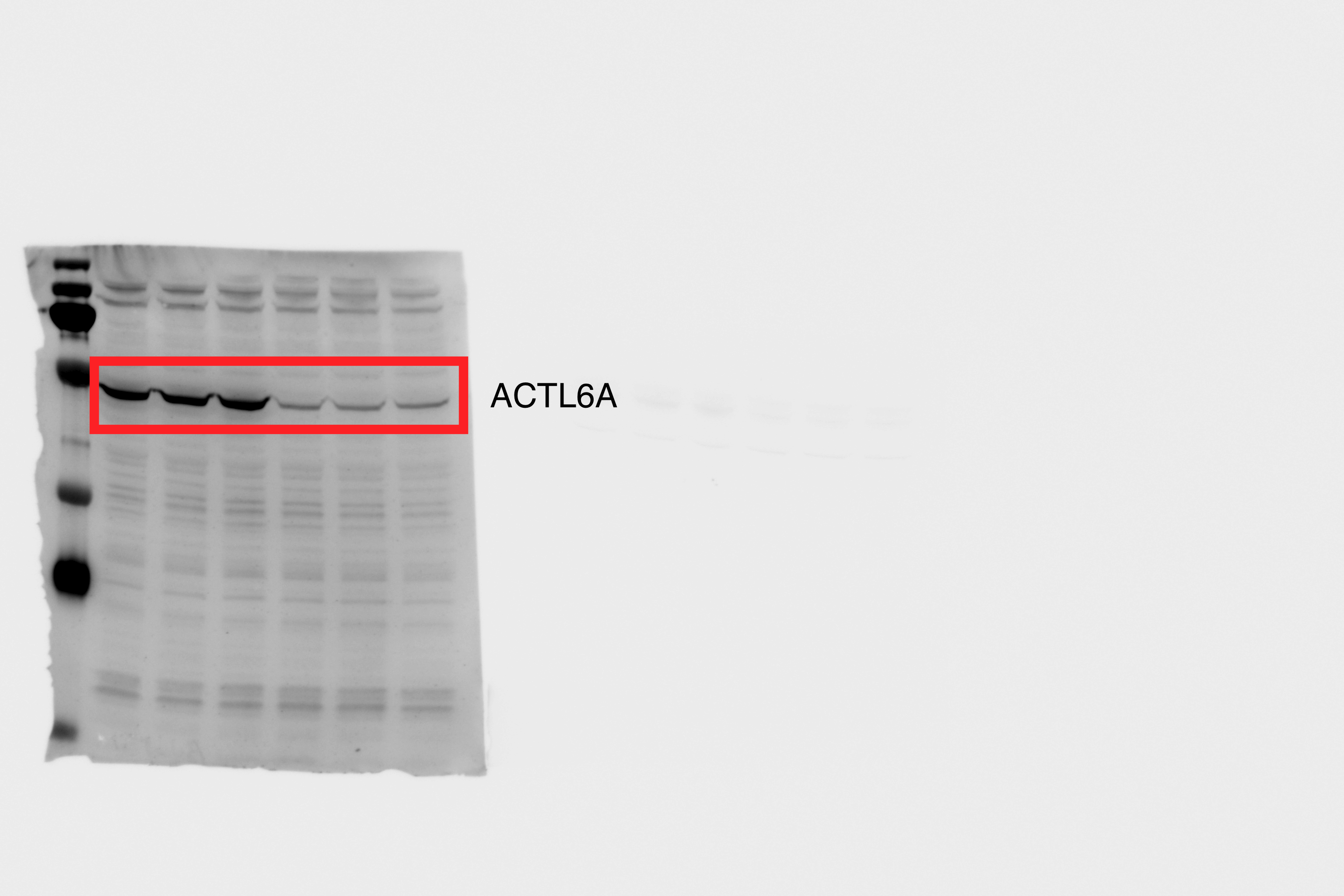

Supplement: Supplementary file 13 — Source Data Fig. 7 [file 44319_2024_75_MOESM13_ESM.zip › Figure 7/7B /WB/ACTL6A 5 sec.tif]

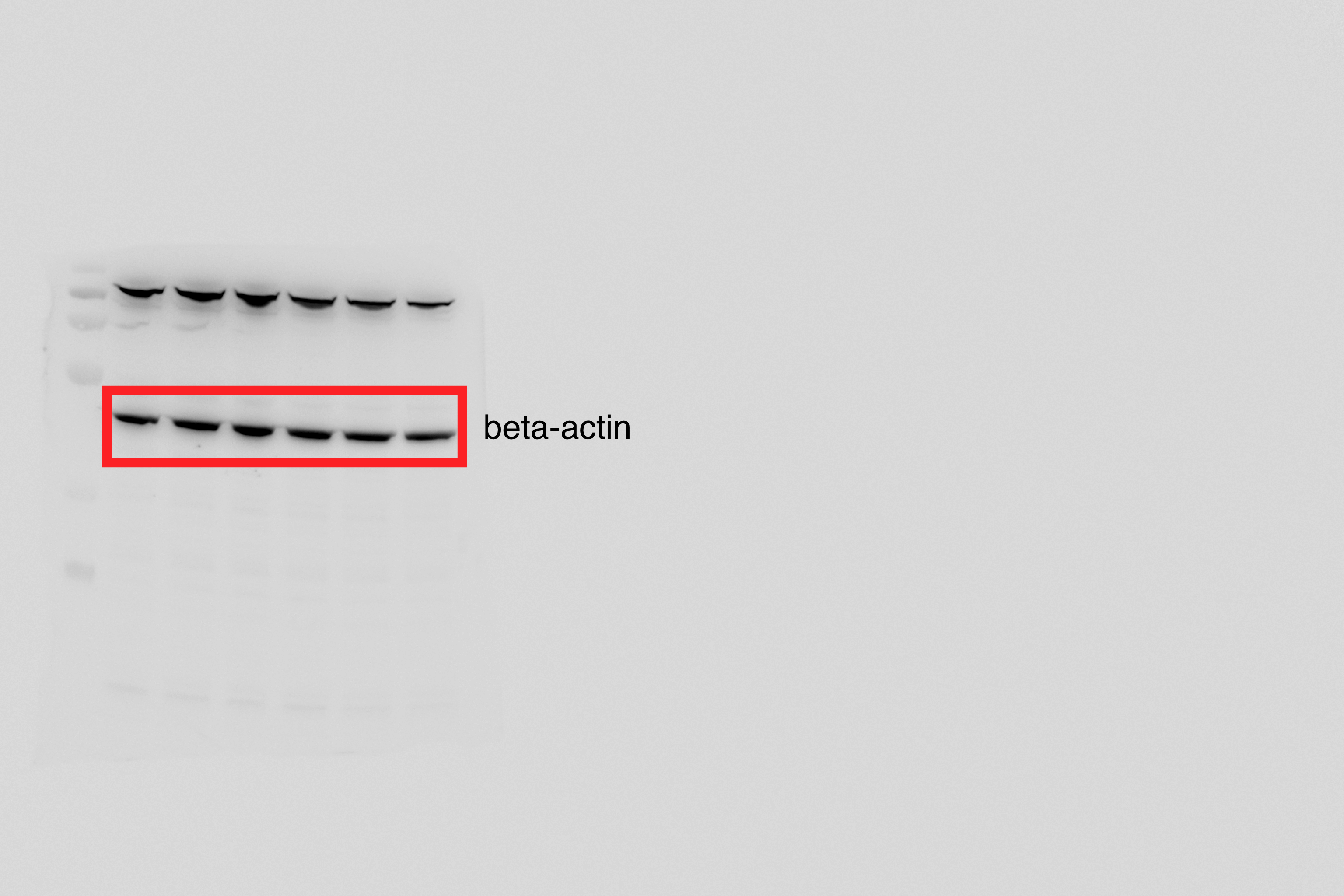

Supplement: Supplementary file 13 — Source Data Fig. 7 [file 44319_2024_75_MOESM13_ESM.zip › Figure 7/7B /WB/reblot b-actin 5 sec.tif]

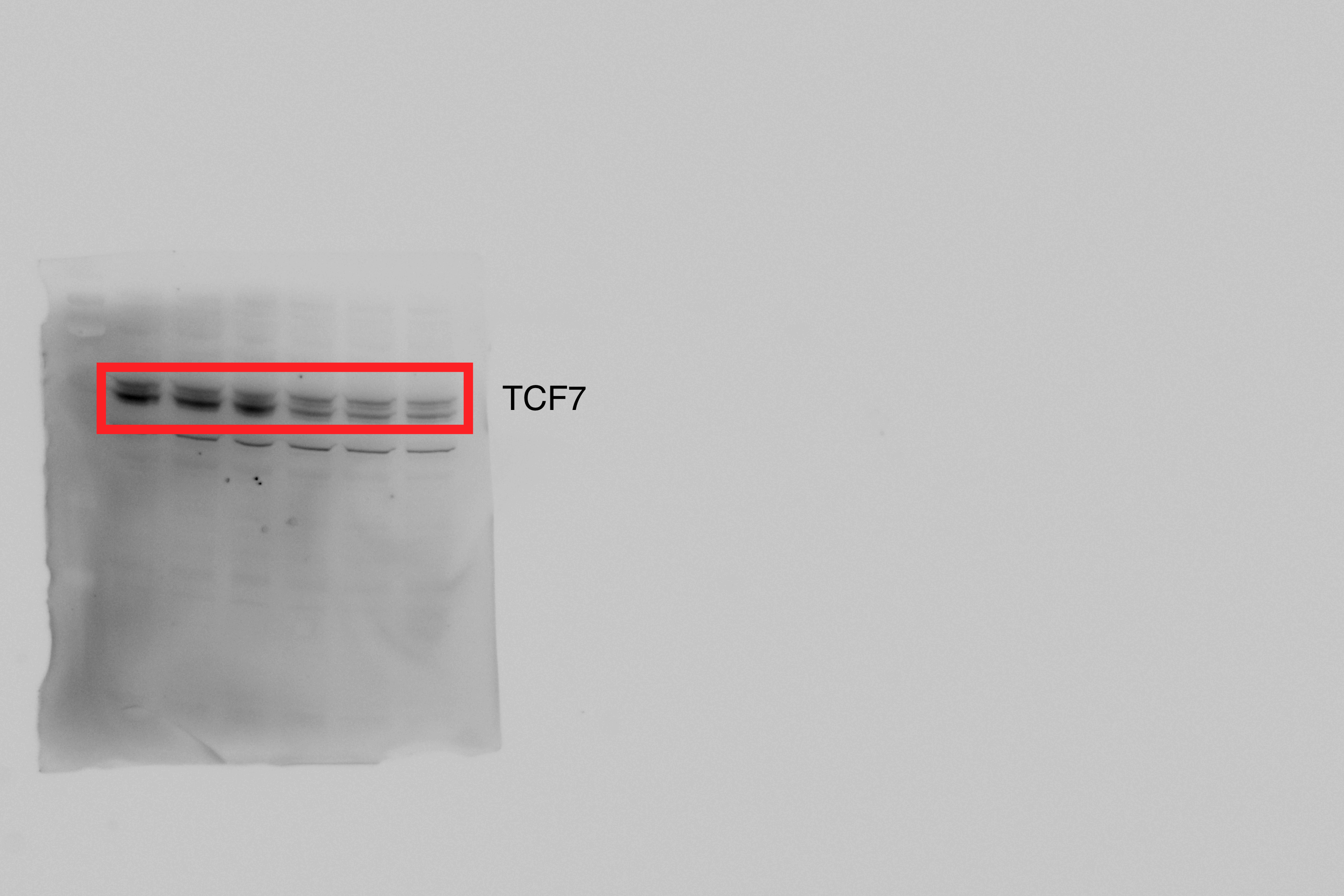

Supplement: Supplementary file 13 — Source Data Fig. 7 [file 44319_2024_75_MOESM13_ESM.zip › Figure 7/7B /WB/TCF7 30 sec.tif]

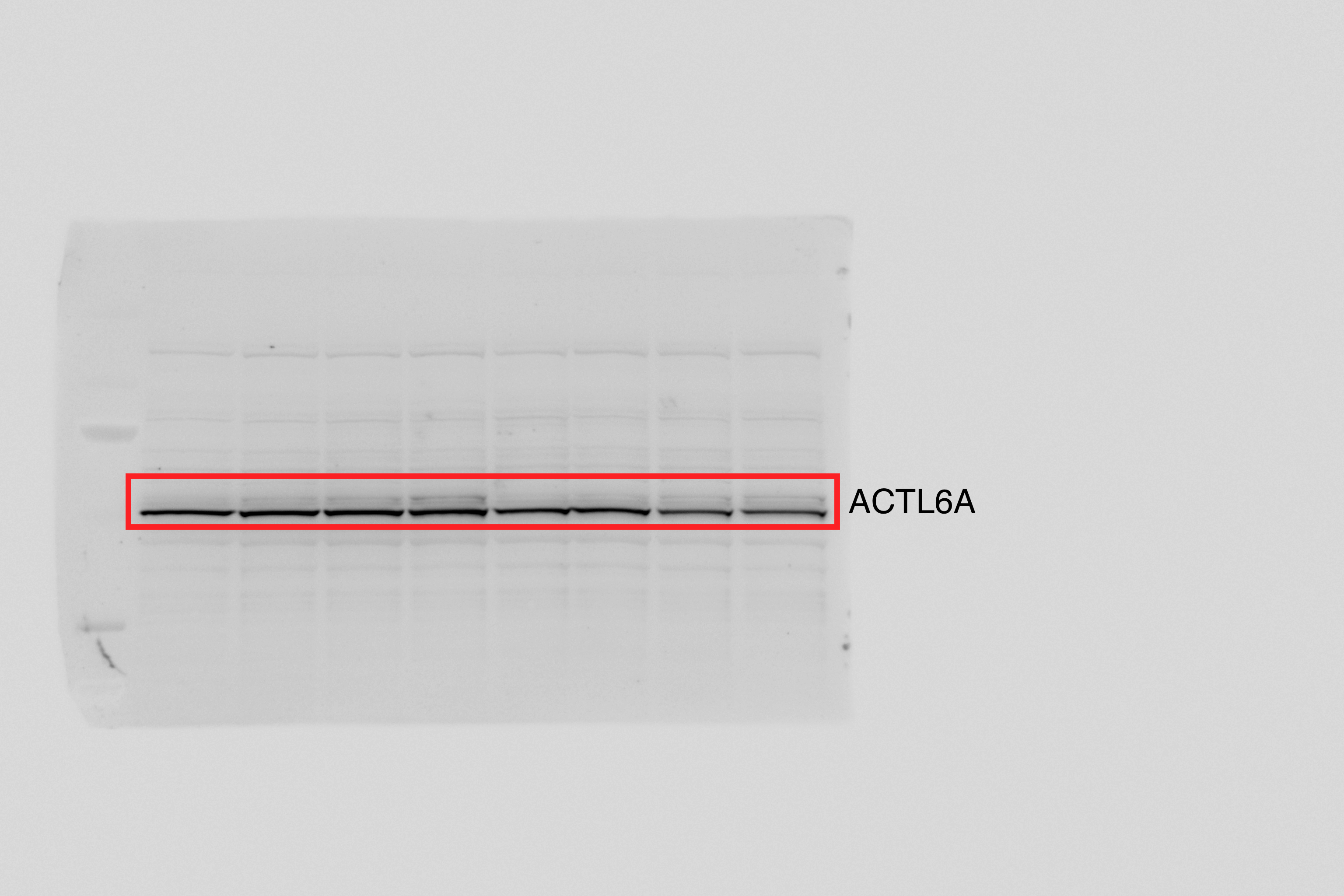

Supplement: Supplementary file 13 — Source Data Fig. 7 [file 44319_2024_75_MOESM13_ESM.zip › Figure 7/7J/WB/reblot ACTL6A 2 sec.tif]

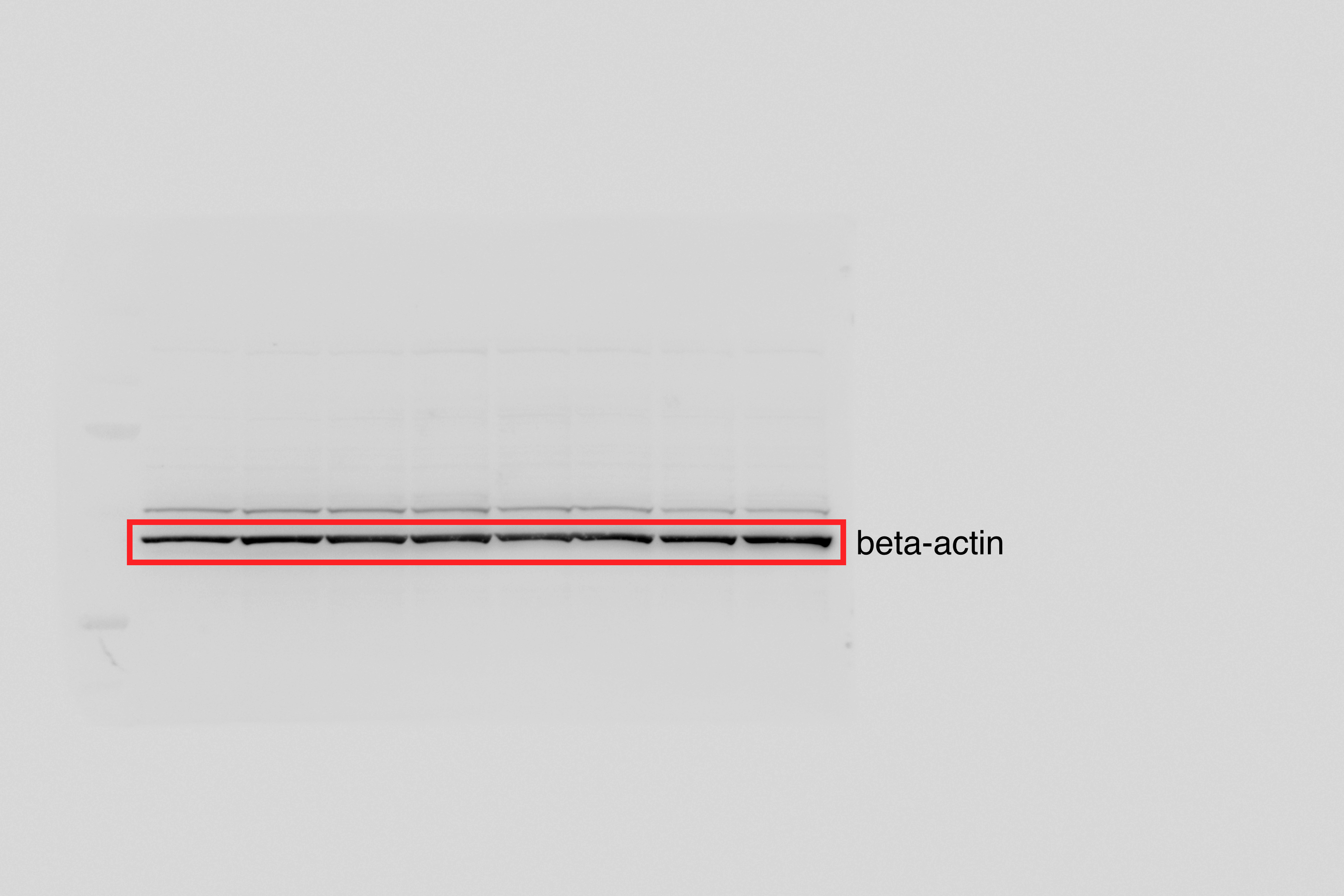

Supplement: Supplementary file 13 — Source Data Fig. 7 [file 44319_2024_75_MOESM13_ESM.zip › Figure 7/7J/WB/reblot b-actin 60 sec.tif]

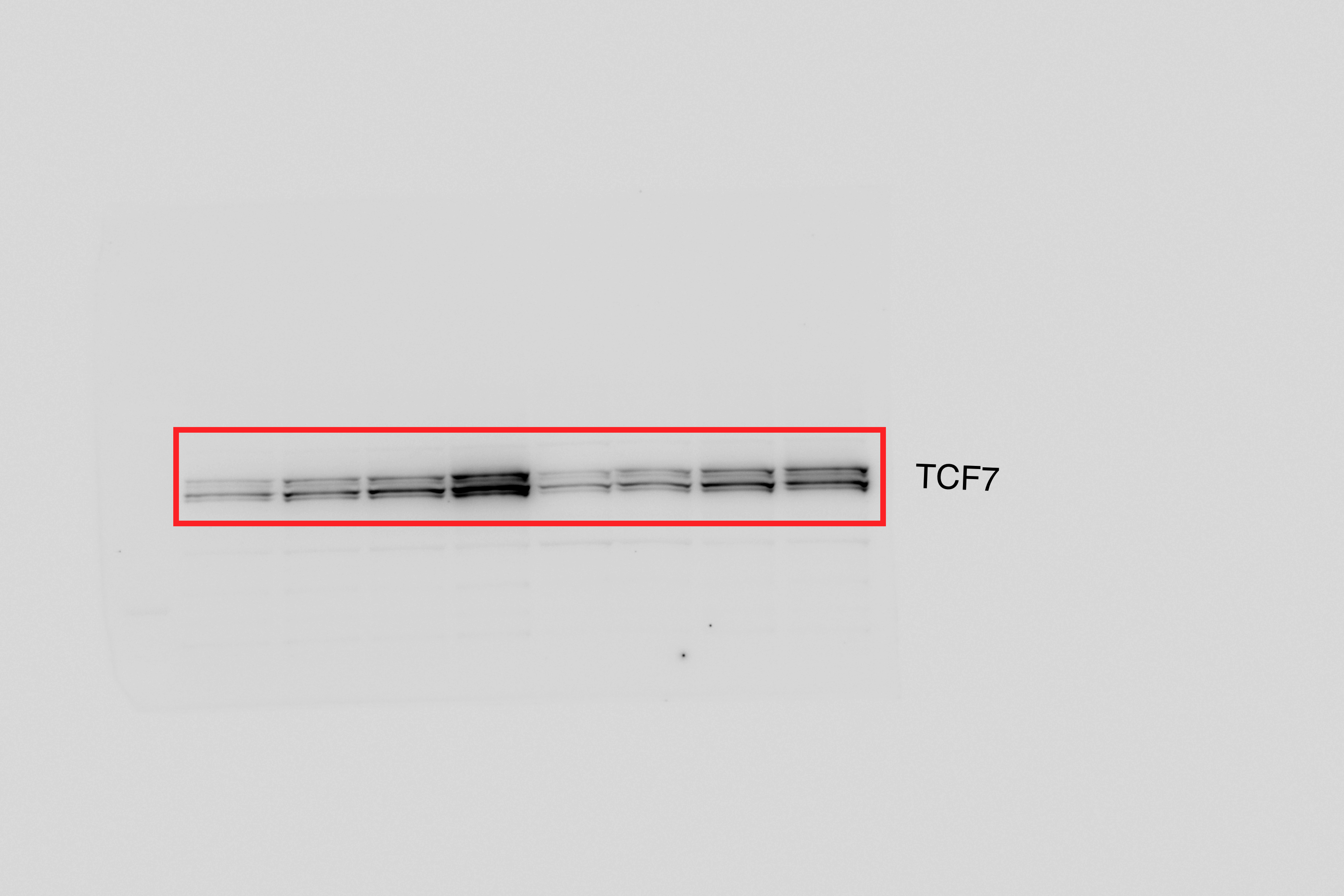

Supplement: Supplementary file 13 — Source Data Fig. 7 [file 44319_2024_75_MOESM13_ESM.zip › Figure 7/7J/WB/TCF7 10 sec.tif]

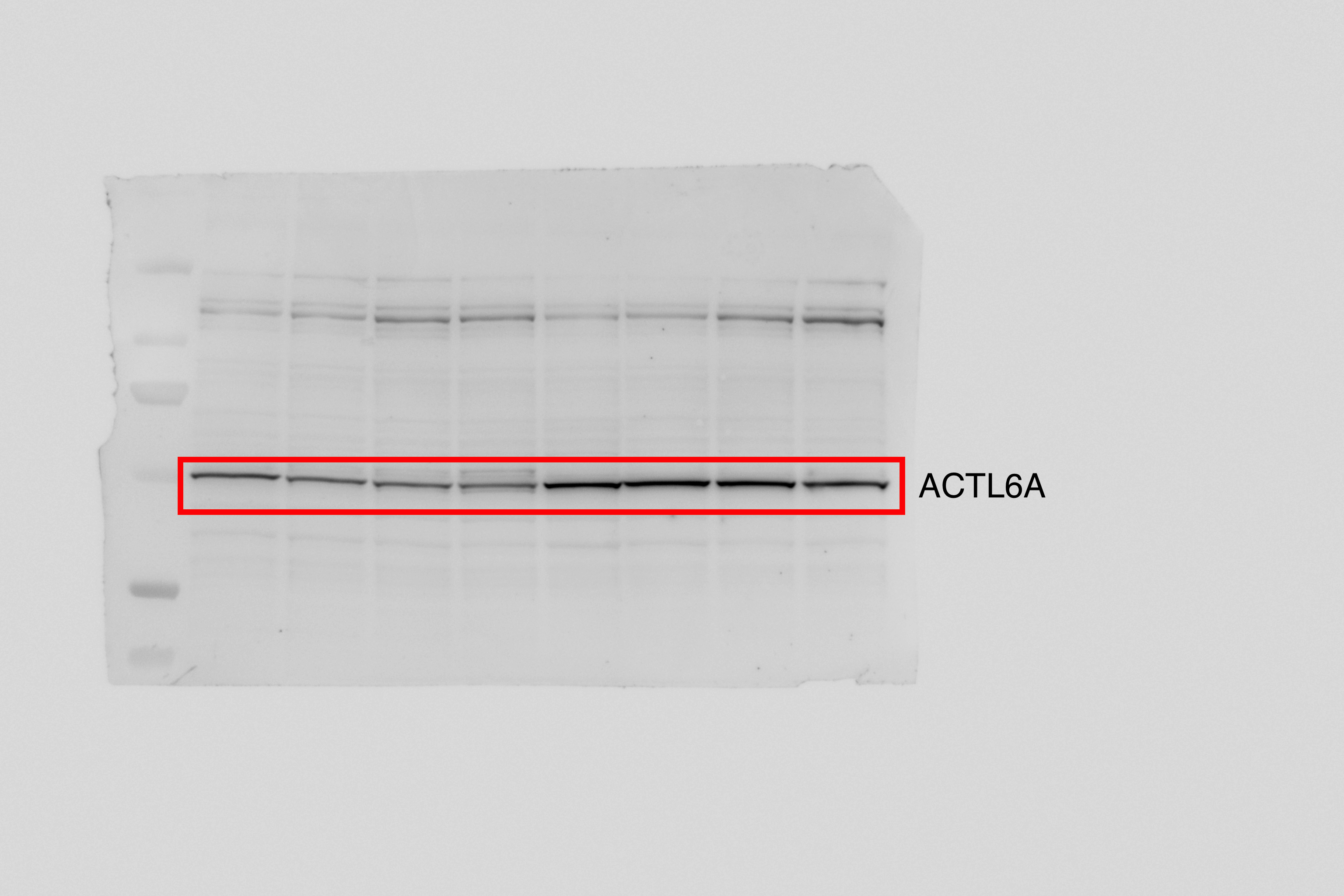

Supplement: Supplementary file 13 — Source Data Fig. 7 [file 44319_2024_75_MOESM13_ESM.zip › Figure 7/S8B (old 7F) /WB/reblot ACTL6A 2 sec.tif]

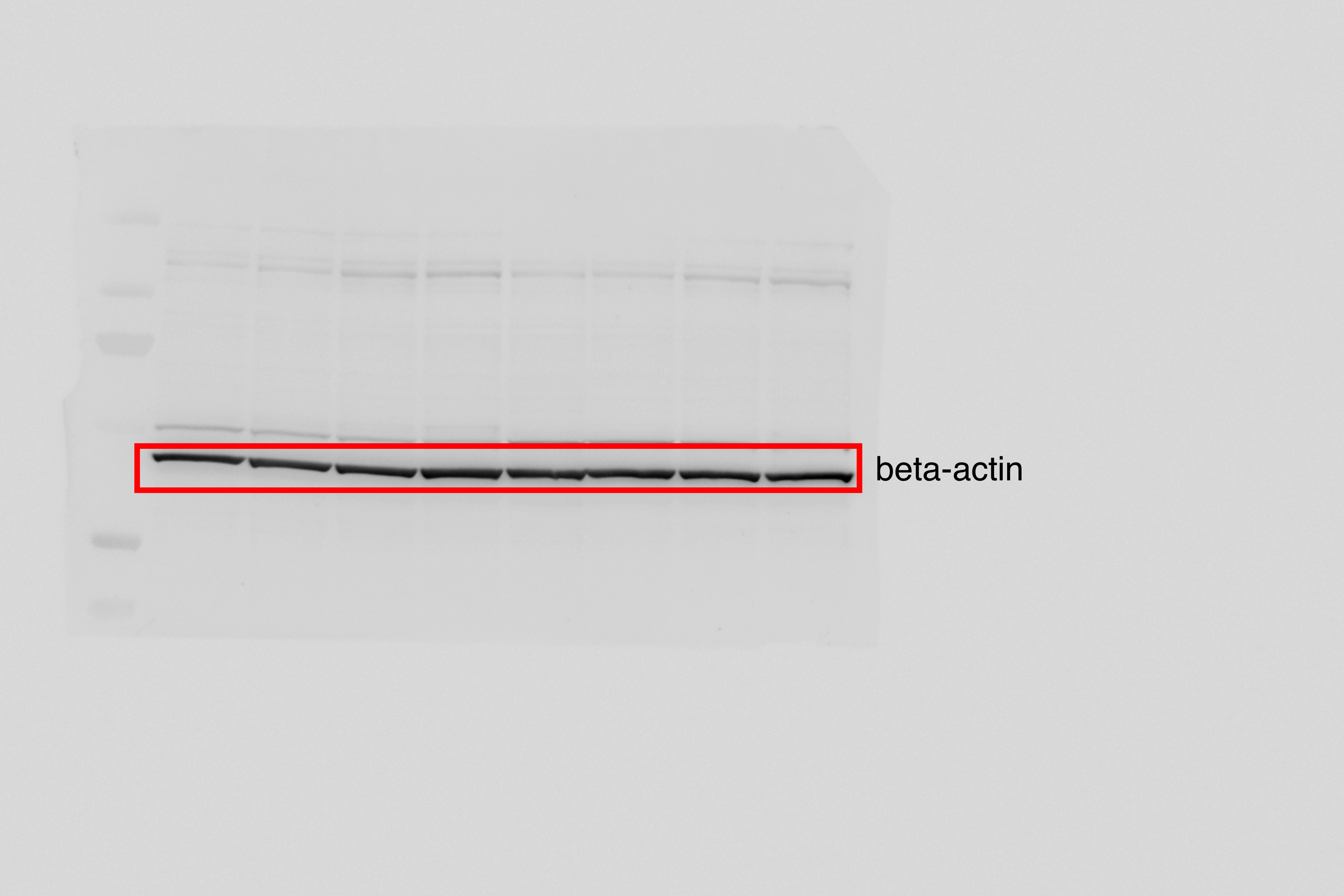

Supplement: Supplementary file 13 — Source Data Fig. 7 [file 44319_2024_75_MOESM13_ESM.zip › Figure 7/S8B (old 7F) /WB/reblot b-actin 45 sec.tif]

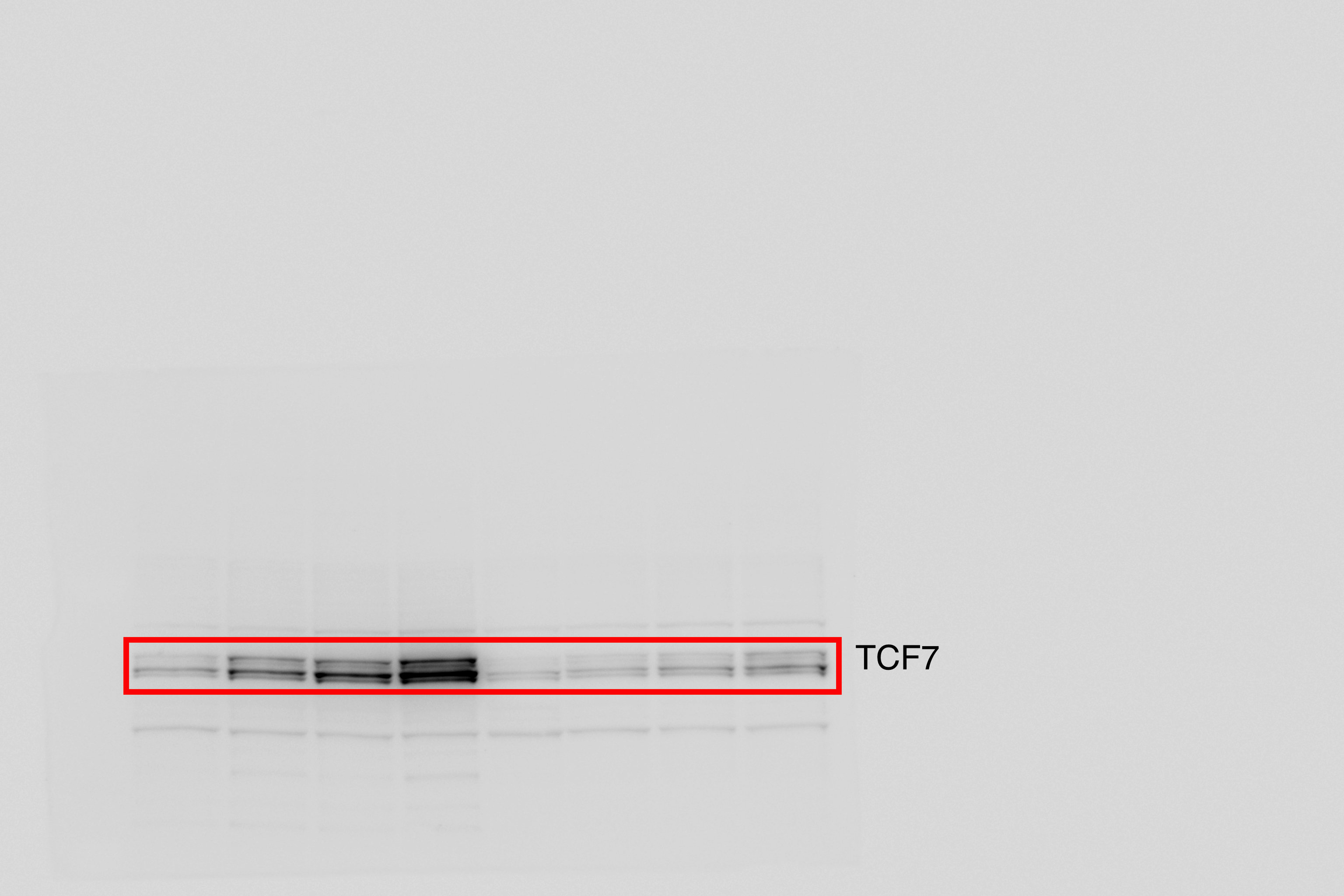

Supplement: Supplementary file 13 — Source Data Fig. 7 [file 44319_2024_75_MOESM13_ESM.zip › Figure 7/S8B (old 7F) /WB/TCF7 15 sec.tif]

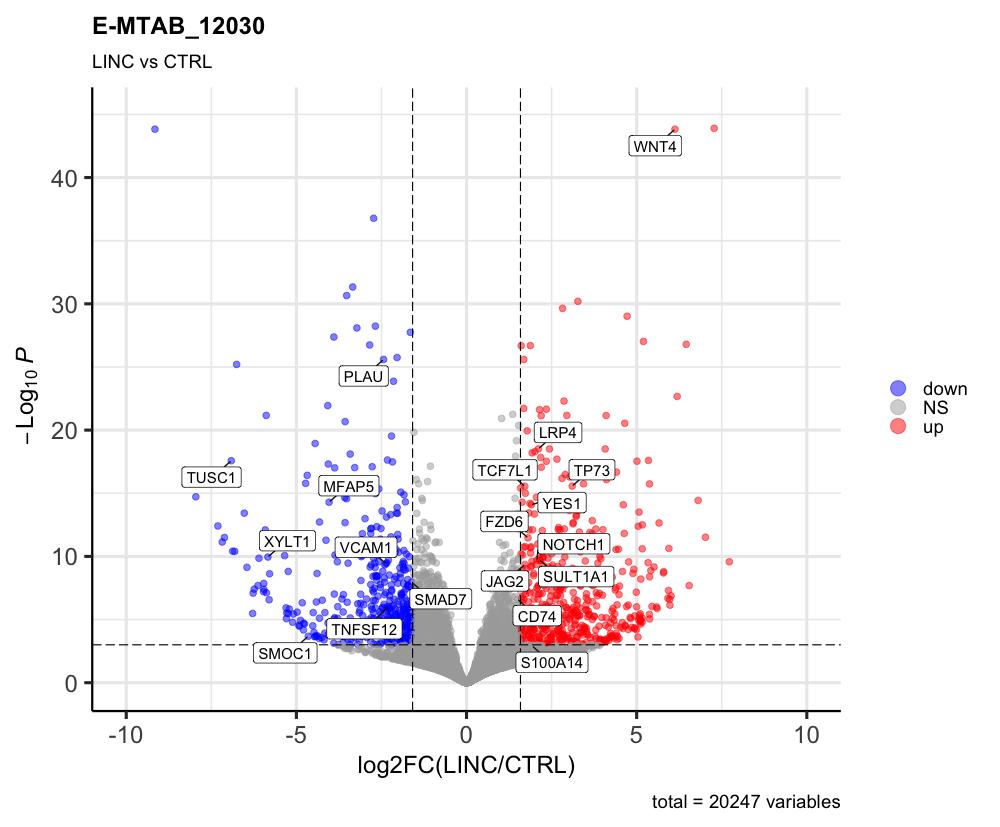

Supplement: Supplementary file 14 — Source Data Fig. 8 [file 44319_2024_75_MOESM14_ESM.zip › Figure 8/volcano_fig8A.tiff]

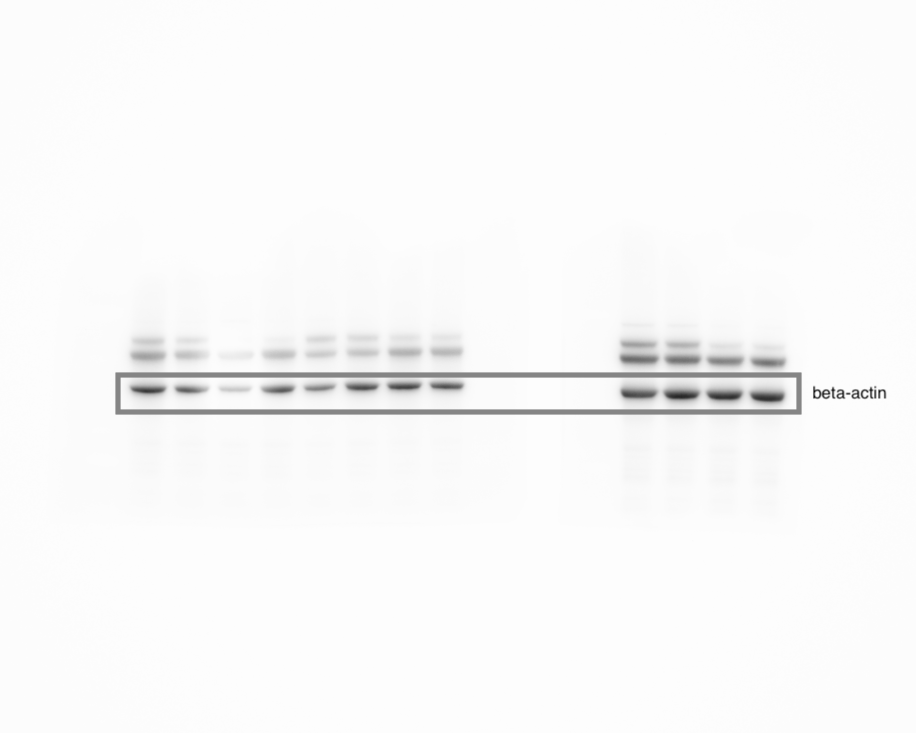

Supplement: Supplementary file 15 — Appendix and EV Figure Source Data [file 44319_2024_75_MOESM15_ESM.zip › Figure Appendix S2/S2B/reblot_beta-actin 1.5sec.tif]

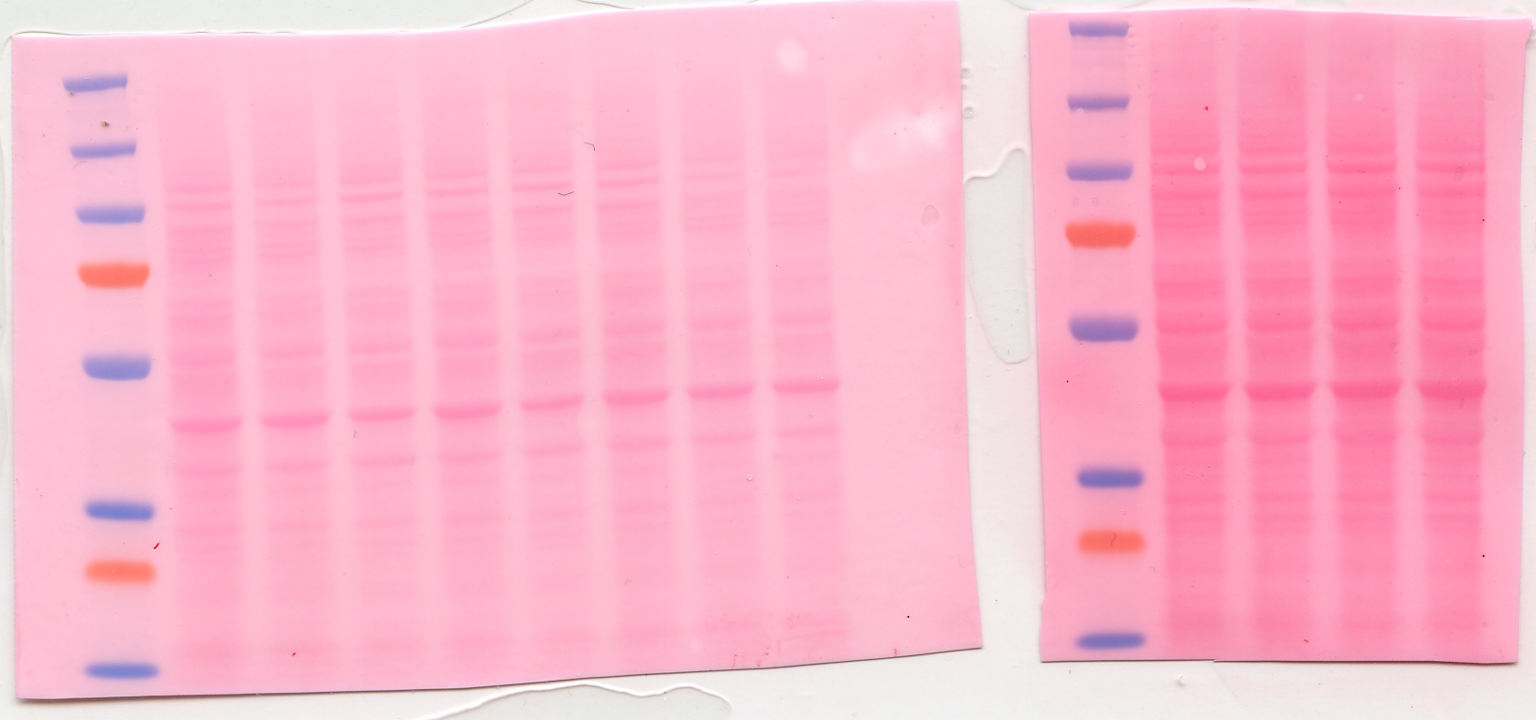

Supplement: Supplementary file 15 — Appendix and EV Figure Source Data [file 44319_2024_75_MOESM15_ESM.zip › Figure Appendix S2/S2B/sismads_ponceau.tif]

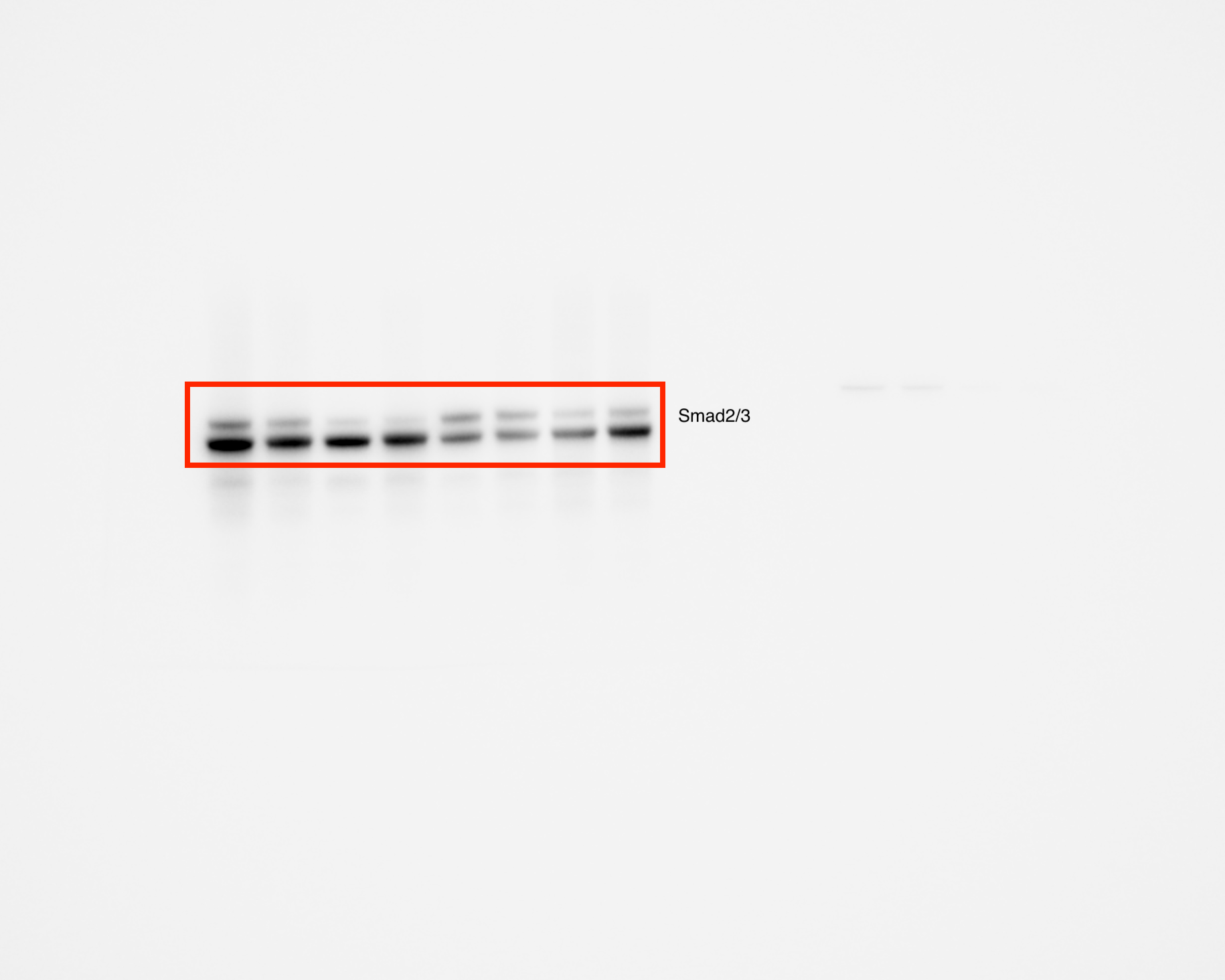

Supplement: Supplementary file 15 — Appendix and EV Figure Source Data [file 44319_2024_75_MOESM15_ESM.zip › Figure Appendix S2/S2B/Smad23 smad4 2sec.tif]

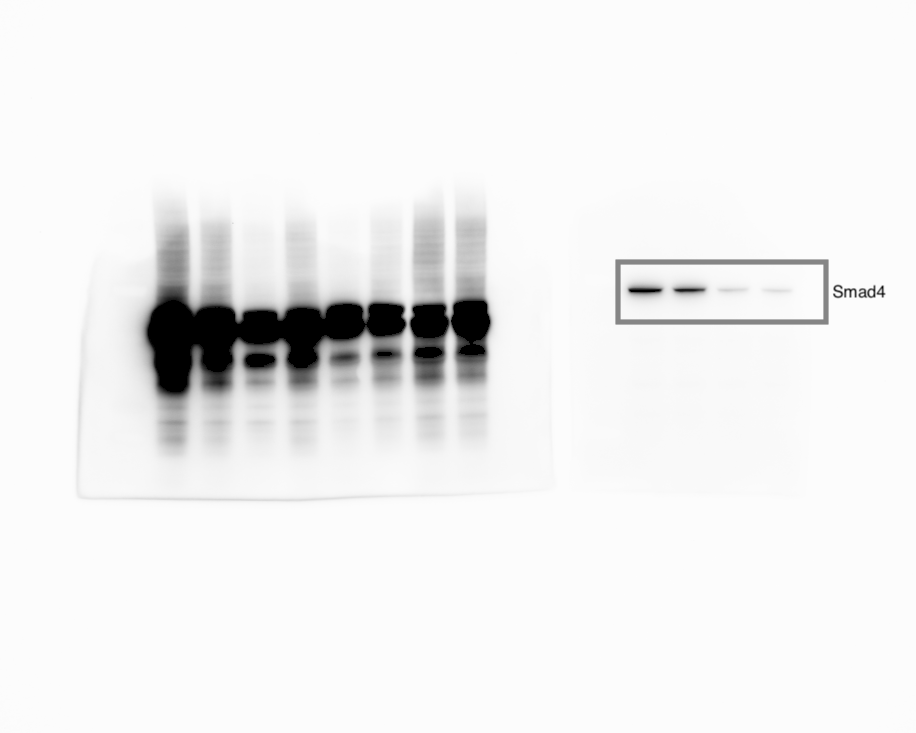

Supplement: Supplementary file 15 — Appendix and EV Figure Source Data [file 44319_2024_75_MOESM15_ESM.zip › Figure Appendix S2/S2B/smad23 smad4 60sec.tif]

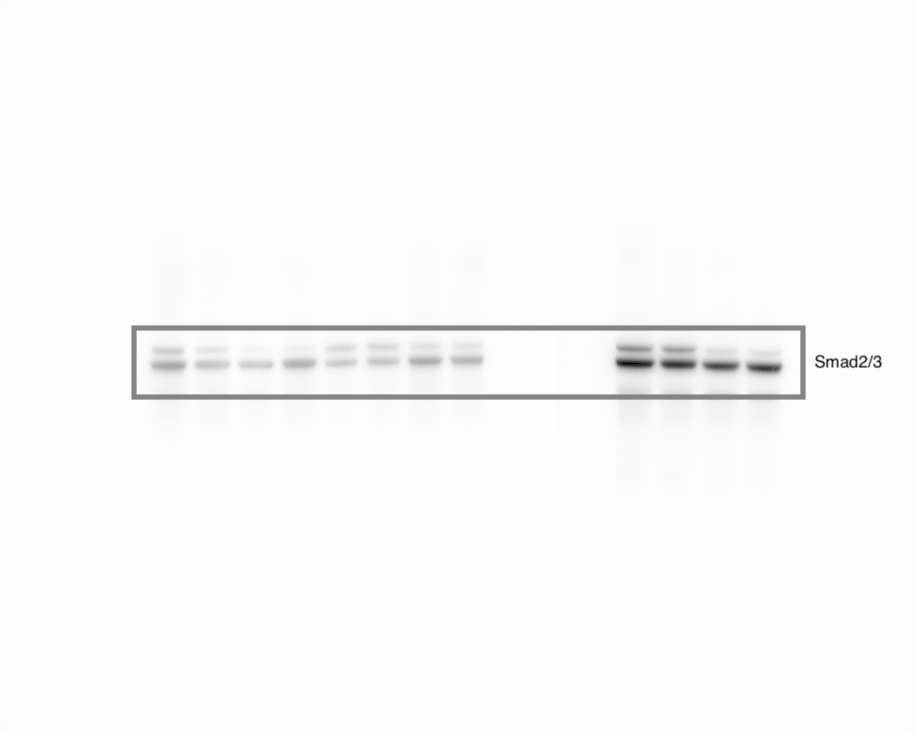

Supplement: Supplementary file 15 — Appendix and EV Figure Source Data [file 44319_2024_75_MOESM15_ESM.zip › Figure Appendix S2/S2B/smad4 smad23 1sec.tif]

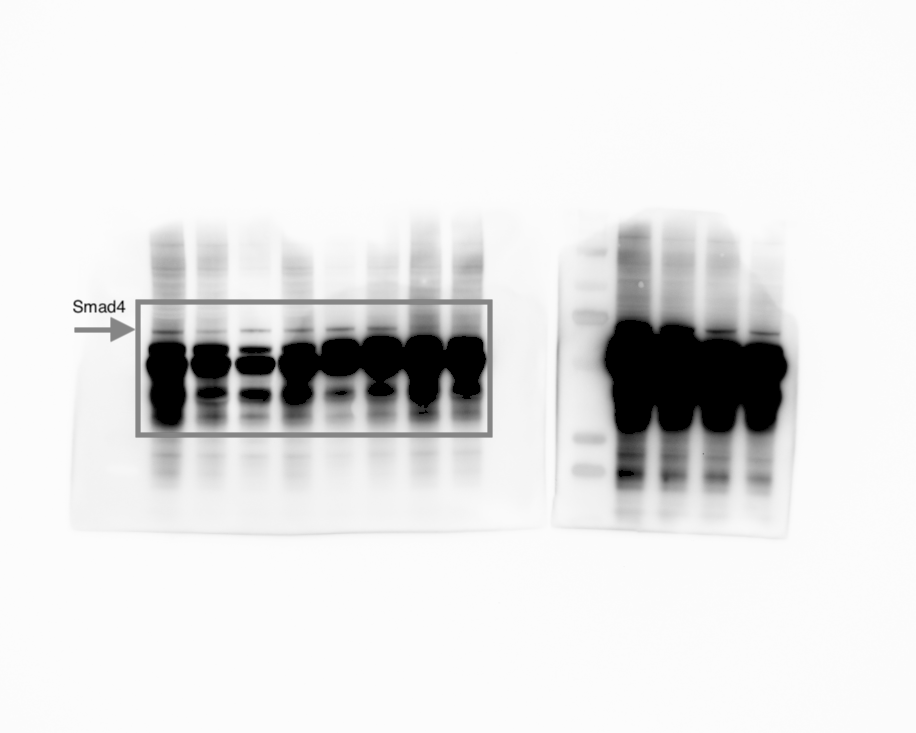

Supplement: Supplementary file 15 — Appendix and EV Figure Source Data [file 44319_2024_75_MOESM15_ESM.zip › Figure Appendix S2/S2B/smad4 smad23 60sec.tif]

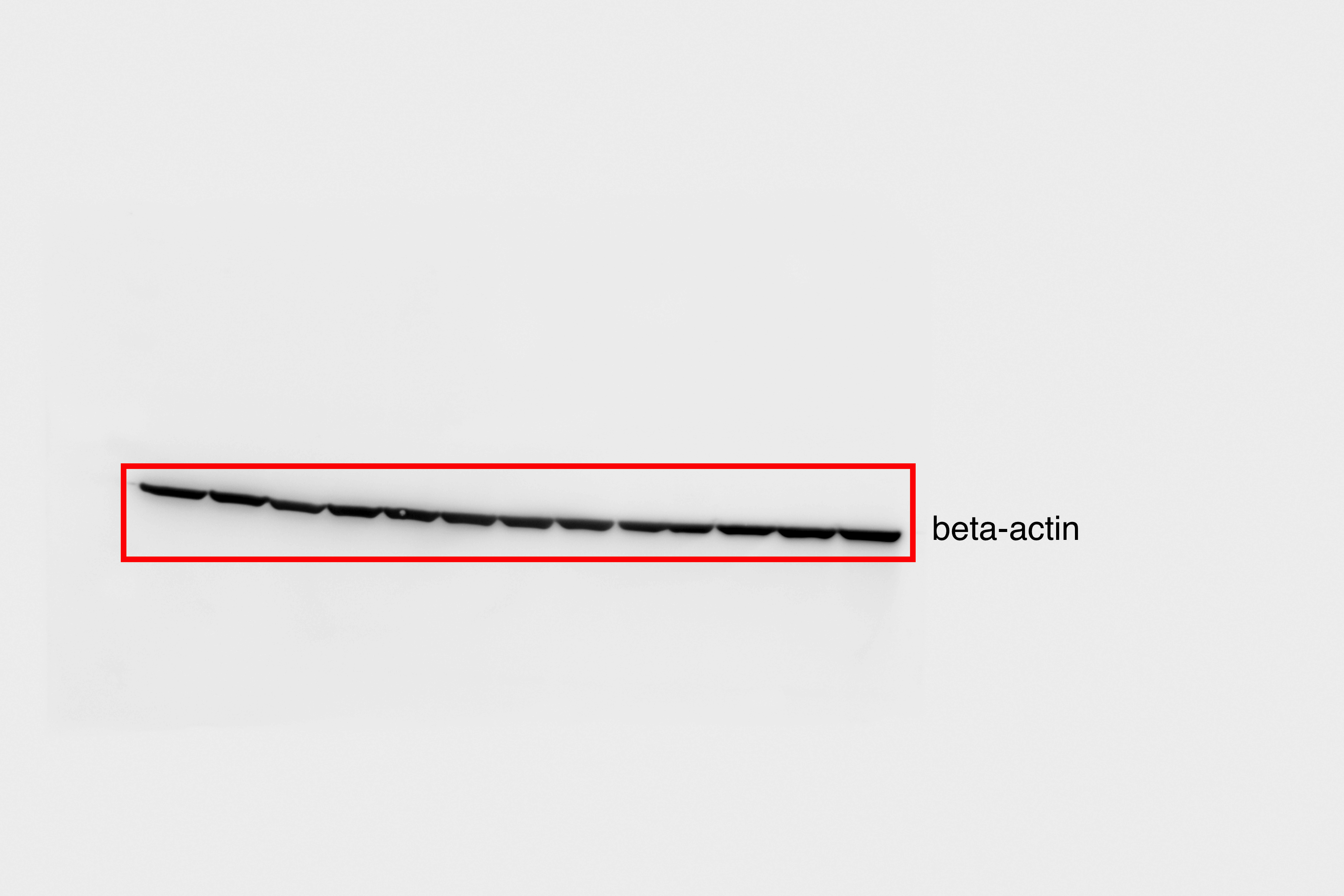

Supplement: Supplementary file 15 — Appendix and EV Figure Source Data [file 44319_2024_75_MOESM15_ESM.zip › Figure Appendix S7/S7B/reblot b-actin 1 sec.tif]

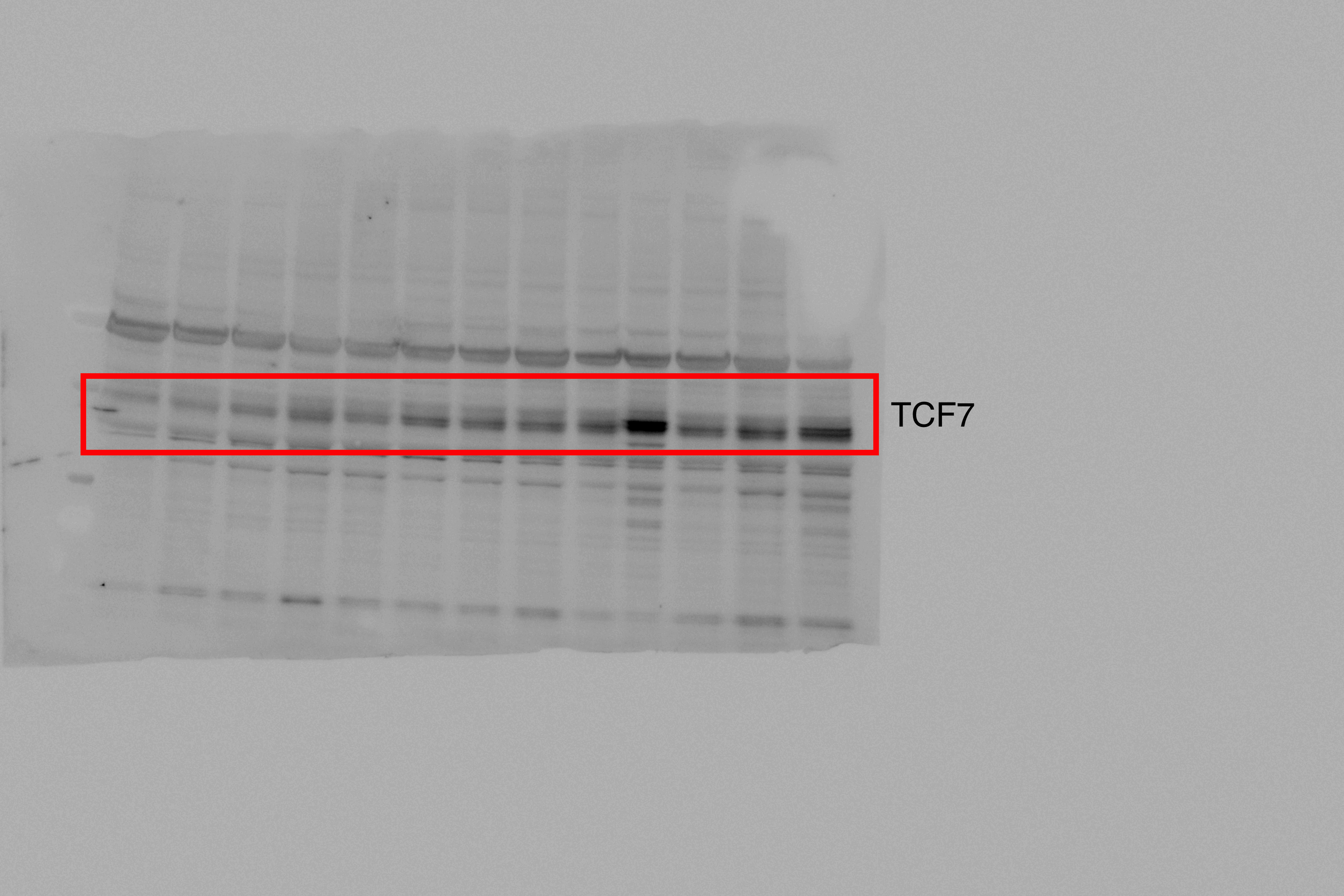

Supplement: Supplementary file 15 — Appendix and EV Figure Source Data [file 44319_2024_75_MOESM15_ESM.zip › Figure Appendix S7/S7B/TCF7 30 sec densitometry.tif]

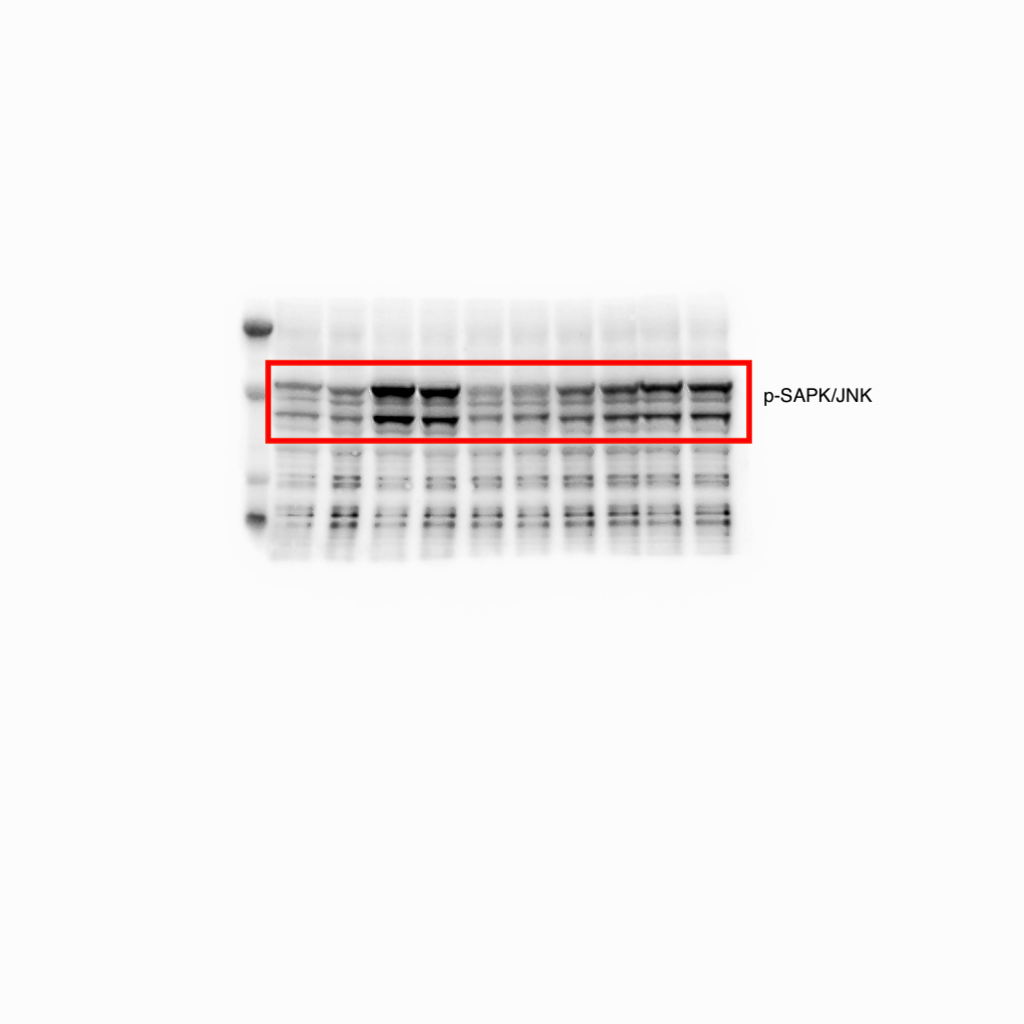

Supplement: Supplementary file 15 — Appendix and EV Figure Source Data [file 44319_2024_75_MOESM15_ESM.zip › Figure EV2/EV2B/p-JNK 3 sec.Tif]

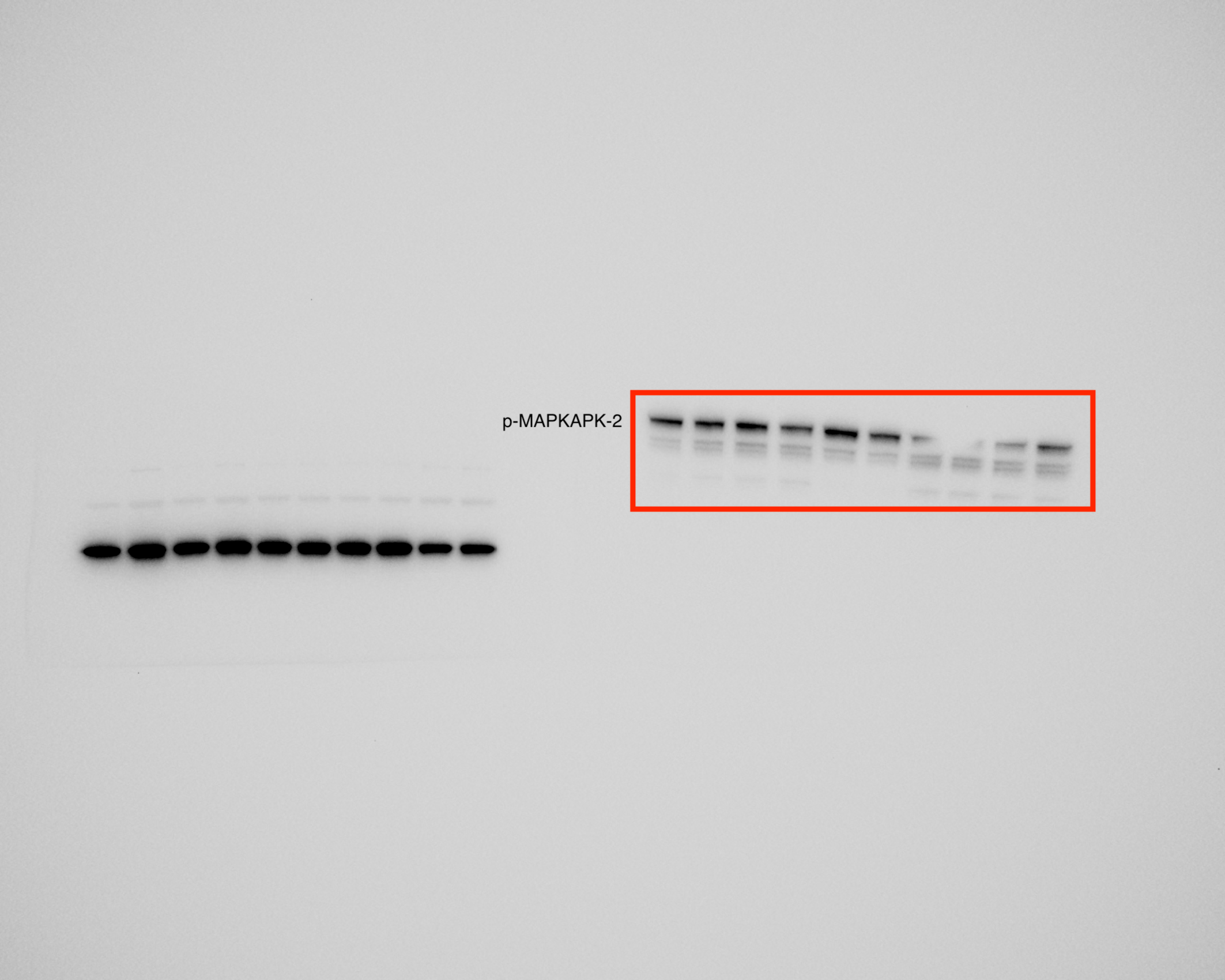

Supplement: Supplementary file 15 — Appendix and EV Figure Source Data [file 44319_2024_75_MOESM15_ESM.zip › Figure EV2/EV2B/p-MAPKAPK-2 5sec.tif]

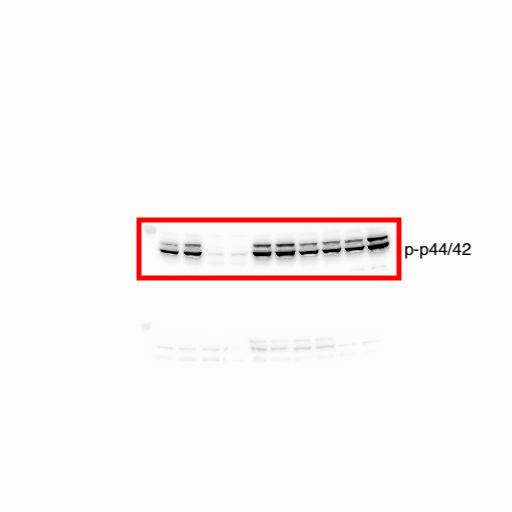

Supplement: Supplementary file 15 — Appendix and EV Figure Source Data [file 44319_2024_75_MOESM15_ESM.zip › Figure EV2/EV2B/p-p44 42.Tif]

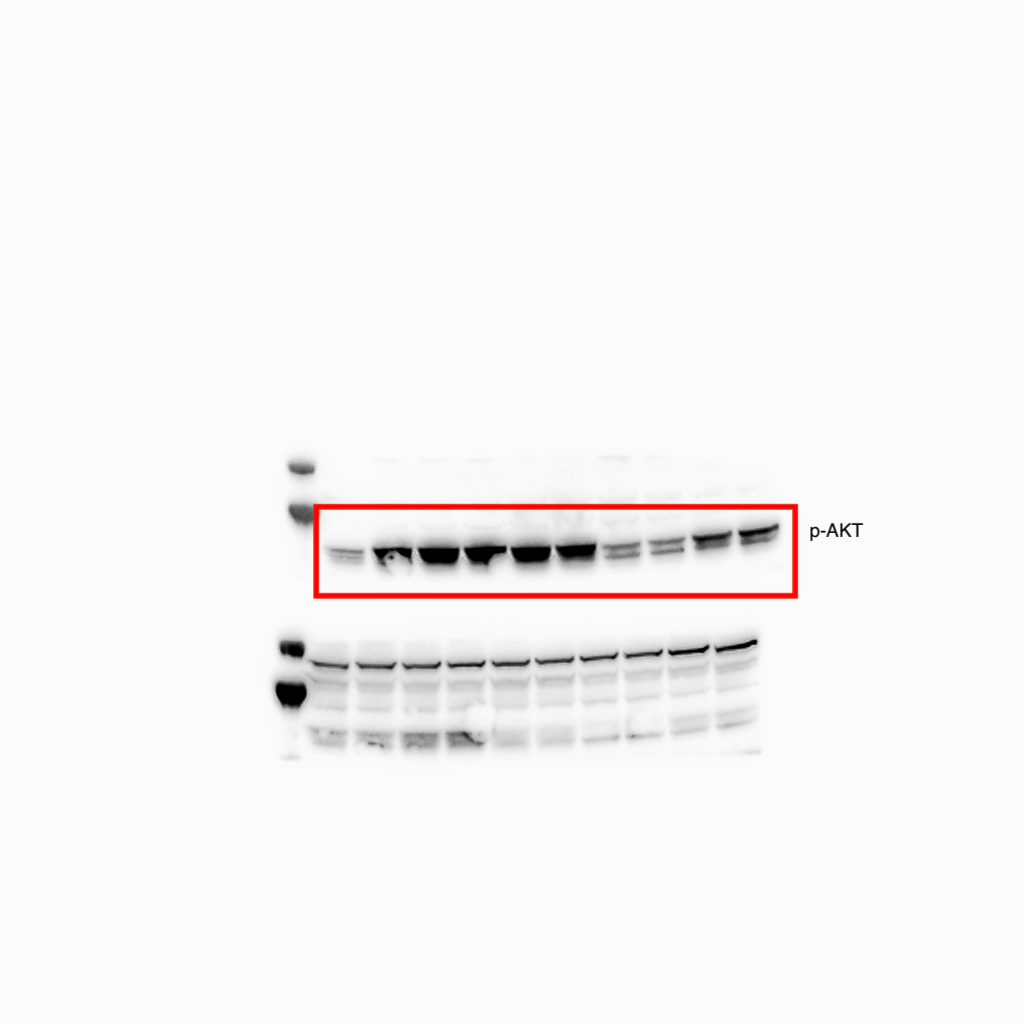

Supplement: Supplementary file 15 — Appendix and EV Figure Source Data [file 44319_2024_75_MOESM15_ESM.zip › Figure EV2/EV2B/pAKT 10sec.Tif]

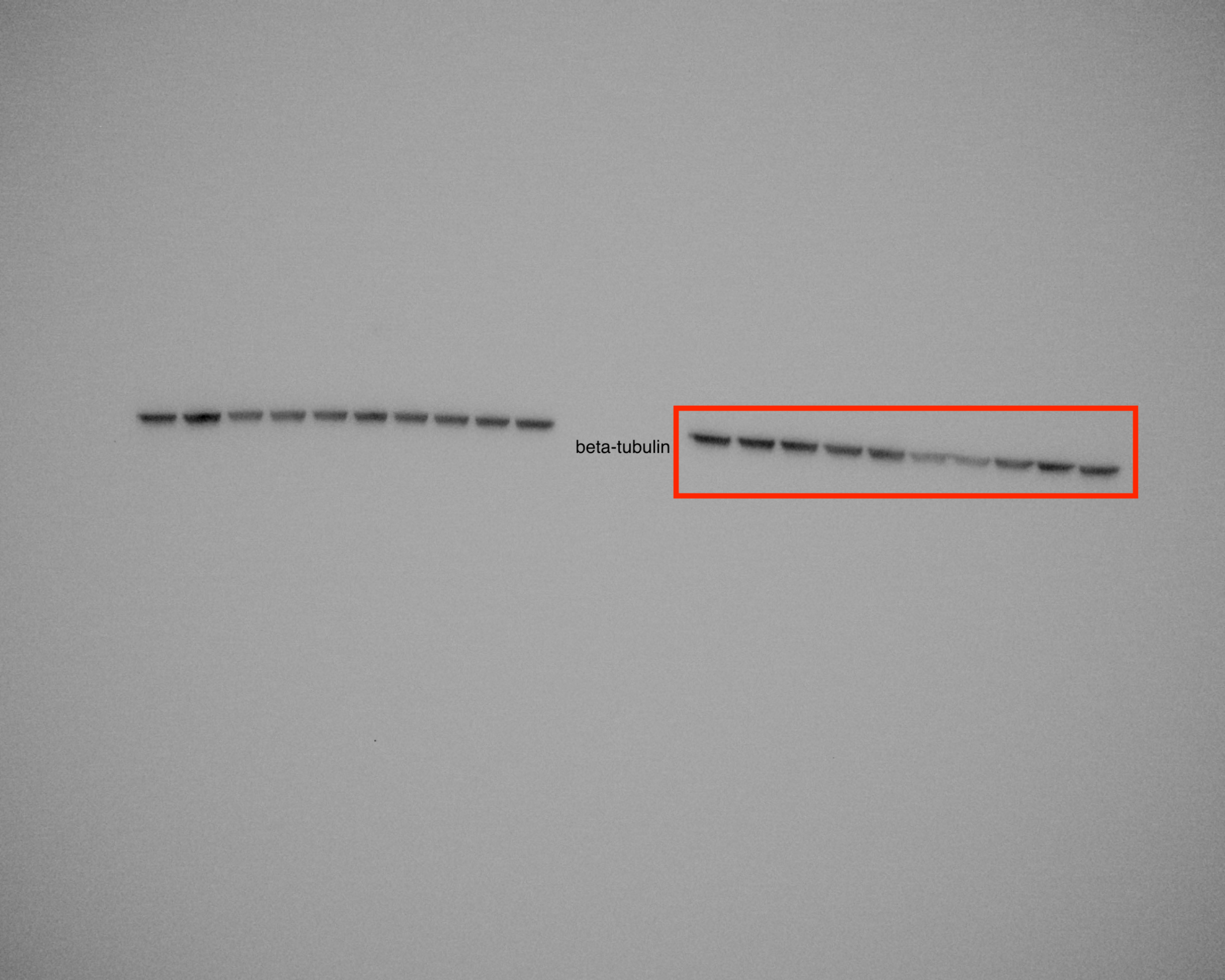

Supplement: Supplementary file 15 — Appendix and EV Figure Source Data [file 44319_2024_75_MOESM15_ESM.zip › Figure EV2/EV2B/reblot beta-tubulin 10sec.tif]

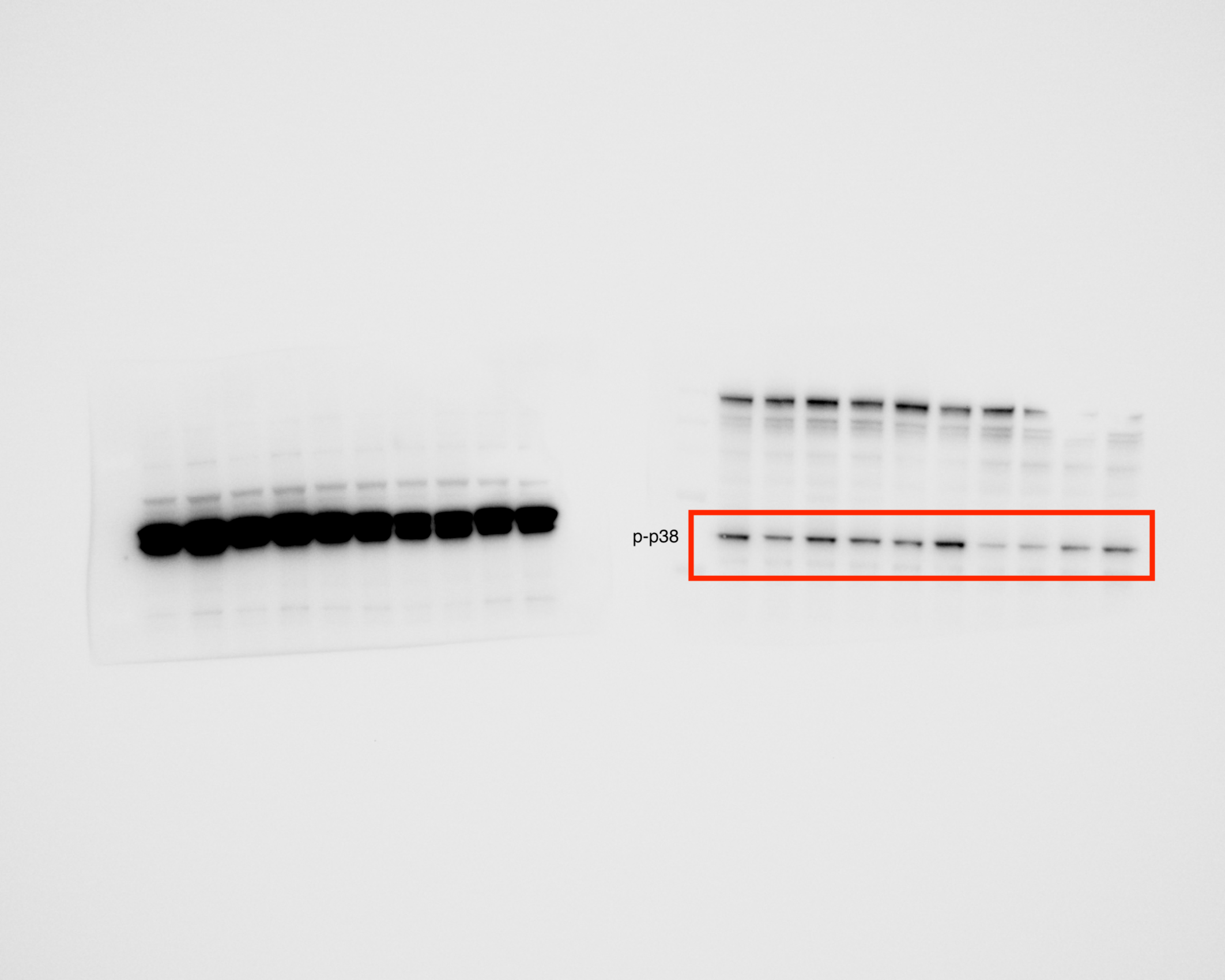

Supplement: Supplementary file 15 — Appendix and EV Figure Source Data [file 44319_2024_75_MOESM15_ESM.zip › Figure EV2/EV2B/reblot p-p38 10sec.tif]

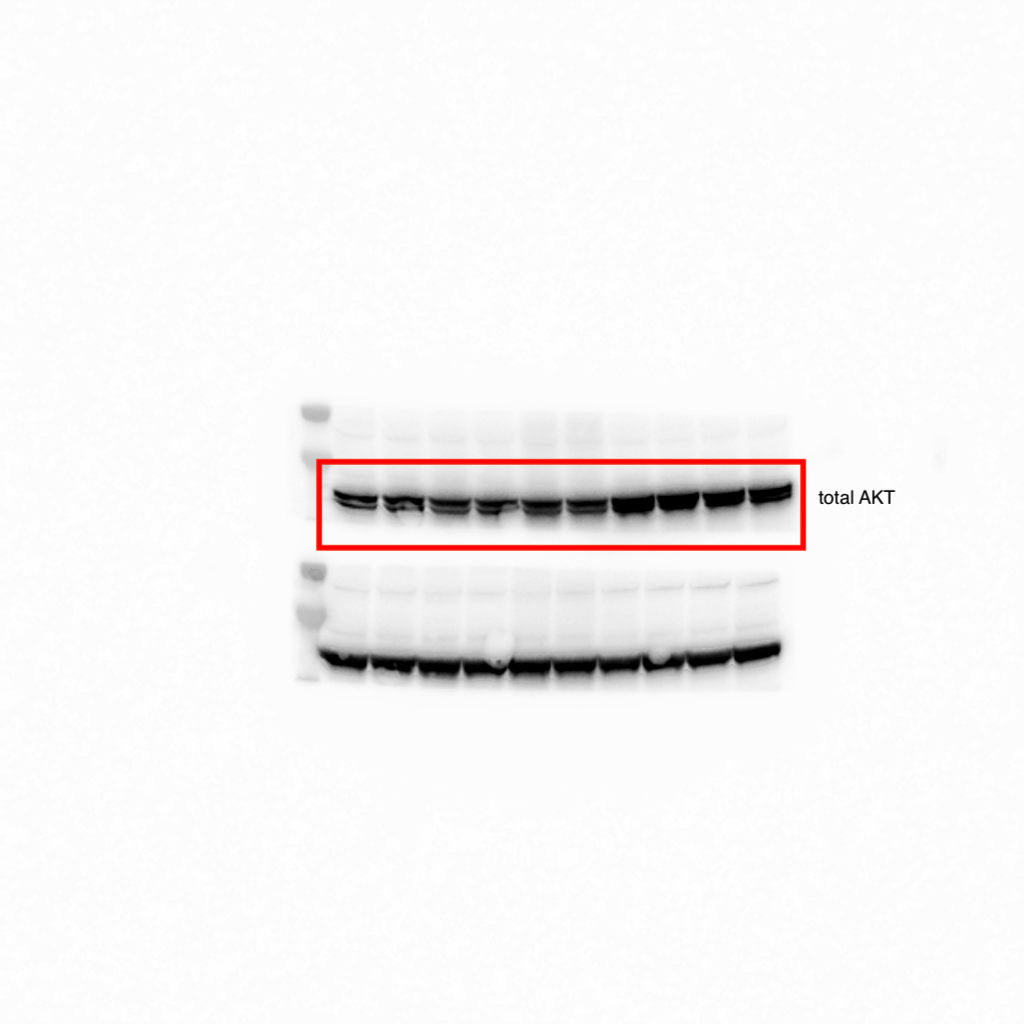

Supplement: Supplementary file 15 — Appendix and EV Figure Source Data [file 44319_2024_75_MOESM15_ESM.zip › Figure EV2/EV2B/total AKT 0.5 sec.Tif]

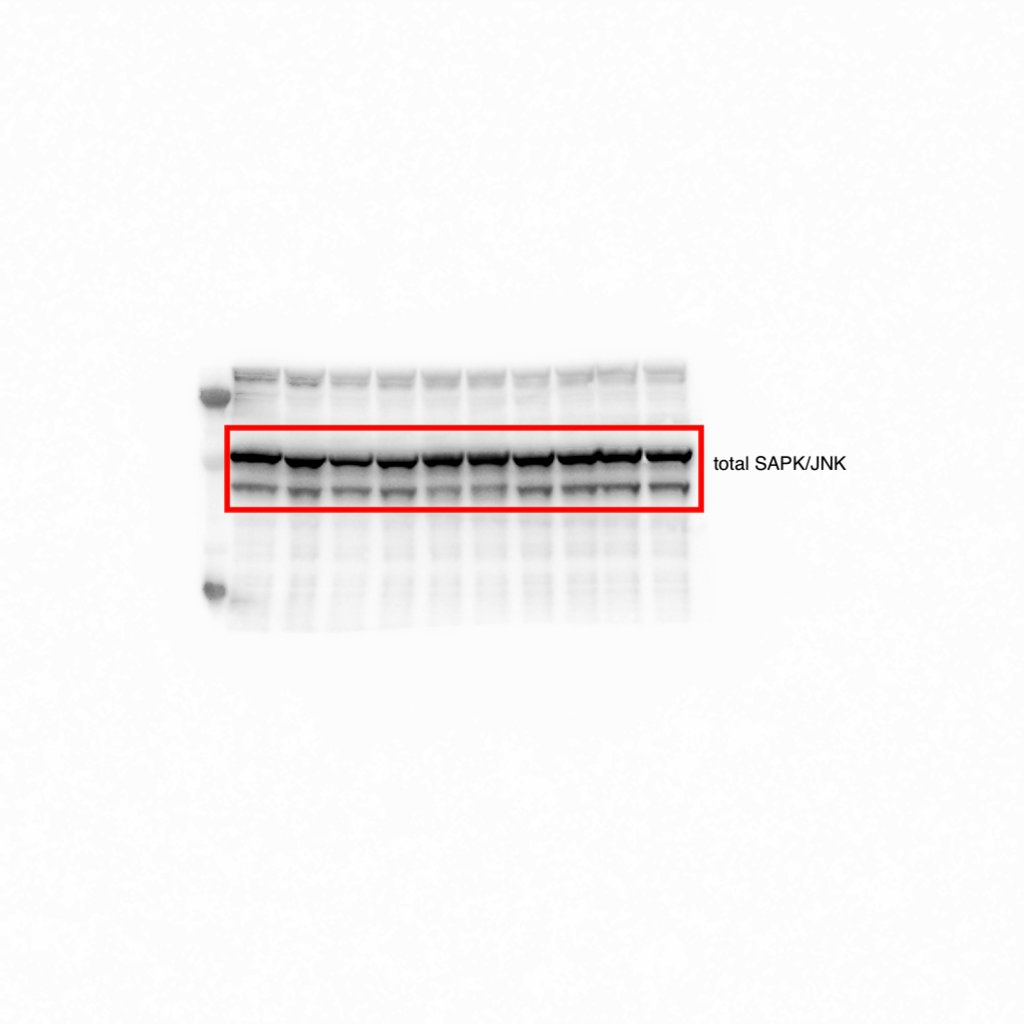

Supplement: Supplementary file 15 — Appendix and EV Figure Source Data [file 44319_2024_75_MOESM15_ESM.zip › Figure EV2/EV2B/total JNK 0.5 sec.Tif]

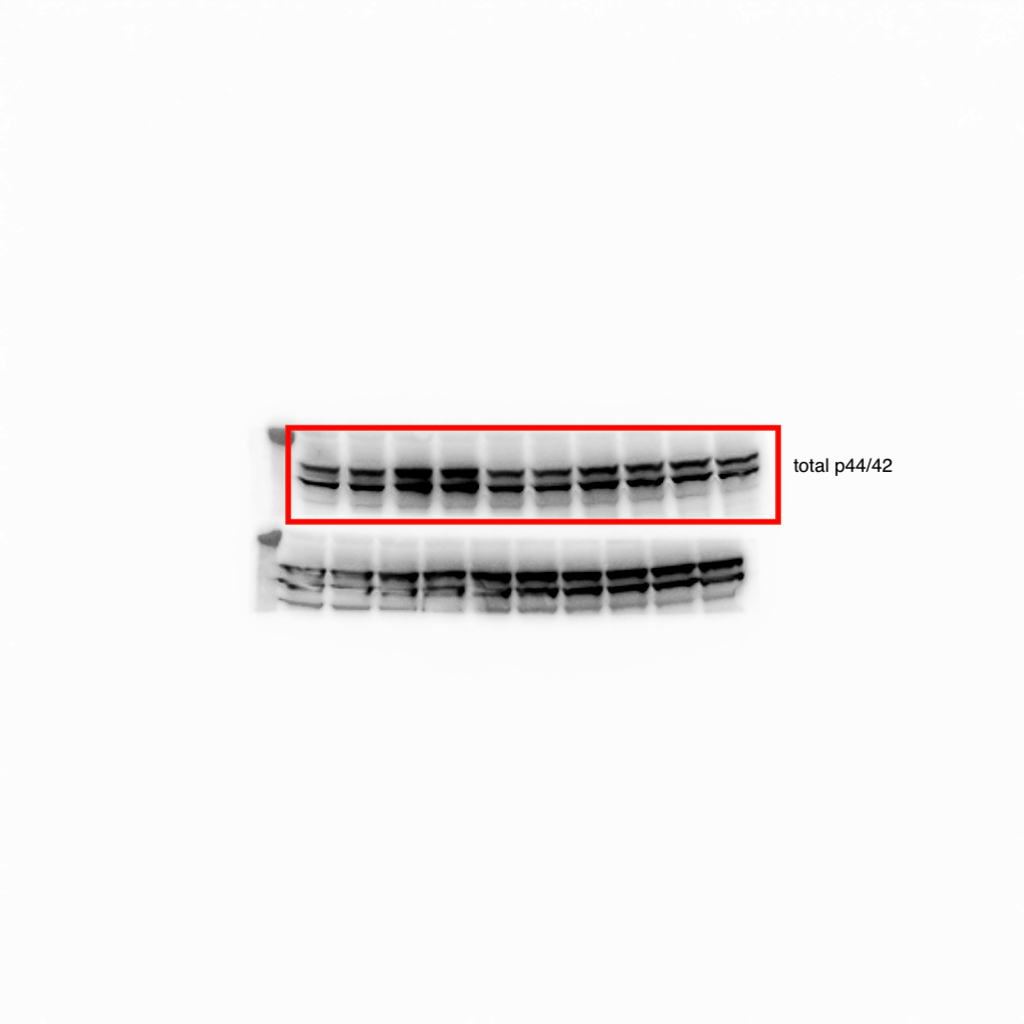

Supplement: Supplementary file 15 — Appendix and EV Figure Source Data [file 44319_2024_75_MOESM15_ESM.zip › Figure EV2/EV2B/total p44 42 0.5 sec.Tif]

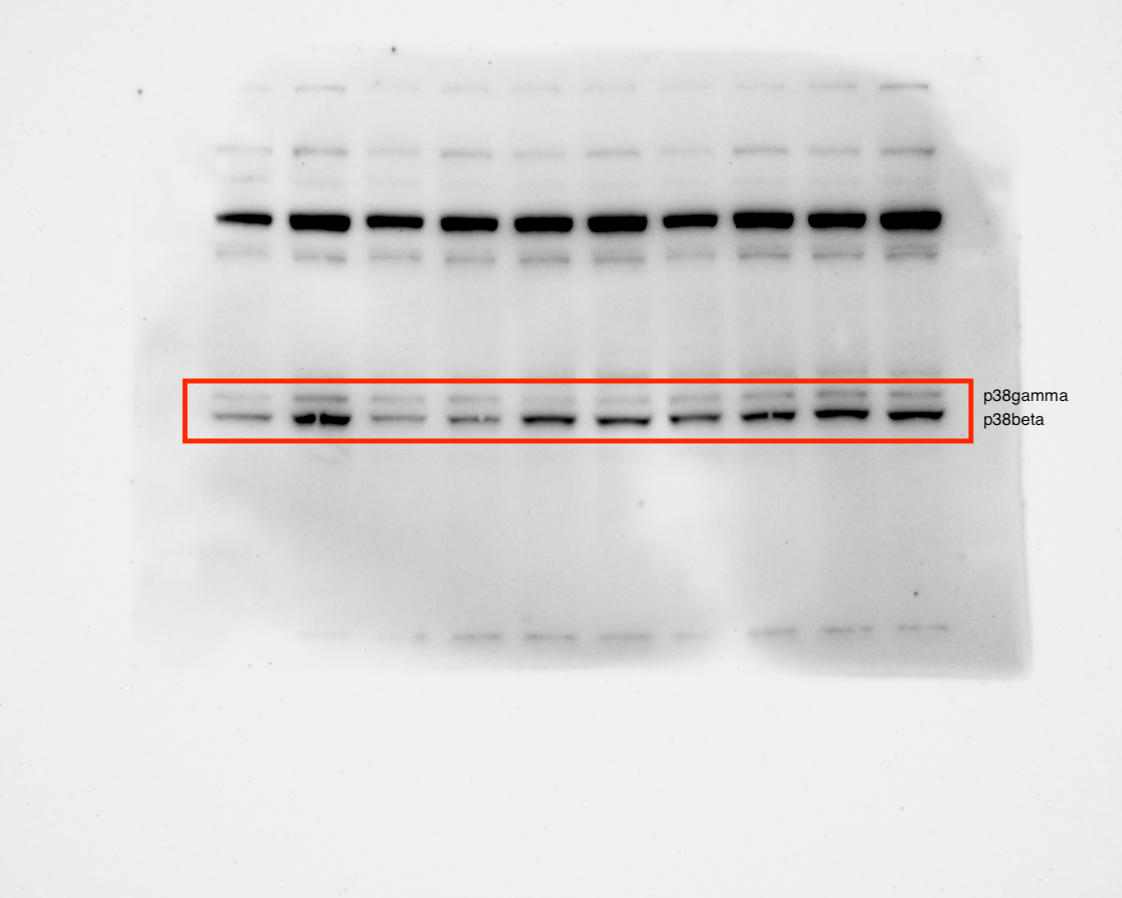

Supplement: Supplementary file 15 — Appendix and EV Figure Source Data [file 44319_2024_75_MOESM15_ESM.zip › Figure EV2/EV2C/p38beta reblot p38gamma.tif]

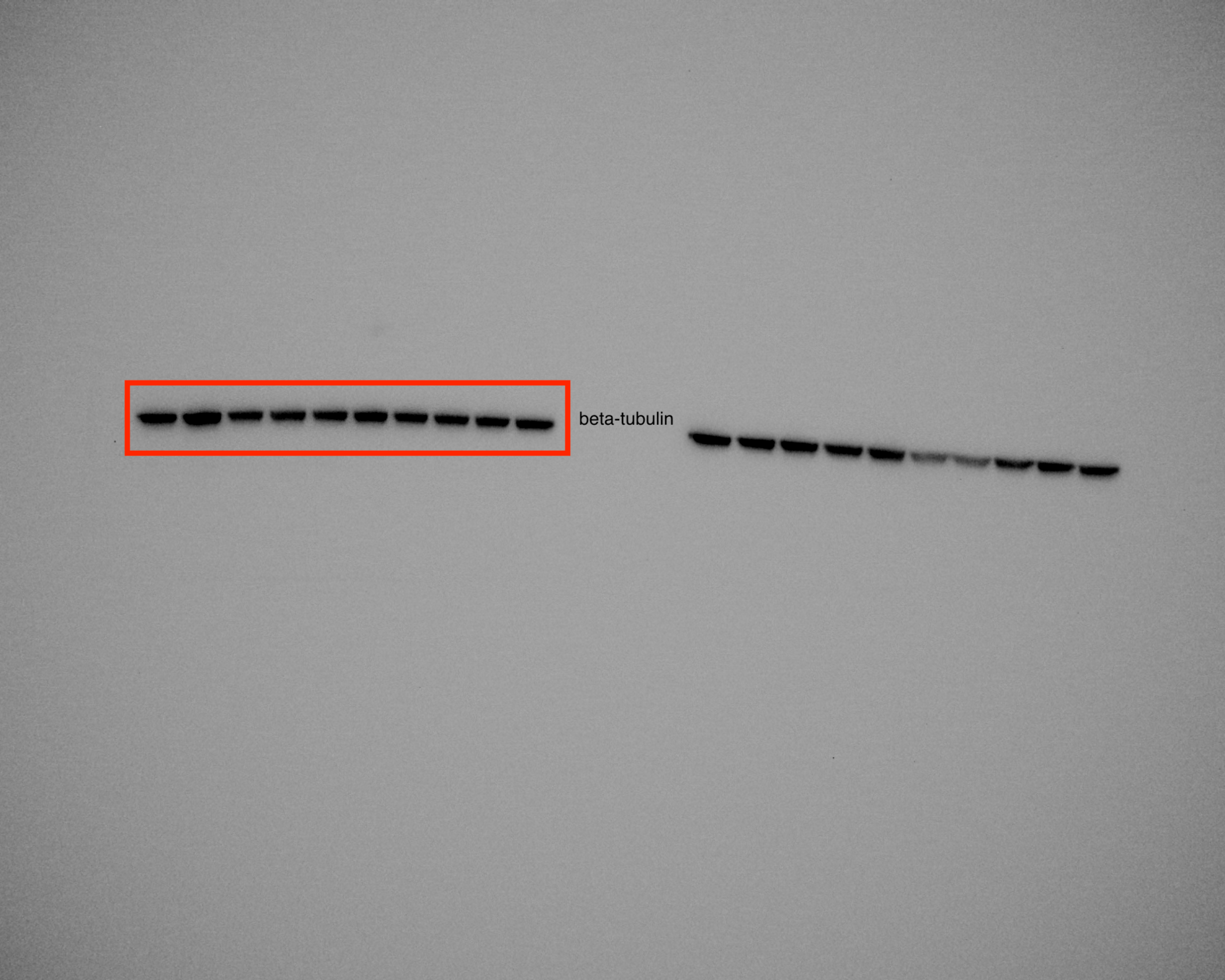

Supplement: Supplementary file 15 — Appendix and EV Figure Source Data [file 44319_2024_75_MOESM15_ESM.zip › Figure EV2/EV2C/reblot beta-tubulin.tif]

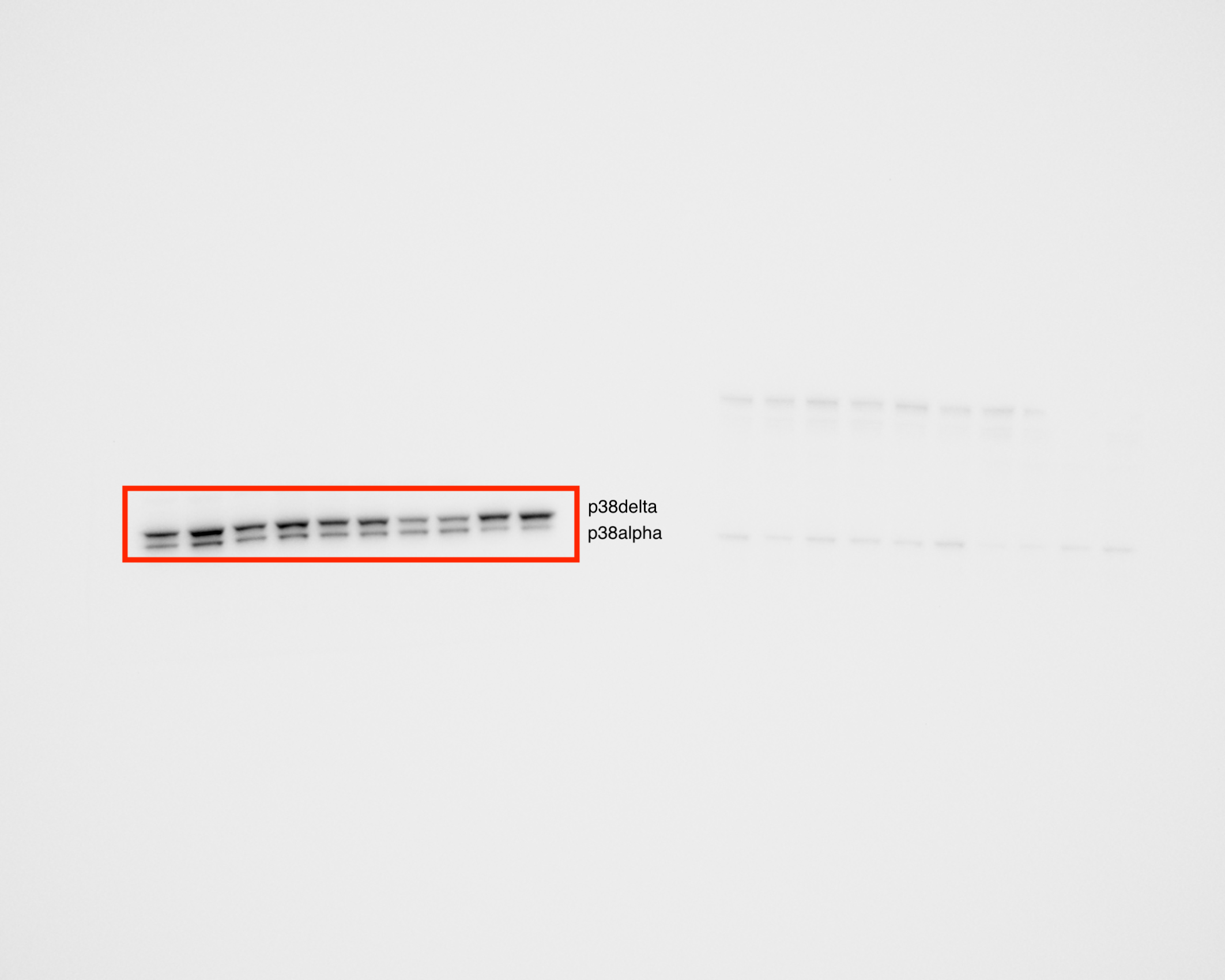

Supplement: Supplementary file 15 — Appendix and EV Figure Source Data [file 44319_2024_75_MOESM15_ESM.zip › Figure EV2/EV2C/reblot p38delta p38alpha.tif]

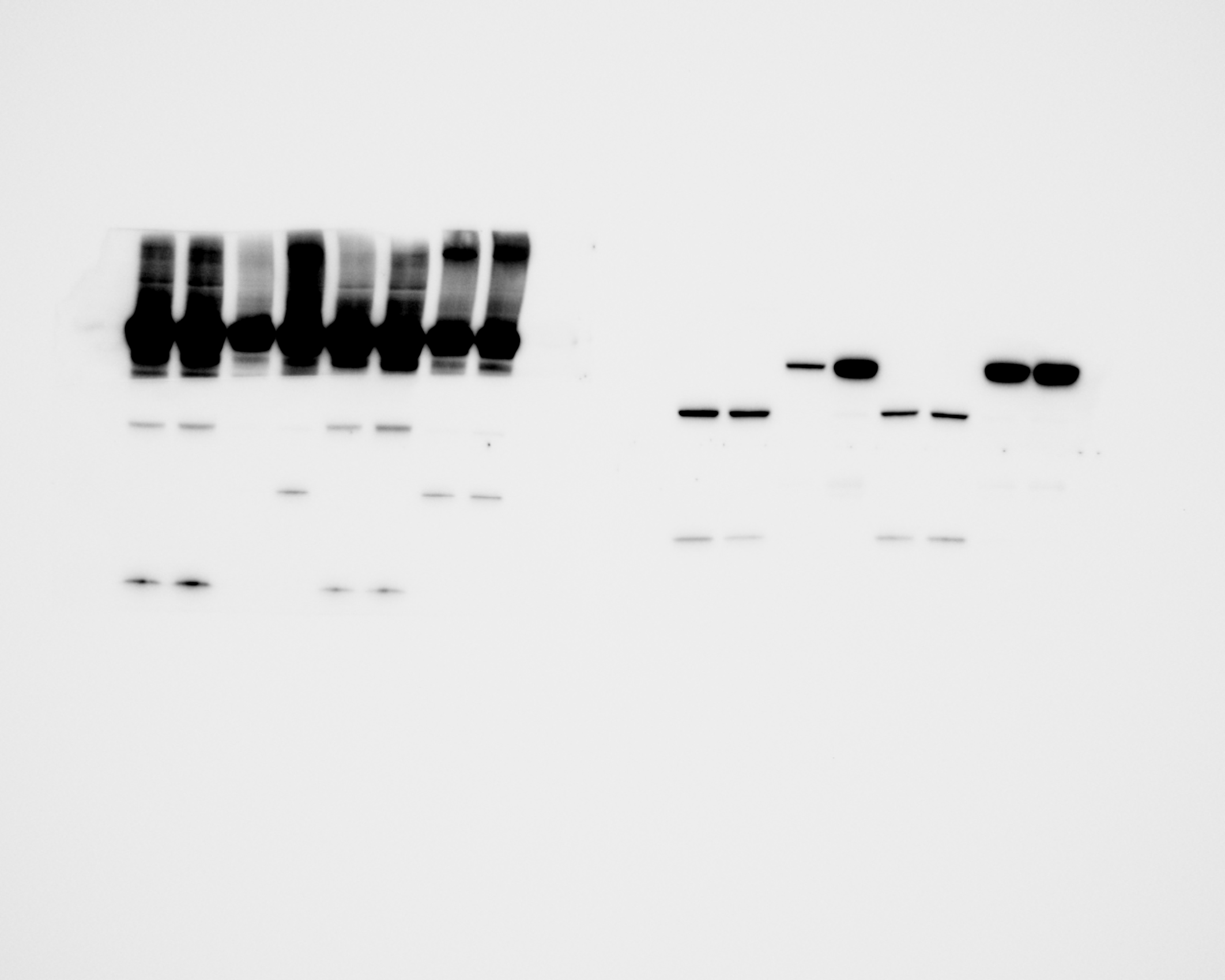

Supplement: Supplementary file 15 — Appendix and EV Figure Source Data [file 44319_2024_75_MOESM15_ESM.zip › Figure EV4/EV4D/14-3-3.tif]

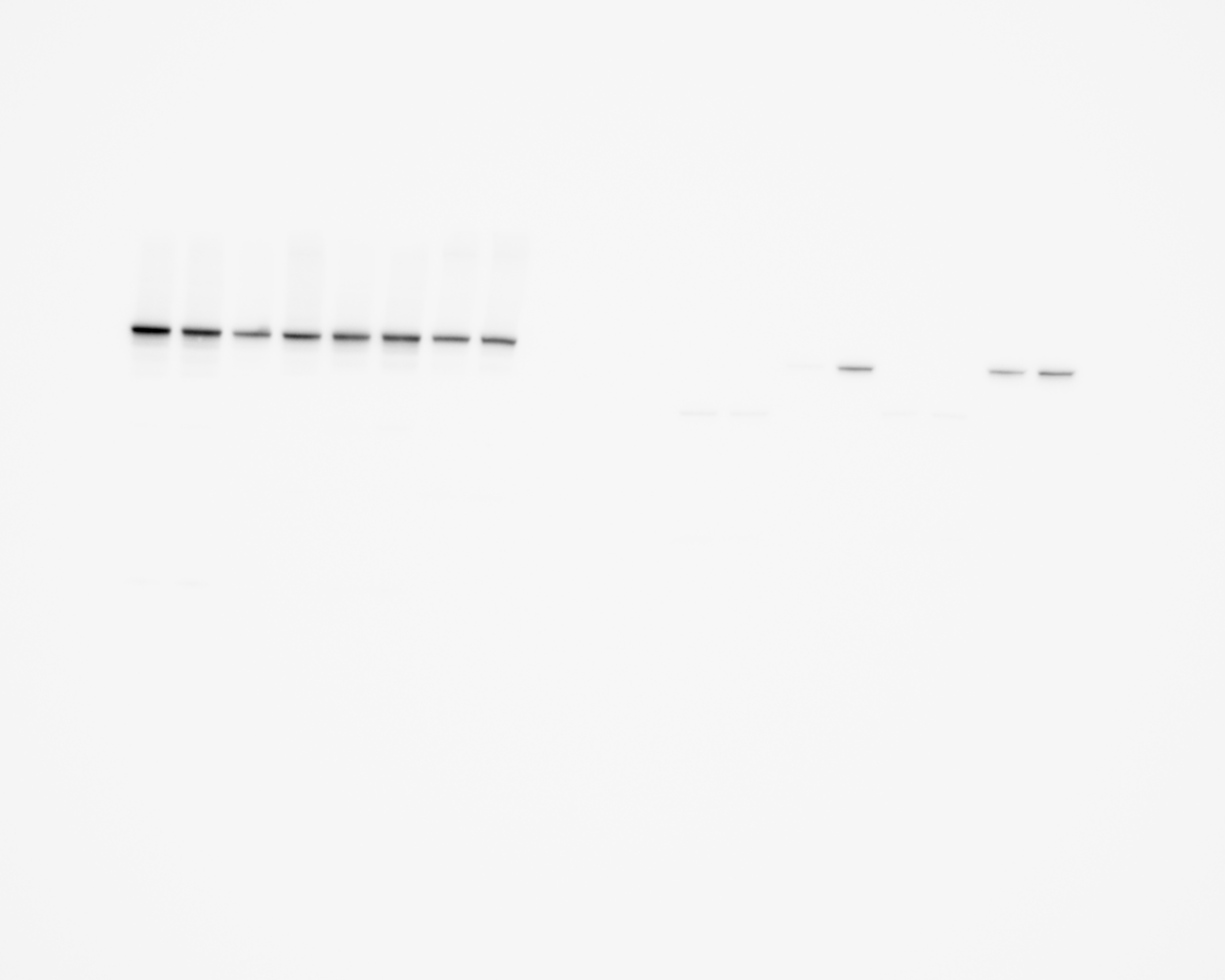

Supplement: Supplementary file 15 — Appendix and EV Figure Source Data [file 44319_2024_75_MOESM15_ESM.zip › Figure EV4/EV4D/beta-catenin.tif]

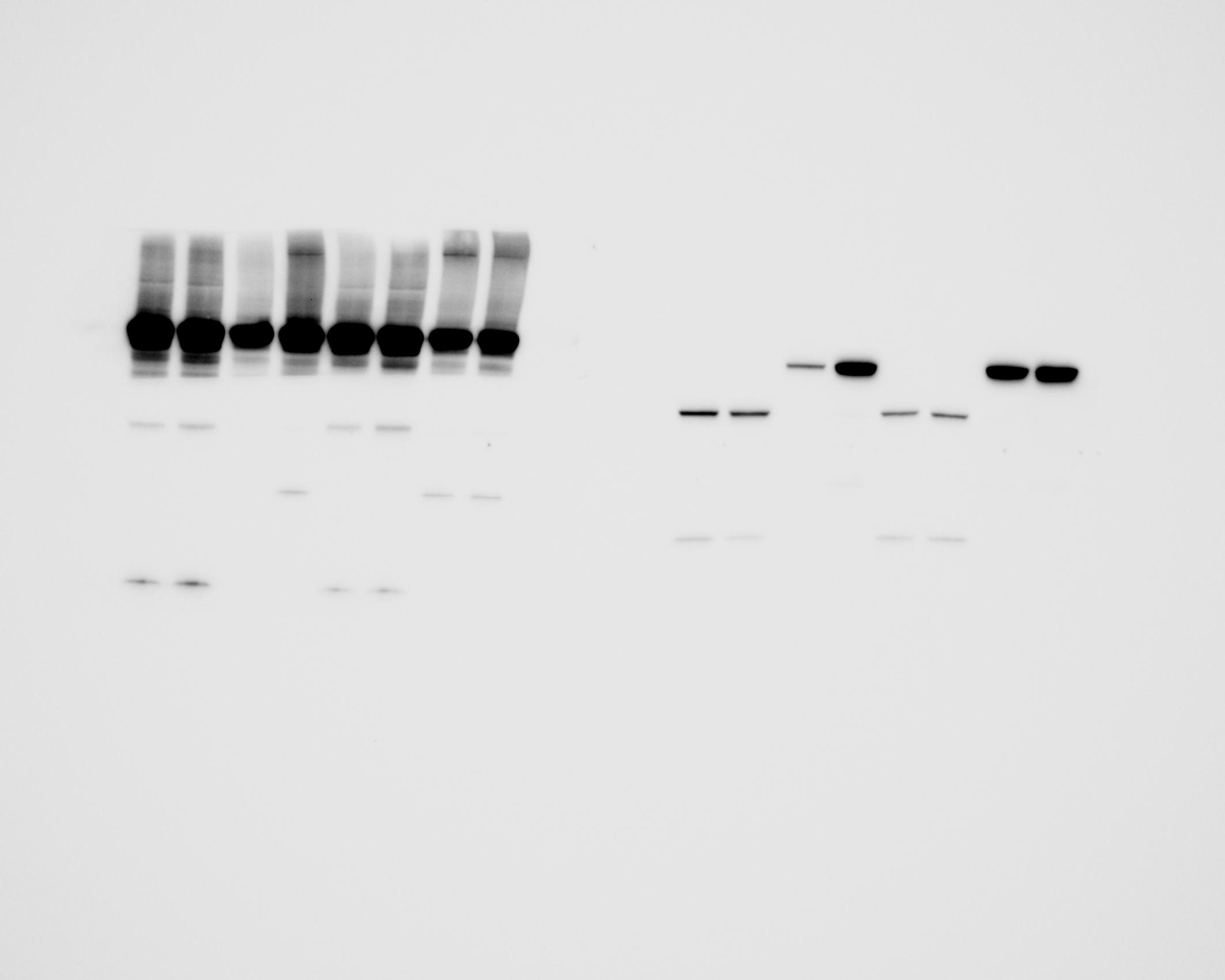

Supplement: Supplementary file 15 — Appendix and EV Figure Source Data [file 44319_2024_75_MOESM15_ESM.zip › Figure EV4/EV4D/laminB1.tif]

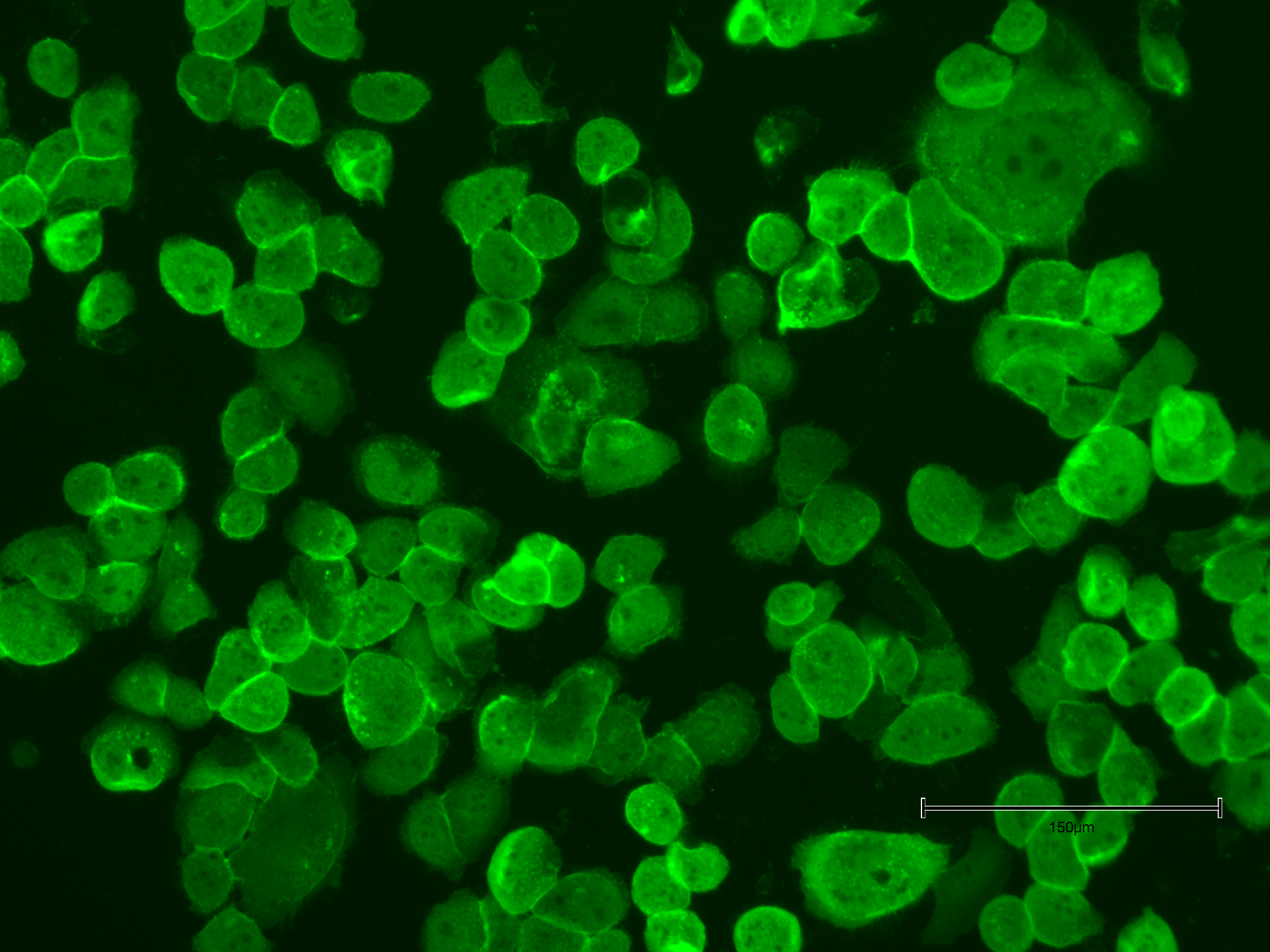

Supplement: Supplementary file 15 — Appendix and EV Figure Source Data [file 44319_2024_75_MOESM15_ESM.zip › Figure EV4/EV4E/pcDNA3+CHIR.tif]

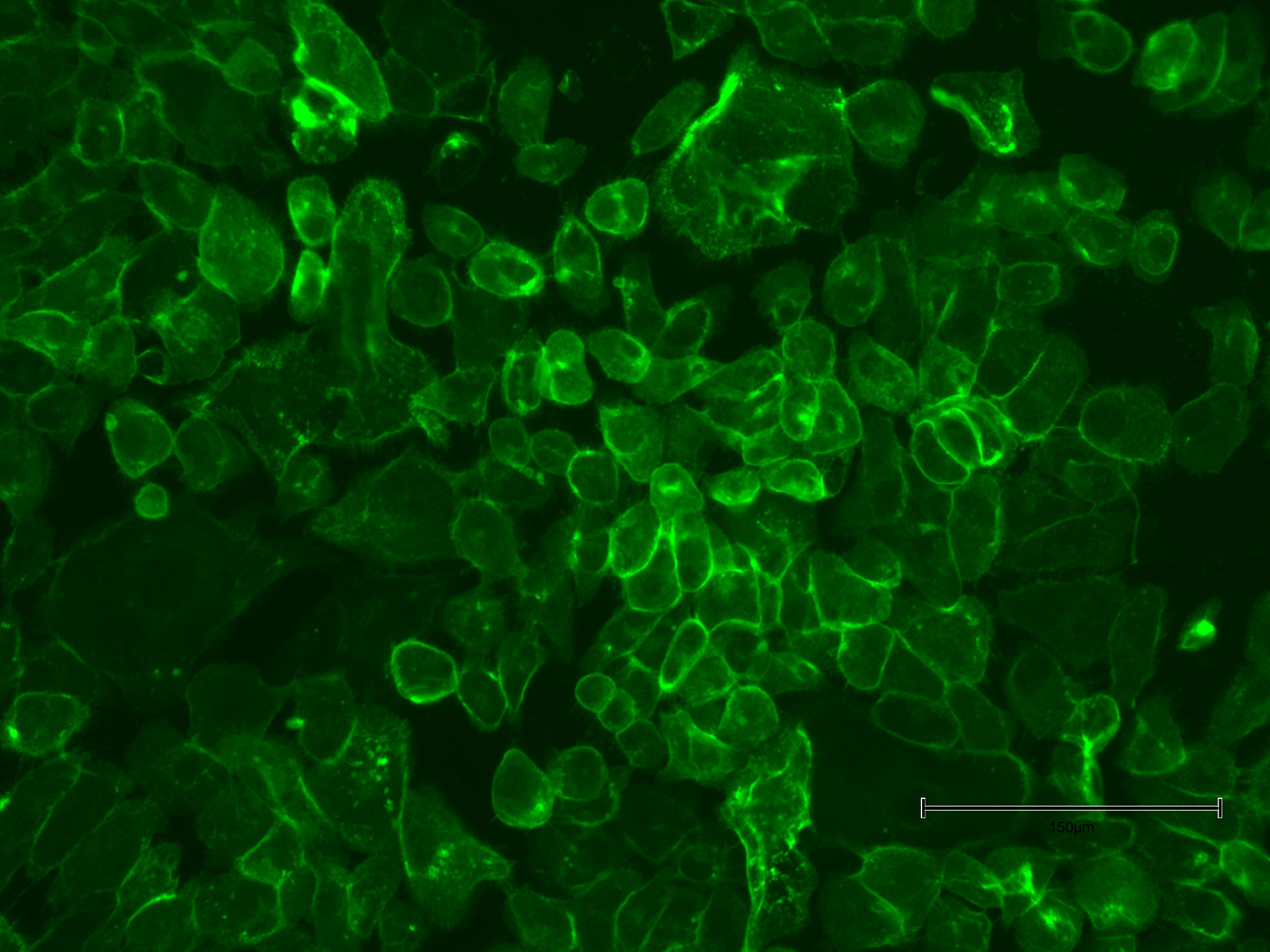

Supplement: Supplementary file 15 — Appendix and EV Figure Source Data [file 44319_2024_75_MOESM15_ESM.zip › Figure EV4/EV4E/pcDNA3-CHIR.tif]

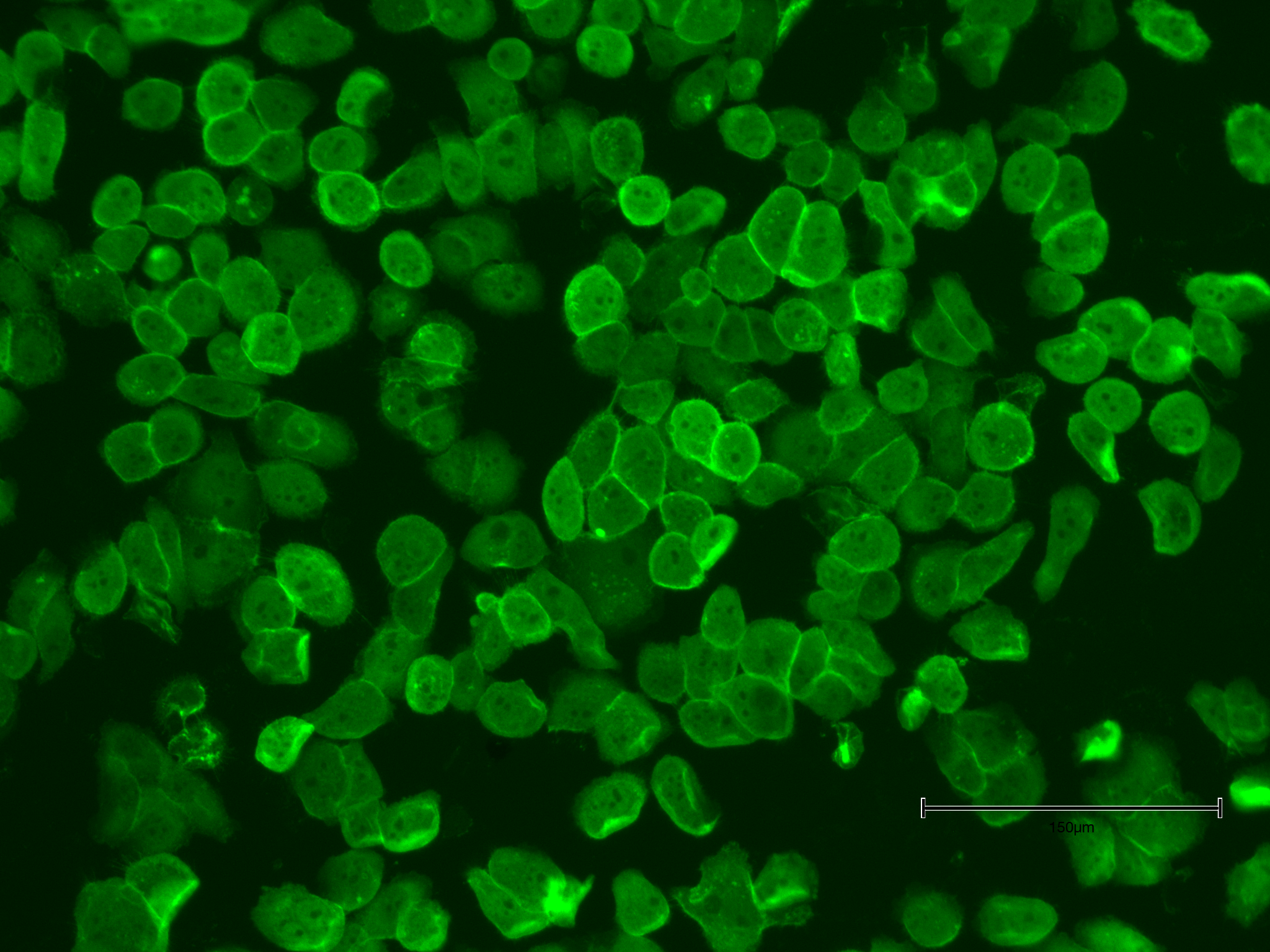

Supplement: Supplementary file 15 — Appendix and EV Figure Source Data [file 44319_2024_75_MOESM15_ESM.zip › Figure EV4/EV4E/pcLINC+CHIR.tif]

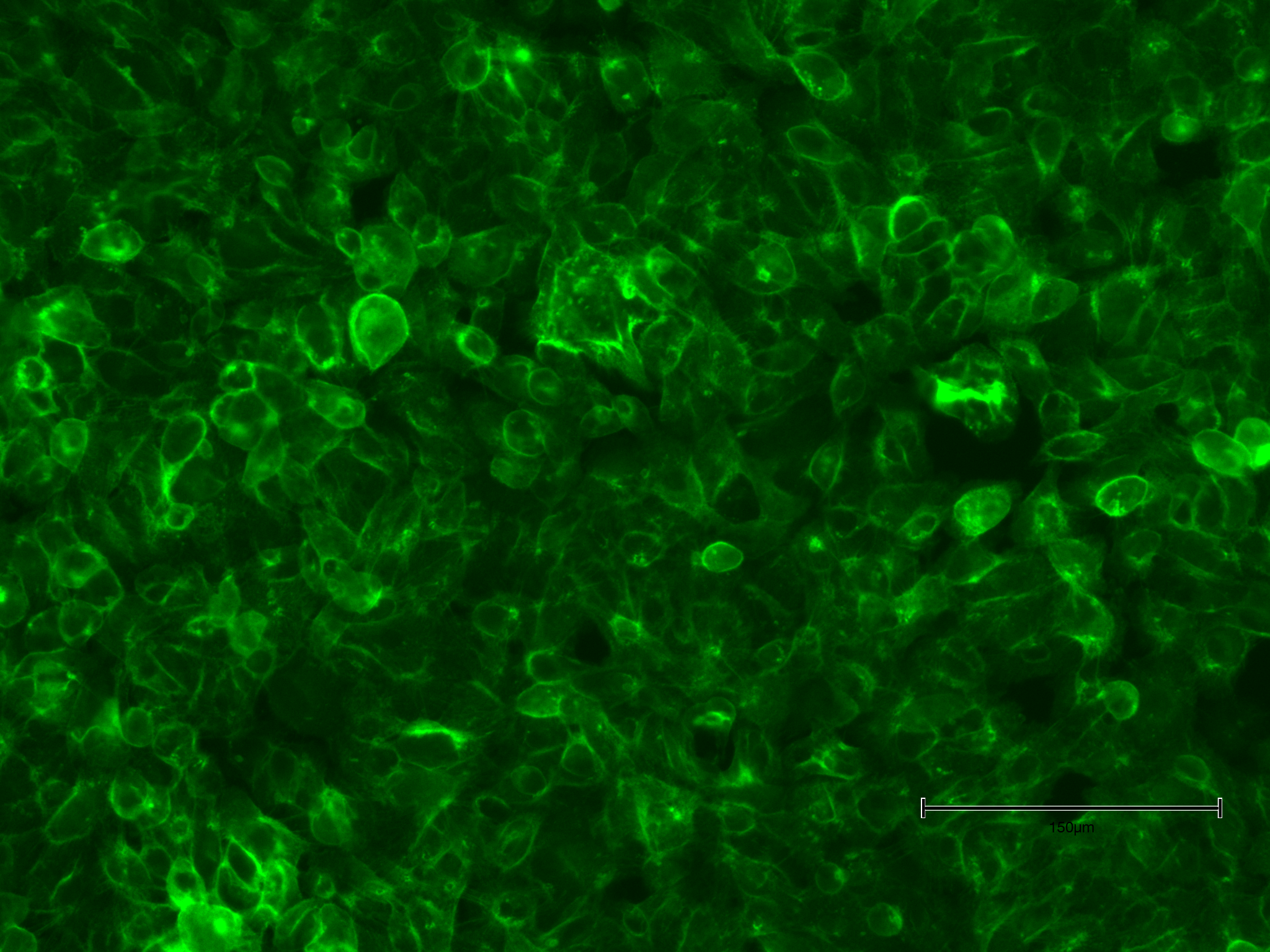

Supplement: Supplementary file 15 — Appendix and EV Figure Source Data [file 44319_2024_75_MOESM15_ESM.zip › Figure EV4/EV4E/pcLINC-CHIR.tif]
