# Supplementary material for: TGFβ-induced long non-coding RNA LINC00313 activates Wnt signaling and promotes cholangiocarcinoma
Source: EMBO Rep. 2024 Feb 8;25(3):11. doi: 10.1038/s44319-024-00075-z (PMC10933437; doi:10.1038/s44319-024-00075-z)
Supplement: Supplementary file 17 — Expanded View Figures [file 44319_2024_75_MOESM17_ESM.pdf]

Expanded View Figures

Figure EV1. Characterization of LINC00313.

(**A**) Schematic representation of LINC00313 genomic locus on chromosome 21. (**B**) LINC00313 expression profile among 27 different human tissues from 95 individuals, as determined by RNA-seq (accession: PRJEB4337). (**C**) LINC00313 expression levels among 29 cancer types from the PanCancer Atlas of TCGA, sorted from the highest (top) to the lowest (bottom) expression. (**D**) ORF analysis of LINC00313 lncRNA sequence, using the Open Reading Frame Finder tool from NCBI. (**E**) Snapshot of the LINC00313 genomic locus from the Ensembl database showing the different splice variants. Data information: In panel (**B**) LINC00313 expression is presented in Reads Per Kilobase per Million mapped reads. In panel (**C**) LINC00313 expression is quantified with RSEM and displayed in logarithmic scale.

© The Author(s)

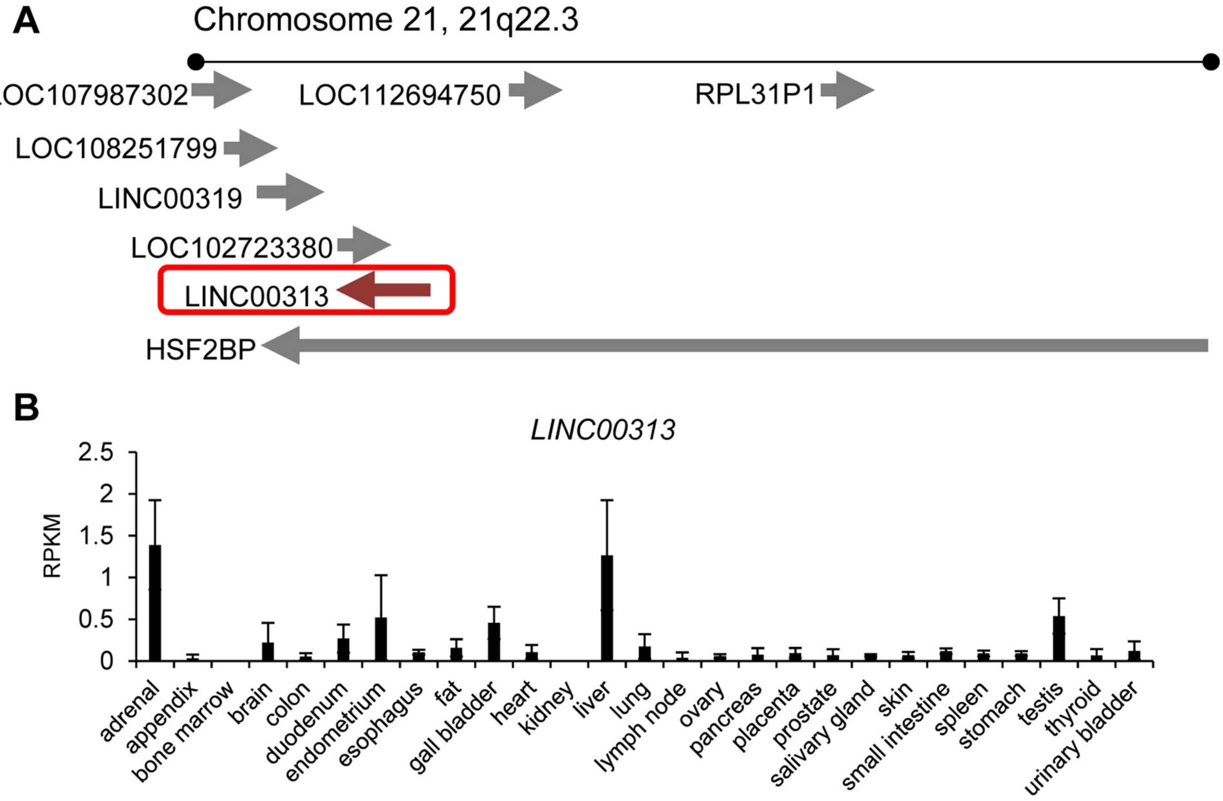

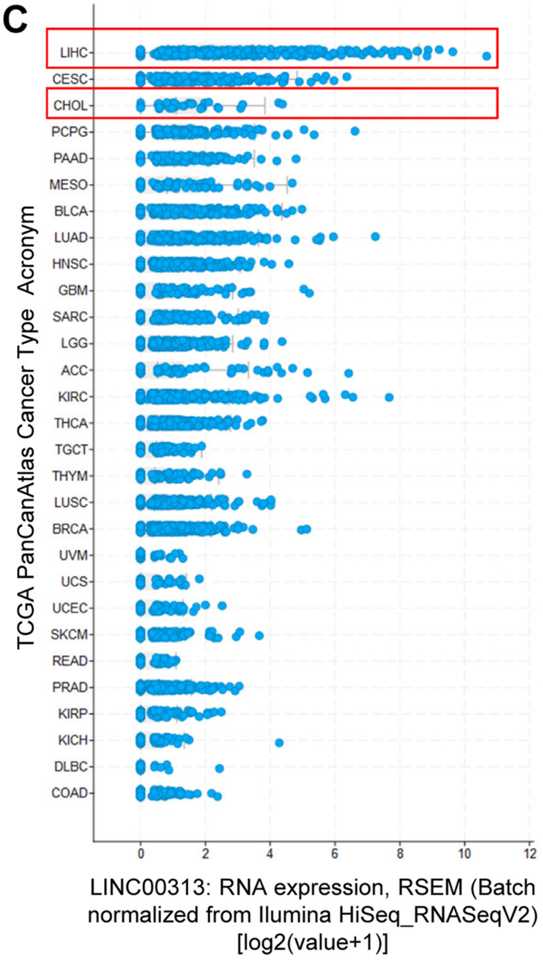

D

Label	Strand	Frame	Start	Stop	Length (nt|aa)
ORF5	-	1	306	67	240|79
ORF3	+	3	90	323	234|77
ORF6	-	2	566	390	177|58
ORF1	+	1	277	423	147|48
ORF4	+	3	369	488	120|39
ORF2	+	2	287	400	114|37
ORF7	-	3	91	>2	90|29

E

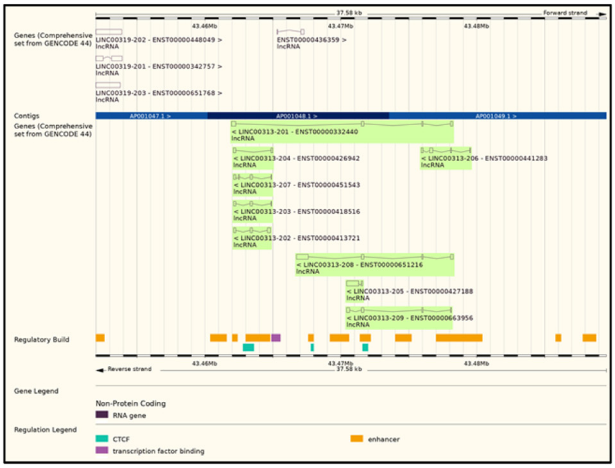

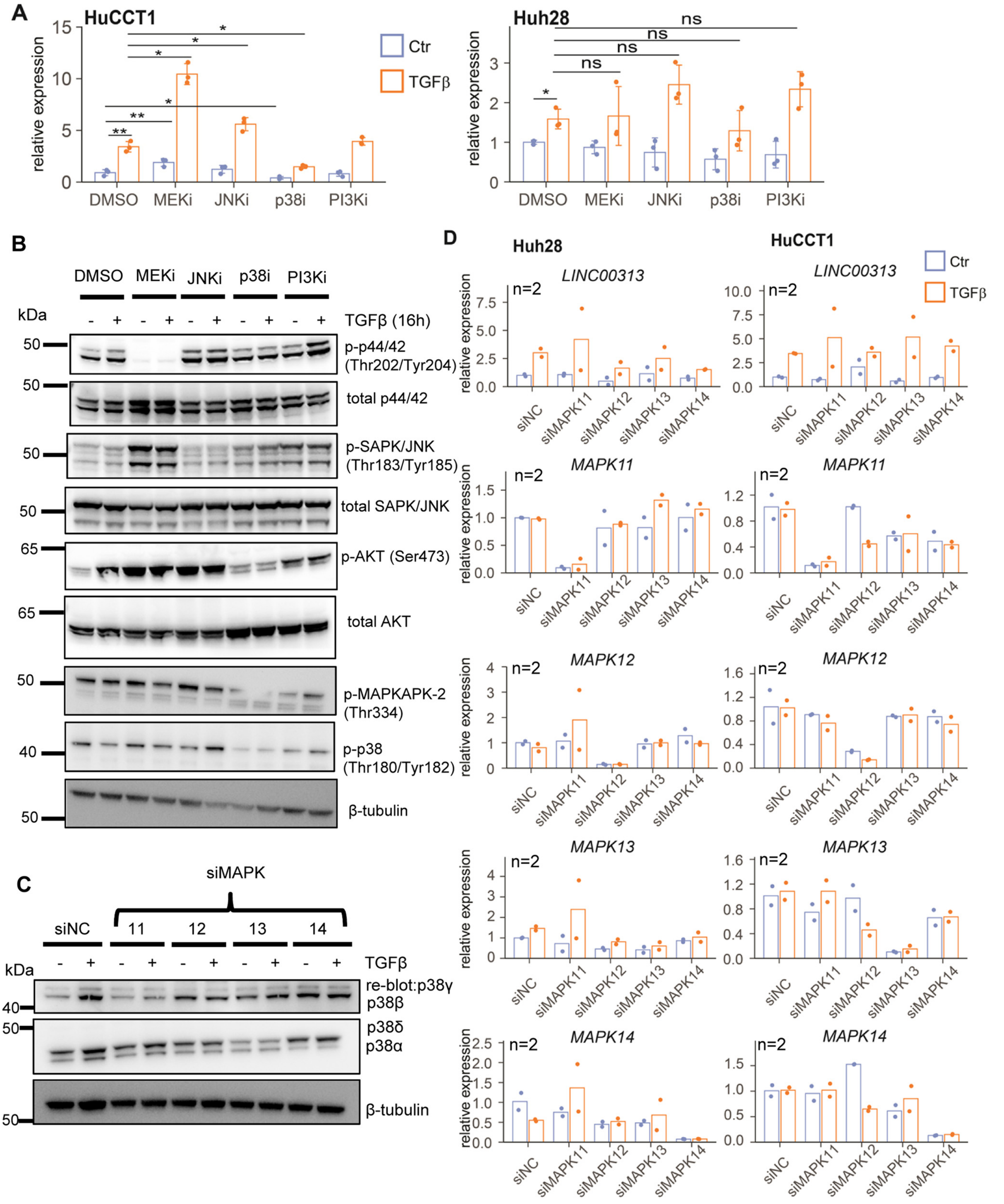
A
HuCCT1
relative expression
Ctr
TGFβ
DMSO
MEKi
JNKi
p38i
PI3Ki
Huh28
ns
B
DMSO
MEKi
JNKi
p38i
PI3Ki
kDa
TGFβ (16h)
p-p44/42
(Thr202/Tyr204)
total p44/42
p-SAPK/JNK
(Thr183/Tyr185)
total SAPK/JNK
p-AKT (Ser473)
total AKT
p-MAPKAPK-2
(Thr334)
p-p38
(Thr180/Tyr182)
β-tubulin
C
siMAPK
siNC
11
12
13
14
TGFβ
re-blot:p38γ
p38β
p38δ
p38α
β-tubulin
D
Huh28
HuCCT1
LINC00313
MAPK11
MAPK12
MAPK13
MAPK14
n=2
siNC
siMAPK11
siMAPK12
siMAPK13
siMAPK14

Figure EV2. Effect of the non-canonical TGFβ pathway on LINC00313 expression.

(**A**) *LINC00313* levels in HuCCT1 or Huh28 treated with MEK, p38, JNK or PI3K inhibitors, with or without TGFβ1 stimulation for 16 h ($n = 3$ biological replicates). (**B**) Immunoblotting for detection of phosphorylated p44/42 (Thr202/Tyr204), phosphorylated p-SAPK/JNK (Thr183/Tyr185), phosphorylated AKT (Ser473), phosphorylated p38 (Thr180/Tyr182), phosphorylated MAPKAPK-2 (Thr334), total p44/42, SAPK/JNK and AKT protein levels and β-tubulin (used as a loading control) in HuCCT1 cells treated with MEK, p38, JNK or PI3K inhibitors, with or without TGFβ1 stimulation for 16 h. DMSO was used as a vehicle treatment. (**C**) Immunoblotting for detection of the p38 isoforms p38α, p38β, p38γ and p38δ in HuCCT1 cells transiently silenced for *MAPK11* or *MAPK12* or *MAPK13* or *MAPK14* and treated or not with TGFβ1 for 16 h. β-tubulin was used as a loading control. (**D**) Real-time qPCR analysis of *LINC00313*, *MAPK11*, *MAPK12*, *MAPK13* and *MAPK14* expression in HuCCT1 and Huh28 cell lines transiently transfected with siRNAs targeting *MAPK11* or *MAPK12* or *MAPK13* or *MAPK14* and treated or not with TGFβ1 for 16 h. Data information: In Panel (**A**) data are presented as mean ± SD ($n = 3$ biological replicates). In panel (**D**) data are presented as single data points ($n = 2$ biological replicates). *$P \leq 0.05$, **$P \leq 0.01$, n.s.: not significant (Student's *t*-test). Source data are available online for this figure.

© The Author(s)

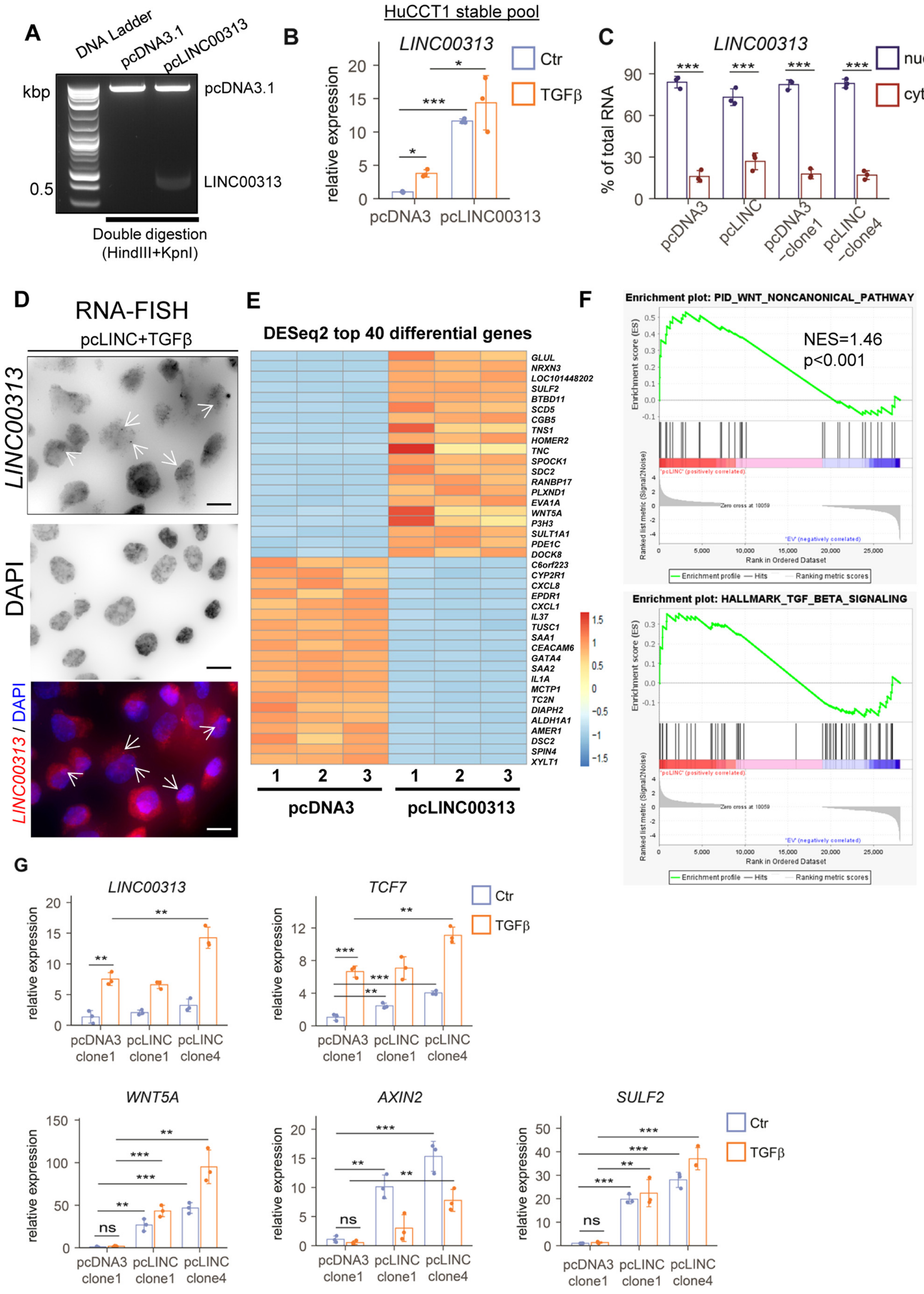
A
DNA Ladder
pcDNA3.1
pcLINC00313
kbp
0.5
pcDNA3.1
LINC00313
Double digestion
(HindIII+KpnI)
B
HuCCT1 stable pool
LINC00313
Ctr
TGFβ
relative expression
pcDNA3
pcLINC00313
C
LINC00313
nuclear
cyto
% of total RNA
pcDNA3
pcLINC
pcDNA3 -clone1
pcLINC -clone4
D
RNA-FISH
pcLINC+TGFβ
LINC00313
DAPI
LINC00313 / DAPI
E
DESeq2 top 40 differential genes
GLUL
NRXN3
LOC101448202
SULF2
BTBD11
SCD5
CGB5
TNS1
HOMER2
TNC
SPOCK1
SDC2
RANBP17
PLXND1
EVA1A
WNT5A
P3H3
SULT1A1
PDE1C
DOCK8
C6orf223
CYP2R1
CXCL8
EPDR1
CXCL1
IL37
TUSC1
SAA1
CEACAM6
GATA4
SAA2
IL1A
MCTP1
TC2N
DIAPH2
ALDH1A1
AMER1
DSC2
SPIN4
XYLT1
pcDNA3
pcLINC00313
F
Enrichment plot: PID_WNT_NONCANONICAL_PATHWAY
NES=1.46
p<0.001
Enrichment score (ES)
Ranked list metric (Signal2Noise)
Rank in Ordered Dataset
Enrichment profile — Hits Ranking metric scores
Enrichment plot: HALLMARK_TGF_BETA_SIGNALING
G
LINC00313
TCF7
WNT5A
AXIN2
SULF2
Ctr
TGFβ
relative expression
pcDNA3 clone1
pcLINC clone1
pcLINC clone4
ns

 © The Author(s)

Figure EV3. ***LINC00313*** **gain of function up-regulates Wnt-related genes.**

(**A**) Efficient ligation of LINC00313 insert to pcDNA3.1 plasmid vector was verified by double digestion with restriction enzymes, followed by agarose gel electrophoresis. Molecular size (in kbp) marker ladder is shown in the first lane. (**B**) Real-time qPCR analysis of *LINC00313* expression in HuCCT1 cells stably over-expressing pcDNA3.1 or pcLINC00313 expression vectors stimulated or not with TGFβ1 for 16 h. (**C**) Subcellular fractionation followed by RT-qPCR analysis of LINC00313 RNA levels in nuclear and cytoplasmic fractions of HuCCT1 cells transiently over-expressing an empty vector (pcDNA3.1) or LINC00313 and HuCCT1 clones, stably over-expressing pcDNA3.1 or LINC00313. (**D**) smiFISH for LINC00313 RNA in HuCCT1 cells stably over-expressing LINC00313 and stimulated with TGFβ for 16 h. DAPI was used to stain nuclei. Scale bars: 15 μm. (**E**) Heatmap presenting the top 40 differentially regulated genes in HuCCT1 pcLINC00313 versus pcDNA3-expressing cells (shrunken log2FC > 1, adjusted *p*-value < 0.1). Biological triplicates (1-3) were used per condition. (**F**) GSEA analysis of differentially expressed genes in pcLINC00313 versus pcDNA3 HuCCT1 cells. (**G**) Real-time qPCR analysis of *LINC00313*, *TCF7*, *WNT5A*, *AXIN2*, and *SULF2* expression in a monoclonal HuCCT1 cell line stably expressing pcDNA3.1 and in two monoclonal HuCCT1 cell lines stably expressing pcLINC00313, in the presence or not of TGFβ1 for 16 h. Data information: Data in panels (**B**), (**C**) and (**G**) are presented as mean ± SD ($n = 3$ biological replicates). *$P \leq 0.05$, **$P \leq 0.01$, ***$P \leq 0.001$ (Student's *t*-test). In panel (**F**) the Kolmogorov-Smirnov test was used for statistical analysis of GSEA. Source data are available online for this figure.

© The Author(s)

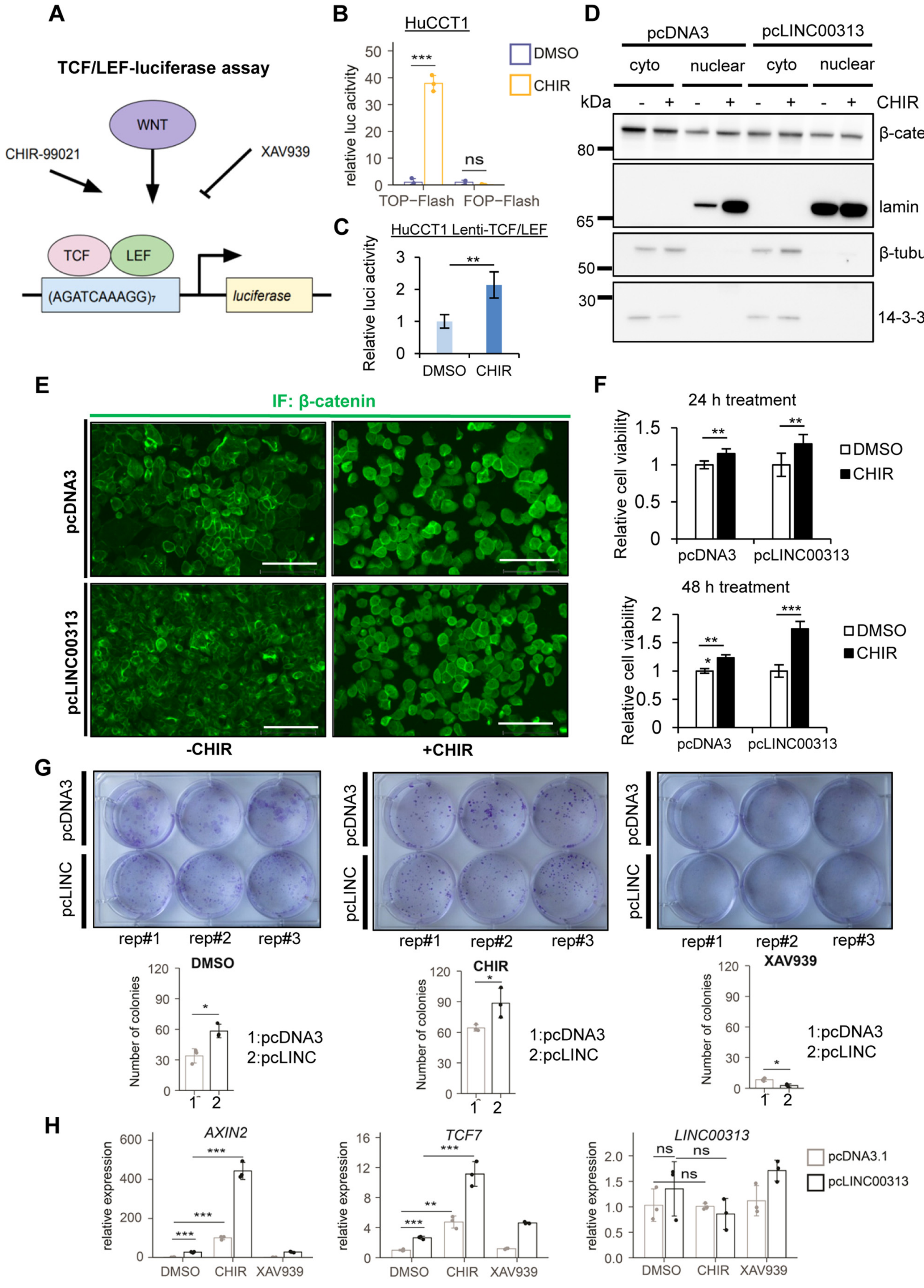
A
TCF/LEF-luciferase assay
WNT
CHIR-99021
XAV939
TCF
LEF
(AGATCAAAGG)7
luciferase
B
HuCCT1
relative luc acitvity
DMSO
CHIR
***
ns
TOP−Flash FOP−Flash
C
HuCCT1 Lenti-TCF/LEF
Relative luci activity
**
DMSO CHIR
D
pcDNA3
pcLINC00313
cyto nuclear cyto nuclear
kDa
CHIR
β-catenin
lamin B1
β-tubulin
14-3-3
E
IF: β-catenin
pcDNA3
pcLINC00313
-CHIR
+CHIR
F
24 h treatment
48 h treatment
Relative cell viability
DMSO
CHIR
pcDNA3 pcLINC00313
G
pcDNA3
pcLINC
rep#1 rep#2 rep#3
DMSO
CHIR
XAV939
Number of colonies
1:pcDNA3
2:pcLINC
H
AXIN2
TCF7
LINC00313
relative expression
DMSO CHIR XAV939
pcDNA3.1
pcLINC00313

 © The Author(s)

Figure EV4. *LINC00313* modulates chemically-induced Wnt/β-catenin signalling and impacts physiological processes in CCA.

(**A**) Schematic representation of the TCF/LEF-luciferase reporter assay. Activators (Wnt ligands, CHIR) and inhibitors (XAV939) of TCF/LEF-dependent responses are also shown. (**B**) TCF/LEF-luciferase reporter assay in HuCCT1 cells transiently transfected with TOP-Flash or FOP-Flash luciferase expression vectors and treated with CHIR99021 or DMSO for 24 h. Cells were co-transfected with a Renilla luciferase expression vector for normalization of the firefly luciferase activity. (**C**) TCF/LEF-luciferase reporter assay in HuCCT1 cells stably expressing the pGreenFire 2.0 TCF/LEF reporter construct and treated with CHIR99021 or DMSO for 24 h. TCF/LEF-luciferase reporter assay in HuCCT1 cells stably expressing the pGreenFire 2.0 TCF/LEF reporter construct and treated with the indicated concentrations of TGFβ1 or BSA/HCl (Ctr) for 16 h. (**D**) Subcellular fractionation followed by immunoblotting for β-catenin, lamin B1 (nuclear marker), β-tubulin and 14-3-3 (cytoplasmic markers) using nuclear and cytoplasmic fractions of HuCCT1 cells stably over-expressing an empty vector (pcDNA3.1) or LINC00313 and treated with CHIR99021 or DMSO for 24 h. (**E**) Immunofluorescence to detect β-catenin subcellular localization in control or LINC00313 over-expressing HuCCT1 cells, treated or not with CHIR99021 or DMSO for 24 h. Scale bars: 150 μm. (**F**) Cell viability assays in control or LINC00313 over-expressing HuCCT1 cells treated with CHIR99021 or DMSO for 24 h. (**G**) Colony formation assay in control or LINC00313 over-expressing HuCCT1 cells, treated with CHIR99021 or XAV939 or DMSO for 24 h. Quantification of the number of colonies for each condition is also shown. (**H**) Real-time qPCR analysis of *AXIN2*, *TCF7* and *LINC00313* expression in control or LINC00313 overexpressing HuCCT1 cells, treated with CHIR99021, XAV939, or DMSO for 24 h. Data information: In panel (**B**) data are presented as mean ± SD ($n = 3$ biological replicates). In panel (**F**) data are presented as mean ± SD ($n = 6$ biological replicates). $^{**}P \le 0.01$, $^{***}P \le 0.001$ (Student's *t*-test). In panels (**G**) and (**H**) data are presented as mean ± SD ($n = 3$ biological replicates). $^{*}P \le 0.05$, $^{**}P \le 0.01$, $^{***}P \le 0.001$ (Student's *t*-test). Source data are available online for this figure.

© The Author(s)

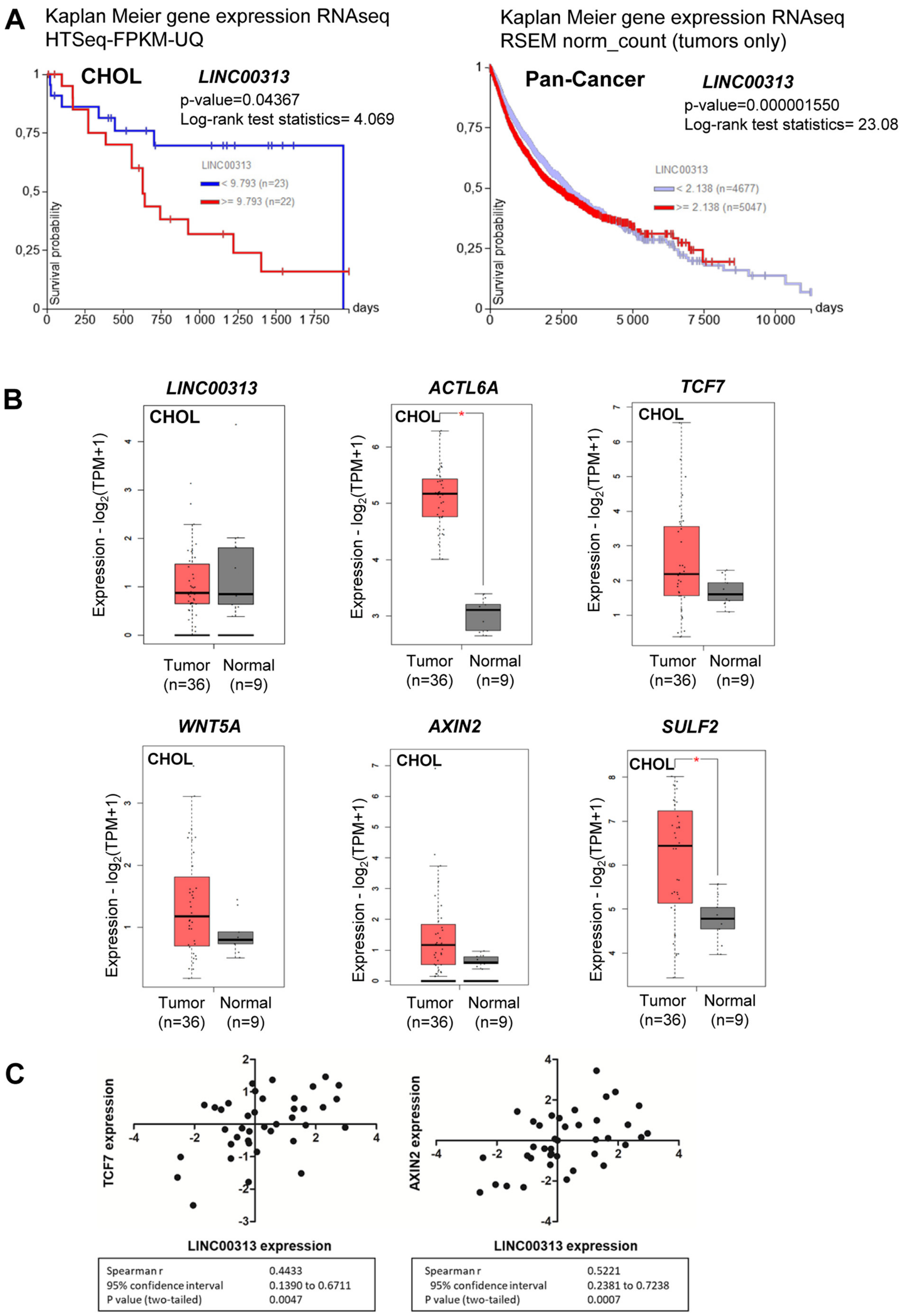
A
Kaplan Meier gene expression RNAseq HTSeq-FPKM-UQ
CHOL
LINC00313
p-value=0.04367
Log-rank test statistics= 4.069
LINC00313
< 9.793 (n=23)
>= 9.793 (n=22)
Survival probability
days
Kaplan Meier gene expression RNAseq RSEM norm_count (tumors only)
Pan-Cancer
LINC00313
p-value=0.000001550
Log-rank test statistics= 23.08
LINC00313
< 2.138 (n=4677)
>= 2.138 (n=5047)
Survival probability
days
B
LINC00313
ACTL6A
TCF7
WNT5A
AXIN2
SULF2
CHOL
Expression - log2(TPM+1)
Tumor (n=36)
Normal (n=9)
C
TCF7 expression
LINC00313 expression
Spearman r 0.4433
95% confidence interval 0.1390 to 0.6711
P value (two-tailed) 0.0047
AXIN2 expression
LINC00313 expression
Spearman r 0.5221
95% confidence interval 0.2381 to 0.7238
P value (two-tailed) 0.0007

Figure EV5. Clinical relevance of LINC00313 in CCA.

(**A**) Kaplan Meier curves depicting overall survival in CHOL patients and Pan-Cancer patients grouped based on *LINC00313* median expression, using the Xena Functional Genomics Explorer from the UCSC. (**B**) Boxplots of LINC00313, ACTL6A, TCF7, WNT5A, AXIN2 and SULF2 transcripts per million (TPMs) in cholangiocarcinoma (CHOL) tumours ($n = 36$) and their paired normal tissues ($n = 9$), using GEPIA2. $*p < 0.01$. (**C**) Expression correlation of *LINC00313* with *TCF7* and *AXIN2* in a cohort of 39 iCCA patients $p < 0.05$. Data information: In panel (**A**), log-rank test was used to compare the different Kaplan–Meier curves. In panel (**B**), transcript expression is shown in logarithmic scale and the method for differential analysis is one-way ANOVA, using disease state (tumour vs normal) as variable for calculating differential expression. The $\log_2$FC cut-off equals 1 and the *p*-value cut-off equals 0.01. In panel (**C**) a two-tailed Student's *t*-test was used to calculate *p*-values.
